# On the Nature of the Rotational Energy Barrier of Atropisomeric Hydrazides

**DOI:** 10.3390/molecules28237856

**Published:** 2023-11-29

**Authors:** Andrea Pellegrini, Laura Marcon, Paolo Righi, Giovanni Centonze, Chiara Portolani, Marco Capodiferro, Shilashi Badasa Oljira, Simone Manetto, Alessia Ciogli, Giorgio Bencivenni

**Affiliations:** 1 Department of Industrial Chemistry “Toso Montanari”, Alma Mater Studiorum University of Bologna, Via P. Gobetti 85, 40129 Bologna, Italy; andrea.pellegrini15@unibo.it (A.P.); laura.marcon2@studio.unibo.it (L.M.); giovanni.centonze2@unibo.it (G.C.);; 2 Center for the Chemical Catalysis—C3, Alma Mater Studiorum University of Bologna, Via P. Gobetti 85, 40129 Bologna, Italy; 3 National Interuniversity Consortium of Materials Science and Technology (INSTM), Bologna Research Unit, Via P. Gobetti 85, 40129 Bologna, Italy; 4 Department of Chemistry and Technologies of Drug, Sapienza University of Rome, Piazzale A. Moro 5, 00185 Rome, Italy; shilashibadasa.oljira@uniroma1.it (S.B.O.); simone.manetto@uniroma1.it (S.M.); alessia.ciogli@uniroma1.it (A.C.)

**Keywords:** N-N atropisomers, hydrazides, racemization, dynamic-HPLC

## Abstract

N-N atropisomers represent a useful class of compounds that has recently received important attention from many research groups. This article presents an in-depth analysis of the energy barrier needed for the racemization process of atropoisomeric hydrazides, combining an experimental and computational approach. The focus is on examining how electronic and steric factors impact the racemization process. The results obtained indicate that the barrier observed during the racemization process mainly arises from an increase in the p-orbital character of the nitrogen atoms.

## 1. Introduction

Atropisomers are conformationally stable rotamers that have been recently rediscovered for their important role in medicinal chemistry and chemical biology. Historically, biaryls have been considered representative examples of atropisomerism since they are largely employed as catalysts and ligands for asymmetric synthesis. In recent years, novel types of atropisomers with stereogenic axes different from the C-C single bond have received massive attention from the scientific community. N-N atropisomers signify the latest frontier in atroposelective synthesis; they are valuable for their useful biological activity and their significance as noteworthy “smart” materials [1]. In the past, this class of compounds has been rarely studied by a few research groups that have analyzed the conformational properties of different types of substituted hydrazines, quinazoline-diones, and aminocamphorimides featuring a hindered N-N single bond [2,3,4,5,6,7]. Nevertheless, it is only in the last two years that important examples of the enantioselective preparation of heteroaromatic and hydrazido N-N atropisomers have been reported using consolidated catalytic strategies [8,9,10,11,12,13,14]. In most cases, the high stability of the rotation along the N-N single bond has been determined and ascribed to the steric hindrance of the substituents surrounding the stereogenic axis. When chemists face the synthesis of novel atropisomeric architectures, the experimental determination of the rotational energy barrier gives fundamental information on their stereochemical stability. Most of the time, this value is unknown, and the synthesis can be attempted without a clear indication other than that deriving from the structural comparison with similar compounds previously reported. Also, computational methods, such as Density Functional Theory (DFT), can be complementarily used to study the rotational energy barrier, and in recent years, the use of the aforementioned methods has been employed multiple times, becoming a fundamental topic of scientific reports on atroposelective synthesis. In some cases, they represent the only way to estimate the rotational energy barrier, for example, when the experimental measurement requires high temperatures, which can cause the product to decompose. The robustness of these DFT methods, which are based on the agreement between the computed rotational barrier and the experimental one, makes them a solid tool for the prediction of the rotational energy barrier of plausible atropisomeric compounds. In this way, given a target molecule, it is possible to estimate the energy barrier with a high degree of fidelity and decide whether to attempt the synthesis or not. In this article, the key role that computational calculations have in the prediction of the rotational energy barrier for the racemization of tetrasubstituted hydrazides is reported and subsequently analyzed, clarifying the effects that electronic and steric factors may have in the process. In particular, the value obtained by varying the nature of alkyl substituents surrounding the N-N single bond clearly suggests which are those required to obtain atropisomeric hydrazides and which are not suitable for this purpose. The experimental values obtained using both classical racemization experiments and dynamic approaches were comparable with those computed using DFT methods, thus confirming their usefulness and their crucial role in the design of novel atropisomeric scaffolds. 

## 2. Results and Discussion

**Figure 1:**
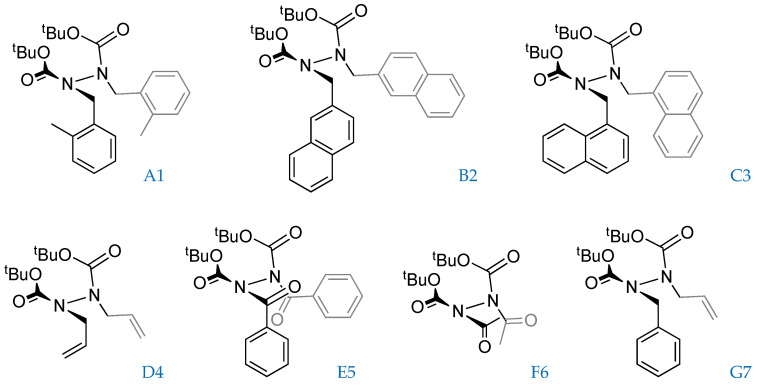
Substrates considered.

At the beginning of our investigation, a series of possible atropisomeric hydrazides was submitted to the computational investigation for the determination of the torsional energy barrier (Figure 1). 

**Figure 2:**
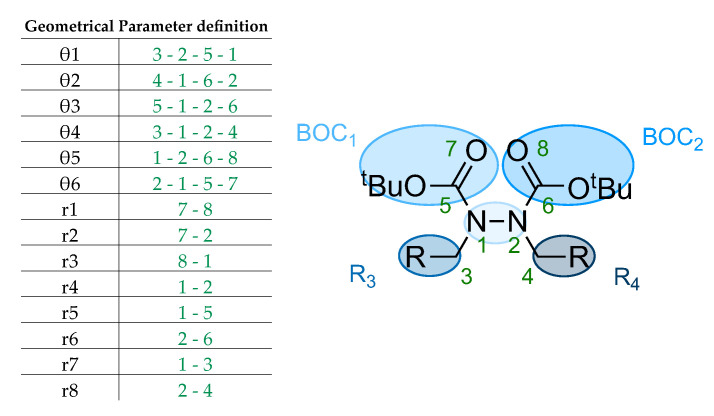
Geometrical parameter definition and fragmentation are used to apply the distortion/interaction model.

To give a description of the geometry, dihedral angles (ϑ), and atomic distances (r) are defined in Figure 2.

### 2.1. Conformational Search

An initial conformation analysis was performed on all seven hydrazides (Figure 1), showing high structural flexibility as evidenced by (i) the high number of conformers obtained in the first conformational search performed with CREST and (ii) the wide dispersion of the Boltzmann populations of the most stable conformers (Table 1).

### 2.2. Ground Potential Analysis

For each molecule, the lower-energy conformer was then optimized at the DFT level. All the optimized structures show nearly perfectly planar sp^2^-hybridized nitrogen atoms, as can be seen from dihedral angles ϑ1 and ϑ2 in Table 2. In addition, the best conformer for all molecules except E5 and G7 has almost perfect C2 symmetry. Three of these structures (B2, D4, and G7) have a ϑ3 dihedral angle around the N-N bond of about 60°, while for the other four structures, this angle is closer to the orthogonal arrangement, varying from 77 to about 88°.

**Figure 3:**
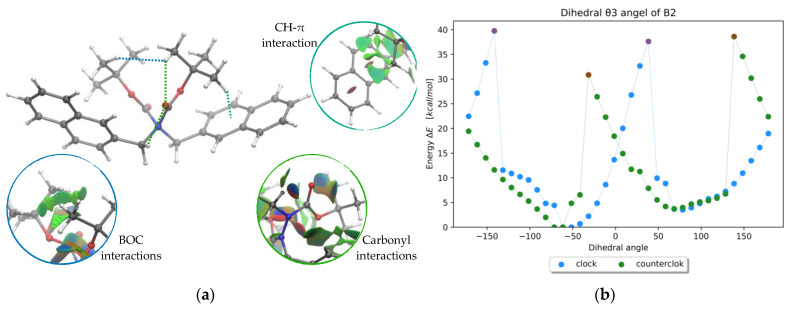
(**a**) B2 best conformer geometry at ωB97x-D/6-31g(d) level and main NCI-index surfaces [17]. (**b**) Scans of the dihedral θ3 angle of the B2 molecule.

These favored conformations appear to be preferred due to two main non-covalent intramolecular interactions (Figure 3a): (a) an interaction between one or two methyl protons of one *t*-butyl group with the π-system of an aromatic substituent on the opposite nitrogen atom, and (b) the interaction between protons of opposite BOC groups. In addition, it is observed that the relative orientation of the carbonyl groups of the BOCs generates an s-*trans* configuration (regarding the NNCO fragment) apart from E5, which presents both carbonyls in an s-*cis* configuration.

Furthermore, an analysis of the total electric dipole moment (Table 3) has been deployed to better understand why conformers prefer the *s*-trans (outside) configuration against the *s*-cis (inside) configuration (Table 3). The opposite conformation had been manually generated by the manual rotation of 180° of the ϑ5 and ϑ6 dihedral angles, followed by an optimization at the same level. It is then possible to observe that they all tend to minimize the dipole moment except for E5, probably thanks to the gain in NCIs between methyl groups and carbonyl oxygen atoms. 

To obtain an initial guess of the geometry of the torsional TS, like common practices [15,16], a relaxed scan of the ϑ3 dihedral angle around the N-N bond was performed (Figure 3b) both in clockwise and counterclockwise modes. The nomenclature adopted refers to GP1 as the previous optimized structure and GP2 as a conformer of its enantiomer obtained from the scan; *cis* refers to TS geometries with the BOC groups on the same side of the σ N-N bond, as opposed to *trans* that have the BOC groups on opposite sides.

### 2.3. Transition State Optimizations

The guess geometries obtained from the scan did not result in geometries particularly close to the actual TSs, greatly increasing the difficulty of the search. Therefore, it became necessary to tweak the maximum displacement parameter for each cycle of optimization in Gaussian. The efficacy of this strategy may be due to a particularly flat PES of these structures, which, in the search for a transition state, can easily lead to loss of the correct frequency at an excessive motion. This workflow resulted in finding most of the correct TS structures except those for E5 and F6. For these elusive TSs, new predicted geometries were obtained using the Growing String Method [14] (GSM) coupled with xTB. This approach allowed us to obtain TS geometries at the DFT level for all remaining structures. Geometrical parameters for the so-found TSs are reported in Table 4.

**Figure 4:**
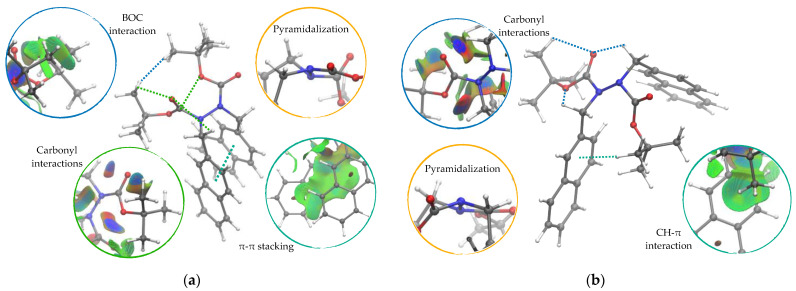
(**a**) B2-Cis TS at ωB97x-D/6-31g(d) level obtained from the counterclockwise relaxed scan of the dihedral angle around the N-N bond. (**b**) B2-Trans TS at ωB97x-D/6-31g(d) level obtained from the clockwise relaxed scan of the same dihedral [17].

A common feature was noted in the spacing of the carbonyl oxygen atoms of 4–5 Å, except for some cis TS geometries. Similarly, there are N-N bond distances around 1.4 Å, slightly longer than the bonds in the minimal structures. Both nitrogen atoms also show strong opposite pyramidalization, as displayed by dihedral angles ϑ1 and ϑ2 (Table 4 and orange circle in Figure 4). Compared with equilibrium structures, TS geometries exhibit a mixed s-*cis* and s-*trans* configuration for BOC fragments, as shown by ϑ5 and ϑ6.

From NCI surface analysis (Figure 4 and Supplementary Materials), some important interactions can be detected: (i) π-π stacking between aromatic systems; (ii) CH-π interaction of the methyl groups pointing towards an aromatic ring; (iii) a carbonyl interaction of the oxygen atoms with the hydrogen of the opposite CH_2_; (iv) a BOC interaction between the carbonyl oxygen of the BOCs that can also give hydrogen bonds on its own *t*-butyl.

The HOMO orbital of all the TSs is mainly localized in the π-system (when present), as was in the GPs analyzed. In contrast, it is possible to observe a decrease in carbamate fragment conjugation (see Supplementary Materials for more information).

### 2.4. Energy Barriers from Experimental Data

The atropisomeric interconversion of structurally symmetric samples (A1–F6) was investigated by liquid chromatography. (In the case of compound G7, decomposition was observed during the experimental determination of the rotational barrier).

Two approaches were used: (i) the off-column racemization process monitored by enantioselective HPLC and (ii) the on-line enantiomerization process on chiral stationary phase at variable temperature. After initial screening, aiming for the optimization of analytical conditions for each racemate, racemization and/or enantiomerization experiments were achieved. As reported in Table S1, samples F6 and E5 were baseline separated at low temperatures (10 °C and 0 °C, respectively), attesting to the fast-interconverting speed of each atropisomeric pair. In fact, when analyzed at room temperature, they showed a typical plateau between two peaks. Chromatographic UV and CD profiles were reported in Figures S1 and S2.

Energy values of enantiomerization processes, temperature, and speed constants are listed in Table 5. Notably, the ΔG of racemization (ΔG_rac_) comes from the off-column experiment, while the ΔG of enantiomerization (ΔG_enant_) comes from the on-column one. The relationship between two values is: *k racemization* = 2*k enantiomerization*. To make the comparison easier with computational data, all racemization-free energies were converted into the corresponding enantiomerization values.

**Figure 5:**
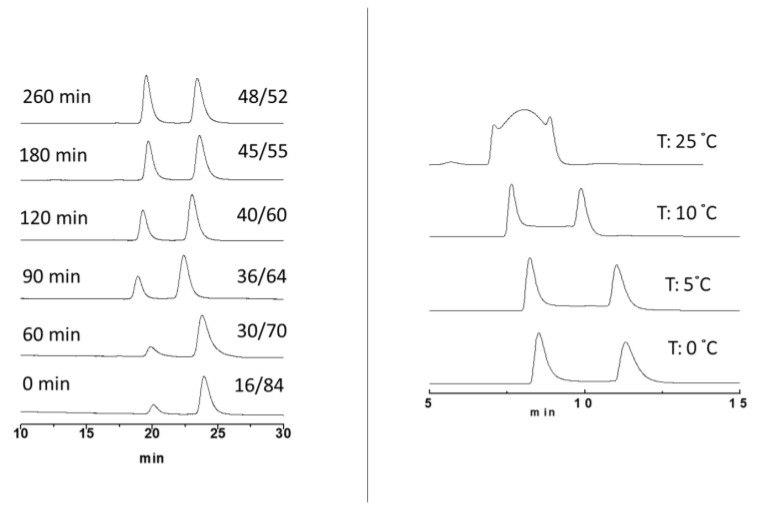
Thermal racemization of B2 (**left**) and enantiomerization of E5 (**right**).

For A1 to D4 samples, an off-column approach was employed at 70 degrees, and the values of the higher free activation energy were expressed by sample C3 (ΔG_enant_ 26.01 kcal/mol). For sample A1, the value of ΔG_enant_ < 24.3 kcal/mol is an estimated value due to the fast racemization (complete in less than 30 min) and to the low column performance in terms of enantioselectivity (α = 1.12). A more flexible N-N bond is the one in sample F6. The plateau is clearly visible at 40 °C (see chromatogram in Figure S3), and by Auto DHPLC y2k, the simulated chromatogram provided a ΔG_enant_ of 23.10 kcal/mol. Unfortunately, it was not possible to acquire chromatograms at higher temperatures to preserve the integrity of the polysaccharide-based stationary phase. A lower energy value has been recorded from sample E5. The ΔG_enant_ of 20.61 kcal/mol at 25 °C has been calculated by simulation of the corresponding dynamic chromatogram (chromatographic trace on the top of Figure 5 right).

### 2.5. Energy Barrier Values at Different Temperatures for Samples 3 and 6: Eyring Plots

Exploring the dependence of free energy versus temperature, two Eyring plots were built from samples B2 and E5 (Table S2 and Figure S4). Data were obtained by off-column racemization experiments for sample B2 and by on-column enantiomerization experiments for sample E5. In both cases, a slight increment of ΔG was recorded as a temperature increase. In addition, good linearity was observed when plotting 1/T vs. ΔG/T (R^2^ = 0.9936 and R^2^ = 0.9979 for samples B2 and E5, respectively), and the corresponding equations have shown a not negligible entropic effect in the N-N interconversion process. The ΔH values are 15.45 kcal/mol and 17.01 kcal/mol, while the ΔS values are −27.9 u.e. and −12.0 u.e., respectively, for sample B2 and sample E5. Figure 5 reports as an example the off-column and on-column approaches employed in this study.

### 2.6. Comparison Analysis

After these analyses, the obtained energy barrier values have been compared with the experimental ones (Table 6). To better compare the two data, the mRRHO approximation [18] were computed at the temperature of the experiment. It has been noticed that trans-TS is the one determining the epimerization barrier due to its lower energy for all compounds analyzed.

**Figure 6:**
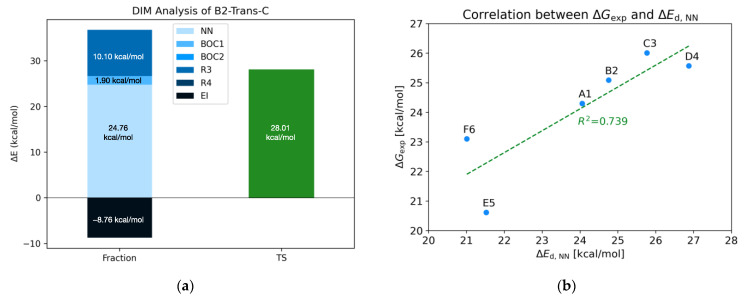
(**a**) DIM analysis of the trans TS for B2 molecule at ωB97x-D/6-31g(d) level. (**b**) Correlation between experimental ∆G and NN distortion energy.

To obtain as much information as possible on the nature of the rotation barrier, further analyses were conducted, such as the Distortion/Interaction Model [19,20] (DIM) and Natural Bond Order [21,22] (NBO). The employment of the DIM allowed an energy estimate to be attributed to the contribution of the various fragments into which the molecule was divided (Figure 2) to the energy of the TS. The plot of Figure 6a, each fragment distortion energy (Equation (1)) and interaction energy value (Equation (2)) have been stacked (on the left) to compare them with the TS energy (on the right). In many of the TSs analyzed, the distortion energy of the fragments is destabilizing; only rarely is this value mildly stabilizing. This phenomenon can be rooted back to internal fragment tension release, thanks to the more room the fragment is given in such an open TS as these ones.

The largest contribution to the TS energy is the deformation of the N-N fragment, while the interaction energies can be stabilizing or destabilizing (Figure 6a and others in Supplementary Materials).

To have a better understanding of the actual nature of the barrier [23], a correlation between the experimental ∆G and the calculated distortion energy that seems to characterize the ∆G^‡^ (Figure 6b) has been reported. A fair correlation came out: the not-so-high R^2^ correlation parameter is, for the most part, due to points F6 and E5, for which their ∆G_exp_ were obtained at different temperatures than the others.

The correlation found indicates that the barrier observed during the racemization process is not only due to the torsion about the σ N-N bond and the clash of the substituents generating the entire rotational barrier, but also to an increase in the *p*-orbital character of the nitrogen atoms. This hypothesis is also supported by the loss of conjugation of the nitrogen atoms with the carbonyl system of the BOC and their pyramidalization, as it is notable from the increase of the r5, r6, r7, and r8 distances and from the surfaces of the HOMO orbitals.

To further confirm this assumption, a NBO population analysis was carried out. This study allowed us to define the percentage of *p* orbitals present in the bonds in which nitrogen atoms are involved. In Table 7, the differences in the percentage of the *p* orbitals of the nitrogen atom between TSs and GPs are correlated to the difference in the bond distance. As previously supposed, slightly more *p* characters are present in TSs.

In addition to these parameters, bond angles were measured for each nitrogen in the molecules. This, along with ϑ1 and ϑ2, facilitated the assessment of nitrogen pyramidalization and deviation from perfect sp^2^ hybridization, indicating proximity to sp^3^ characteristics.

Theoretical expectations dictate that the sum of bond angles around a perfectly sp^2^ nitrogen should be 360°, while for a perfectly sp^3^ nitrogen (taking ammonia as a reference), it is 321° (three times the H–N–H bond angle of 107°).

To quantify this pyramidalization of the nitrogen atoms in the TSs geometries, for each one of the two nitrogen atoms, the difference between the sum of bond angles in the GP and the sum of bond angles in the TS geometry was calculated. These values are reported in Table 8 in absolute terms as ∆A and as the %ratio (∆A%) between ∆A and the difference between perfectly sp^2^ and sp^3^ observed for ammonia (39°, as shown in Table 8-top) taken as a reference.

**Figure 7:**
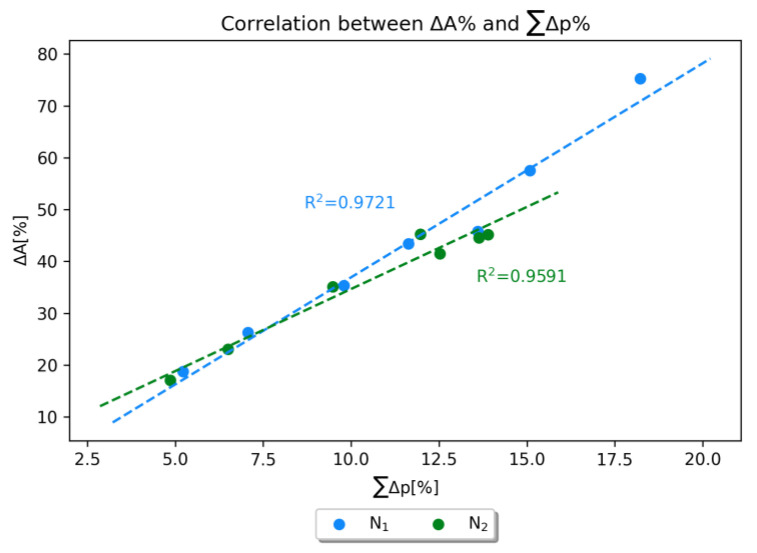
Correlation between the %ratio of the bond angles and the overall sum of the *p* character of the nitrogen atoms.

In summary, the consideration of the nitrogen atom pyramidalization involves two key parameters: the sum of the bond angles of the substituents on each nitrogen atom (∆A%) and the overall increase in the *p* character in the bonds around the nitrogen atoms. It is noteworthy that all geometries exhibit a high ∆A%, and each bond demonstrates a higher *p* percentile in the TS rather than in the GP. This observation is supported by the good correlation between these two factors: the more the sum of the *p* orbital into the bond, the more the sum of the bond angles approaches the ideal sp^3^ geometry (Figure 7).

## 3. Methods and Materials

### 3.1. Computational Methods

For each substrate, an initial conformational search was performed using the CREST [24] software utility (version 2.11.1), which is based on the xTB [25] engine (version 6.4.1), and it executes an interactive meta-dynamics with a cross-genetic grow algorithm (called iMTD-GC). This analysis was conducted at 298.15 K at the GFN2-xTB [26] level, discarding all conformers with an energy higher than the predefined cutoff value of 6 kcal/mol relative to the lowest-energy conformer.

The seven conformer ensembles obtained were then refined at the DFT-theory level using the CENSO [27] (version 1.2.0) framework interfaced with the ORCA quantum chemistry program package (version 5.0.1) [28] according to the following protocol:

1. part0 *cheap prescreening*: b97-d3 [29]/def2-SV(P)//GFN2-xTB (Input geometry)
2. part1 *prescreening*: r^2^scan-3c [30] + C-PCM[hexane] + GmRRHO [18] (GFN2[ALPB [31]]-bhess [32])//GFN2-xTB (Input geometry)
3. part2 *optimization*: r^2^scan-3c + C-PCM[hexane] + GmRRHO(GFN2[ALPB]-bhess)//r^2^scan-3c[SMD]

This framework enabled faster processing of the vast conformational ensemble of a large number of conformers since each part analyzed the conformers and sorted out those with a relative energy greater than the default (for *part0* 4.0 kcal/mol, *part1* 3.5 kcal/mol, and *part2* 2.5 kcal/mol). For the last two parts, the free energy was calculated via the mRRHO approximation [18].

All following calculations have been performed using the Gaussian16 (rev. A.03) [33] program at the DFT level with the ωB97x-D/6-31g(d) model chemistry and C-PCM implicit solvation in *n*-hexane. These parameters were chosen on the basis of previous works on similar compounds [34]. Free energy has been calculated with a vertical excitation energy correction, modifying the functional and the basis set to M06-2X/def2-TZVP, and using the previously calculated thermochemical correction [35].

To accurately describe non-covalent interactions, the NCI index was calculated with the NCIPLOT [36] software (version 4.0) using the FINE grid of integration.

To study the energetic contributions to the transition states (TS), the distortion-interaction model (DIM) was employed [19,20]. This analysis was carried out by fragmenting each molecule into five groups (Figure 2): each of four substituents and the N-N core. For each fragment, the distortion energy ($E_{d}^{i}$) was calculated as the difference between the energy of the segment at its TS geometry ($E_{TS_{i}}^{*}$) and at its ground potential (GP1) geometry ($E_{GP_{i}}$). Thus, the interaction energy ($E_{i}$) was obtained by the difference between the single point energy of the whole TS and the sum of all distortion energies.

(1) $$E_{d}^{i}=E_{TS_{i}}^{*}-E_{GP_{i}}$$

(2) $$E_{i}=E_{TS}-\sum^{\mathrm{fragment}}E_{d}^{i}$$

### 3.2. Synthesis and Experimental Part

**Figure 8:**
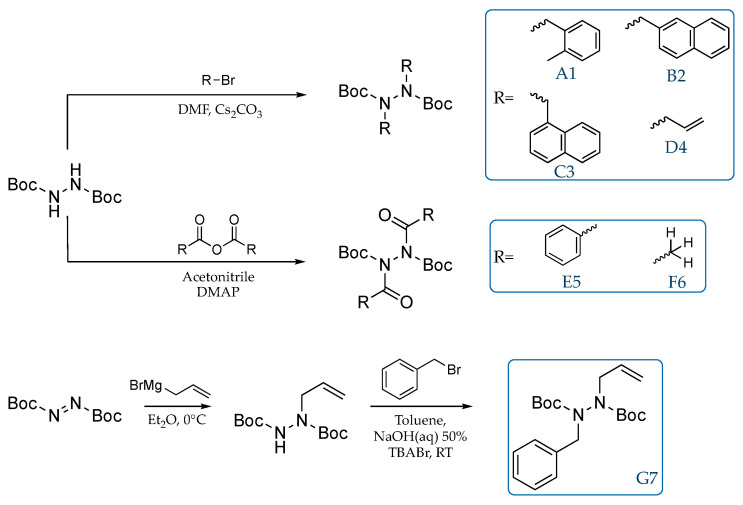
Synthetic procedures for investigated molecules.

Hydrazides have been synthesized with an achiral methodology, as reported in Figure 8. The synthetic strategy can be divided into three categories: the first two employed the di-*tert*-butyl hydrazine-1,2-dicarboxylate and the corresponding bromide derivates or anhydrides to give A1-F6 compounds. In alternative, di-*tert*-butyl azodicarboxylate has been employed in a sequential pathway to obtain compound G7. Only the first one has already been discussed in the literature [37]; the others were not optimized procedures.

## 4. Conclusions

During this study, a systematic computational and experimental analysis of seven atropoisomeric hydrazides was conducted. For the herein studied molecules, both the GPs and the transition state geometries have been thoroughly investigated, in good accordance with the experimental racemization studies. Furthermore, with both the DIM and the NBO analyses, it has been possible to assign as the cause of the rotational impeding the predominant distortion energy of the N–N bonds and the partial rehybridization of the two nitrogen atoms.

## Figures and Tables

**Table 1:** Number of conformers generated from the CREST-CENSO protocol. Values in parentheses represent the Boltzmann population of the best conformer at that stage.

Parameter	CREST	*Part0*	*Part1*	*Part2*
A1	93 (67.0%)	89	78	48 (31.5%)
B2	241 (22.2%)	177	127	96 (4.5%)
C3	125 (46.9%)	72	37	31 (29.4%)
D4	71 (24.7%)	47	37	30 (17.0%)
E5	86 (30.1%)	60	59	51 (11.2%)
F6	66 (9.8%)	40	40	35 (7.5%)
G7	115 (38.1%)	83	62	50 (13.1%)

**Table 2:** Geometrical parameters of the best conformer for each compound at ωB97x-D/6-31g(d) level.

Parameter	A1	B2	C3	D4	E5	F6	G7
ϑ1 [°]	−0.75	9.26	−1.26	−10.41	4.35	1.48	−8.31
ϑ2 [°]	−0.75	8.84	−1.25	−10.41	−5.82	1.48	−11.52
ϑ3 [°]	77.89	−61.55	77.58	62.73	87.70	−84.12	62.11
ϑ5 [°]	179.87	171.67	179.23	−171.38	19.01	179.24	−170.04
ϑ6 [°]	179.87	171.35	179.23	−171.38	−20.29	179.24	−172.92
r4 [Å]	1.37	1.38	1.37	1.38	1.37	1.38	1.38
r5 [Å]	1.38	1.38	1.38	1.38	1.41	1.41	1.38
r6 [Å]	1.38	1.38	1.38	1.38	1.41	1.41	1.38

**Table 3:** Dipole momentum of the optimized structures at ωB97x-D/6-31g(d) level.

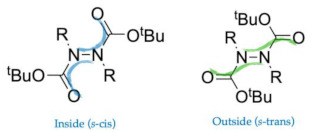			
	Inside	Outside	ΔG kcal/mol (In-Out)
A1	1.9848 ^[b]^	0.9121 ^[a]^	+5.85
B2	1.5692 ^[b]^	0.8129 ^[a]^	+8.21
C3	1.9374 ^[b]^	0.8278 ^[a]^	+8.47
D4	1.5456 ^[b]^	0.8058 ^[a]^	+3.32
E5	2.1177 ^[a]^	5.2156 ^[b]^	−1.75
F6	2.7641 ^[b]^	5.7090 ^[a]^	+3.92
G7	1.7223 ^[b]^	0.6977 ^[a]^	+5.43

^[a]^ structures generated from the CREST-CENSO protocol. ^[b]^ manually generated by manually rotating by 180° the ϑ4 and ϑ5 dihedral angles of the structures.

**Table 4:** Geometrical parameters of the transition state determine the torsional barrier at the ωB97x-D/6-31g(d) level.

Parameter	A1	B2	C3	D4	E5	F6	G7
ϑ1 [°]	20.71	−21.33	35.49	23.46	−25.12	−31.56	−31.60
ϑ2 [°]	−29.53	28.85	−20.30	−34.37	28.25	24.53	19.85
ϑ3 [°]	−173.62	−161.50	170.61	−177.37	169.33	−166.89	−176.93
ϑ5 [°]	162.93	−166.00	−179.60	11.77	−129.29	−179.69	−2.07
ϑ6 [°]	−1.71	13.97	39.47	−172.14	−40.38	−8.82	169.36
r4 [Å]	1.44	1.44	1.44	1.44	1.42	1.43	1.44
r5 [Å]	1.39	1.38	1.44	1.40	1.46	1.41	1.41
r6 [Å]	1.41	1.42	1.39	1.40	1.47	1.40	1.39

**Table 5:** Energy values of enantiomerization processes, temperature, speed constants, and half-life for each sample were listed.

Sample	T [°C]	k_rac_ [min^−1^]	k_enant_ [min^−1^]	ΔG_enant_ [kcal/mol]	t_1/2_ [min]
D4	70	0.0111 (R^2^ = 0.9974)	-	25.57	31
C3	71	0.0064 (R^2^ = 0.9989)	-	26.01	54
B2	71	0.0250 (R^2^ = 0.9991)	-	25.09	14
A1	70	0.0645	-	<24.3 ^a^	5.4
F6	40	-	0.0296	23.10 ^b^	12
E5	25	-	0.3560 ^b^	20.61 ^b^	2

^a^ estimated value: the racemization is completed in 30 min. ^b^ averaged value between the free energy of the direct (ΔG_1-2_) and reversed (ΔG_2-1_) enantiomerization processes.

**Table 6:** Single-point refined rotational ΔG at M06-2x/def2-TZVP//ωB97x-D/6-31g(d) level.

Molecule	∆G Exp [kcal/mol]	T exp [°C]	∆G Computational [kcal/mol]	Deviation from Experimental Value [%]
A1	<24.3	70	29.23	20.29 ^[a]^
B2	25.09	71	26.31	4.87
C3	26.01	71	36.23	39.29
D4	25.57	70	24.81	−2.96
E5	20.61	25	22.57	9.52
F6	23.10	40	28.46	23.20
G7	N.A.		26.87 ^[b]^	N.A.

N.A.: not available. ^[a]^: considering experimental ΔG = 24.3 kcal/mol; ^[b]^: calculated at 298.15 K.

**Table 7:** NBO analysis at ωB97x-D/6-31g(d) level.

Bond ^[a]^	A1		B2		C3		D4		E5		F6		G7	
	∆bd [Å]	∆*p* [%]	∆bd [Å]	∆*p* [%]	∆bd [Å]	∆*p* [%]	∆bd [Å]	∆*p* [%]	∆bd [Å]	∆*p* [%]	∆bd [Å]	∆*p* [%]	∆bd [Å]	∆*p* [%]
1–2	0.07	2.56	0.06	2.10	0.07	4.88	0.06	3.55	0.05	3.02	0.05	2.96	0.06	3.98
2–1	0.07	3.90	0.06	4.34	0.07	2.53	0.06	3.39	0.05	2.84	0.05	2.41	0.06	2.26
1–3	0.01	1.56	0.01	2.02	0.03	5.81	0.03	3.16	−0.01	2.01	0.07	7.23	0.04	5.18
1–5	0.01	2.95	0.00	1.10	0.06	7.53	0.02	3.09	0.05	6.60	0.00	4.90	0.04	4.44
2–4	0.04	5.20	0.03	3.64	0.01	1.33	0.02	2.85	−0.01	3.40	0.06	7.03	0.02	0.85
2–6	0.04	4.79	0.04	5.65	0.01	2.64	0.02	3.24	0.06	5.73	0.00	3.09	0.00	1.75

∆bd: difference in bond distance between TS and GP. ∆*p* [%]: difference in %*p* orbital involved in the bond (%*p*_TS_-%*p*_GS_). ^[a]^: The numbers defining the bond refer to the indexing encoded in Figure 2.

**Table 8:** Difference of angles (GP-TS). The index of the nitrogen is according to Figure 2 numeration.

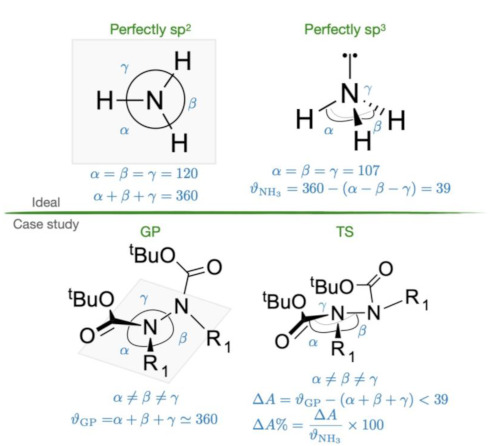				
	N_1_		N_2_	
Molecule	∆A	∆A [%]	∆A	∆A [%]
A1	10.26	26.31	10.26	26.31
B2	7.33	18.79	17.38	44.56
C3	29.34	75.23	9.00	23.08
D4	13.80	35.38	13.70	35.13
E5	16.93	43.41	17.66	45.28
F6	22.44	57.54	16.19	41.51
G7	17.85	45.77	6.68	17.13

## Data Availability

Data are contained within the article and Supplementary Materials.

## Supplementary Materials

The following supporting information can be downloaded at: https://www.mdpi.com/article/10.3390/molecules28237856/s1; File S1 Supporting information, refs [38,39,40,41,42,43,44,45] are cited in Supplementary Materials.
