# Supplementary material for: On the Nature of the Rotational Energy Barrier of Atropisomeric Hydrazides
Source: Molecules. 2023 Nov 29;28(23):7856. doi: 10.3390/molecules28237856 (PMC10708319; doi:10.3390/molecules28237856)
Supplement: Supplementary file 1 [file molecules-28-07856-s001.zip › molecules-2708809-supplementary.pdf]

# On the Nature of the Rotational Energy Barrier of Atropisomeric Hydrazides

*Andrea Pellegrini* [1,2], *Laura Marcon* [1], *Paolo Righi* [1,2,3], *Giovanni Centonze* [1,2], *Chiara Portolani* [1,2], *Marco Capodiferro* [1], *Shilashi Basasa Oljira* [4], *Simone Manetto* [4], *Alessia Ciogli* [4] *and Giorgio Bencivenni* [1,2,3,*]

[1]Department of Industrial Chemistry "Toso Montanari"- Alma Mater Studiorum University of Bologna, via P. Gobetti 85, 40129 Bologna, Italy

[2]Center for the Chemical Catalysis—C[3], Alma Mater Studiorum University of Bologna, via P. Gobetti 85, 40129 Bologna, Italy

[3]National Interuniversity Consortium of Materials Science and Technology (INSTM)—Bologna Research Unit—via P. Gobetti 85, 40129 Bologna, Italy

[4]Department of Chemistry and Technologies of Drug, Sapienza University of Rome, piazzale A. Moro 5, 00185 Rome, Italy

*Correspondence: giorgio.bencivenni2@unibo.it

**Supporting information**

***Synthesis of tetrasubstituted hydrazides*** ____ **2**

**Alkylation strategy[1]** ____ 2

**Acylation strategy** ____ 3

**Consecutive alkylation strategy** ____ 4

***Materials for HPLC method development*** ____ **6**

***Computational study*** ____ **11**

**General information** ____ 11

**Conformational searches and refinement of the ensembles** ____ 11

**TS search for the N—N axial epimerization** ____ 18

**DIM Analysis** ____ 33

***Geometries*** ____ **39**

## General

All the NMR spectra were recorded on Gemini 400 MHz or Mercury 600 MHz Varian spectrometers for $^1$H, 101 MHz for $^{13}$C. The chemical shifts (δ) for $^1$H, $^{13}$C are given in ppm relative to internal standard TMS (0.0 ppm) or residual signals of $CHCl_3$ (7.26 ppm). The following abbreviations are used to indicate the multiplicity: s, singlet; d, doublet; t, triplet; q, quartet; m, multiplet; bs, broad signal. Purification of reaction products was carried out by flash chromatography on silica gel (230-400 mesh). Organic solutions were concentrated under reduced pressure on a Büchi rotary evaporator. All reagents used are commercially available and purchased from suppliers. Procedures using bromide derivates have been taken from literature[1] and $^1$HNMR of the products were consistent with those previously reported. Procedures using anhydrides and the two-step synthesis have not been optimized.

### *Synthesis of tetrasubstituted hydrazides*

*Alkylation strategy[1]*

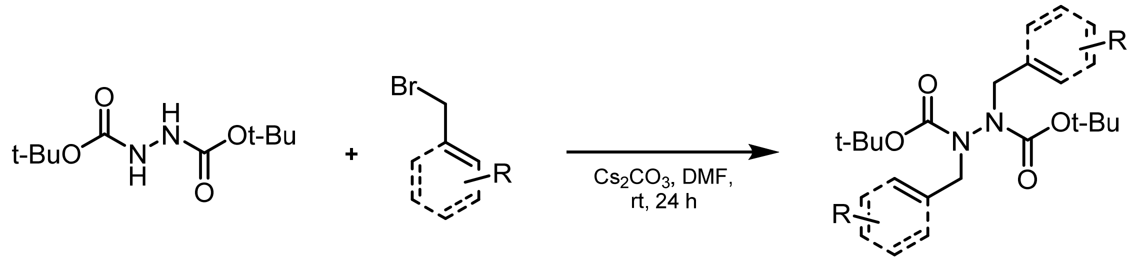


*General procedure:*

To a suspension of $Cs_2CO_3$ (3 eq) in DMF [0.6] were added di-*tert*-butyl hydrazine-1,2-dicarboxylate (1 eq) and the corresponding benzyl bromide (3 eq); only in the case of the synthesis of the compound **D4** 4 eq of allyl bromide were required. The reaction was quenched after 24 hours with distilled water and the mixture was extracted three times with ethyl acetate. The organic phases were collected, washed with brine, dried on $Na_2SO_4$ and finally concentrated under reduced pression. Products **A1**, **B2**, **D4** didn't required further purifications; the compound **C3** was isolated after a flash-chromatography (mixture: 1:6 $Et_2O$/*n*-hexane).

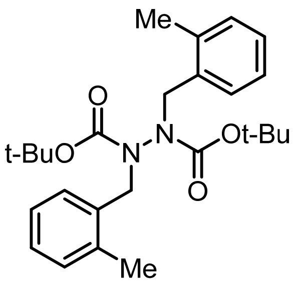


**A1** yield: 72%

**Melting point**: 74°C

**[M+H]$^+$** found = 441.2746 m/z; calculated = 441.2748 m/z

**$^1$H NMR** (400 Hz, $CDCl_3$) δ 7.23 - 6.87 (m, 8H), 4.87 - 3.87 (m, 4H), 2.40 - 2.12 (m, 6H), 1.55 - 1.11 (m, 18H).

**$^{13}$C NMR** (101 MHz, $CDCl_3$) δ 154.73, 137.70, 134.50, 131.29, 130.24, 127.94, 125.78, 125.51, 80.69, 48.57, 28.18, 27.86, 19.73.

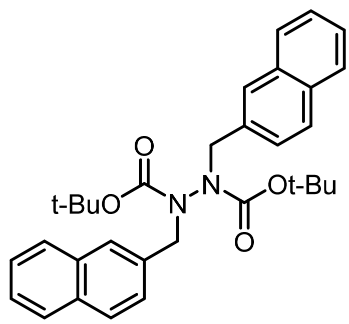

**B2** yield: 71%

**Melting point**: 87°C

**[M+H]⁺** found = 513.2754 m/z; calculated = 513.2748 m/z

**$^{1}$H NMR** (400 Hz, $CDCl_3$) δ 7.87 - 7.10 (m, 14H), 5.38 - 4.29 (m, 4H), 1.50 - 1.24 (m, 18H).

**$^{13}$C NMR** (151 MHz, $CDCl_3$) δ 192.27, 160.83, 156.33, 155.45, 155.18, 154.61, 136.49, 135.03, 134.84, 134.61, 134.58, 134.38, 134.16, 133.29, 133.23, 133.20, 133.16, 133.05, 133.00, 132.82, 132.78, 132.68, 132.64, 132.62, 132.58, 132.55, 129.59, 129.56, 129.13, 128.54, 128.49, 128.40, 128.31, 128.10, 128.05, 127.95, 127.92, 127.79, 127.75, 127.73, 127.71, 127.68, 127.62, 127.60, 127.57, 127.50, 127.37, 127.28, 127.19, 127.11, 127.06, 126.93, 126.60, 126.47, 126.45, 126.42, 126.36, 125.97, 125.87, 125.84, 125.81, 125.68, 125.55, 123.61, 122.80, 81.87, 81.61, 81.20, 81.13, 81.08, 80.94, 69.94, 65.85, 56.02, 55.83, 54.14, 53.30, 51.49, 46.51, 29.76, 28.36, 28.08, 15.33, 14.19.

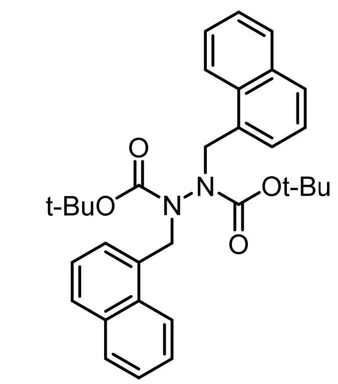

**C3** yield: 43%

**Melting point**: 165°C

**[M+H]⁺** found = 513.2753 m/z; calculated = 513.2748 m/z

**$^{1}$H NMR** (400 MHz, $CDCl_3$) δ 8.34 – 8.22 (m, 2H), 7.83 (m, 2H), 7.79 – 7.69 (m, 2H), 7.65 – 7.48 (m, 2H), 7.52 – 7.44 (m, 2H), 7.42 – 7.26 (m, 2H), 7.15 – 7.02 (m, 2H), 5.07-5.00 (m, 2H), 4.63 – 4.48 (m, 2H), 0.97-0.88 (m, 18H).

**$^{13}$C NMR** (101 MHz, $CDCl_3$) δ 154.58, 133.68, 132.64, 132.44, 129.23, 128.91, 128.69, 128.65, 128.42, 126.34, 125.74, 125.13, 125.02, 80.58, 48.61, 28.41, 27.57, 27.34.

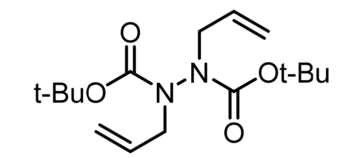

**D4** yield: 63%.

**$^{1}$H NMR** (400 Hz, $CDCl_3$) δ 5.97 - 5.82 (m, 2H), 5.24 - 5.06 (m, 4H), 4.07 - 3.81 (m, 4H), 1.51 - 1.39 (m, 18H).

**$^{13}$C NMR** (101 MHz, $CDCl_3$) δ 155.38, 154.69, 154.29, 134.01, 133.91, 133.49, 118.08, 117.81, 117.23, 81.04, 80.91, 80.83, 54.75, 52.60, 52.56, 28.22, 28.15.

*Acylation strategy*

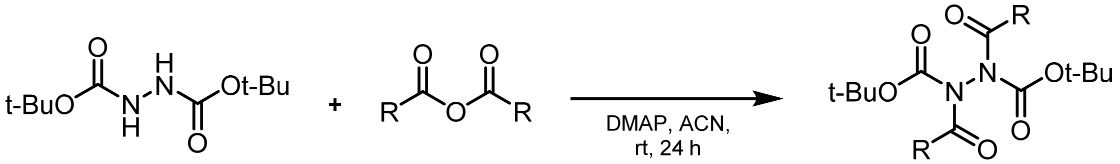


*General procedure:*

To a solution of di-*tert*-butyl hydrazine-1,2-dicarboxylate (1 eq) and 4-dimethylamino-pyridine (DMAP)(1 eq) in acetonitrile [0.5], was added the corresponding anhydride (6 eq). After the consumption of the starting hydrazide monitored by TLC, the reaction was diluted with $Et_2O$ and extracted firstly with 0.1 N HCl solution, then with 1 M KOH solution. The organic phase was eventually washed with the brine solution, then dried on $Na_2SO_4$ and concentrated under reduced pressure. The products didn't require further purifications.

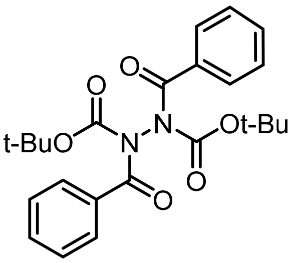


**E5** yield: 54%

**Melting point**: 105°C

**[M+H]$^+$** found = 441.2020 m/z; calculated = 441.2014 m/z

**$^1$H NMR** (400 Hz, $CDCl_3$) δ 7.78-7.73 (m, 4H), 7.57-7.51 (m, 2H), 7.47-7.41 (m, 4H), 1.28(s, 18H).

**$^{13}$C NMR** (101 MHz, $CDCl_3$) δ 169.70, 150.67, 135.39, 133.73, 131.82, 130.18, 128.48, 128.22, 128.11, 84.84, 27.42.

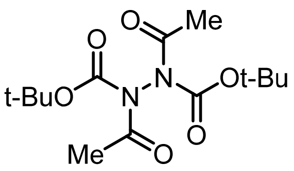


**F6** yield: 77%

**[M+H]$^+$** found = 317.1707 m/z; calculated = 317.1704 m/z

**$^1$H NMR** (400 Hz, $CDCl_3$) δ 2.54 (s, 6H), 1.49 (s, 18H).

**$^{13}$C NMR** (151 MHz, $CDCl_3$) δ 206.96, 170.92, 168.71, 155.82, 154.30, 151.59, 150.26, 84.55, 84.43, 84.29, 84.17, 81.77, 81.28, 65.78, 30.84, 28.12, 28.07, 27.81, 27.73, 25.31, 25.05, 15.19

*Consecutive alkylation strategy*

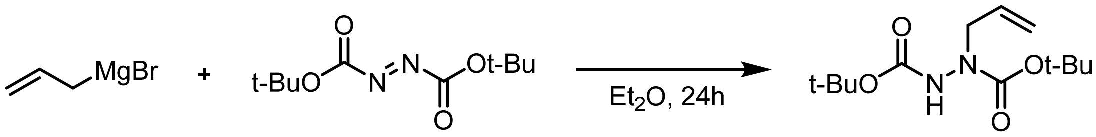


To a mixture of di-*tert*-butyl diazene-1,2-dicarboxylate (1 eq) in anhydrous $Et_2O$ [0.5], allylmagnesium bromide (1 M in $Et_2O$, 2 eq) was added dropwise at 0°C. After 24 hours, the solution was quenched with a saturated ammonium chloride solution and then extracted with dichloromethane (DCM). The crude was purified with a flash chromatography (2:1 DCM/*n*-hexane).

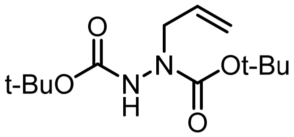


Yield: 42%

HNMR and CNMR were identical to those reported in the literature[2].

Afterwards, the isolated intermediate was subsequently converted into the compound **G7** performing a phase transfer strategy. Therefore, 1 eq of the trisubstituted hydrazide was dissolved in toluene [0.05] to which was added NaOH 50%w/w (2 times the volume of toluene), tetrabutylammonium bromide (1 eq) and benzyl bromide (1 eq). After one day, the reaction was stopped separating the 2 phases, then extracting the water phase 2 times with $Et_2O$. The flash chromatography (1:3 $Et_2O$:*n*-hexane) allowed the isolation of **G7** with a total yield of 58%.

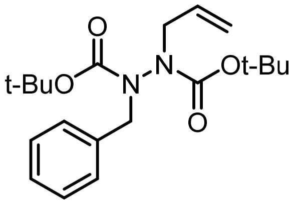


**G7**: yield: 58%

**$[M+H]^+$** found = 363.2278 m/z; calculated = 363.2285 m/z

**$^1$H NMR** (400 Hz, $CDCl_3$) δ 8.18 - 6.92 (m, 5H), 5.85 - 3.46 (m, 7H), 1.62 - 1.16 (m, 18H).

**$^{13}$C NMR** (101 MHz, $CDCl_3$) δ 156.02, 155.31, 155.02, 154.90, 154.61, 154.40, 154.21, 137.21, 137.18, 136.77, 136.68, 133.75, 133.30, 129.77, 129.75, 129.40, 129.10, 128.94, 128.30, 128.27, 128.25, 128.21, 128.02, 127.61, 127.50, 127.49, 127.44, 117.91, 117.57, 116.83, 81.29, 81.09, 81.01, 80.98, 80.80, 80.75, 55.21, 54.94, 53.18, 52.82, 52.65, 52.63, 52.51, 28.24, 28.17, 28.06, 28.01, 27.97.

*NMR traces*

**¹H NMR** (400 Hz, $CDCl_3$)

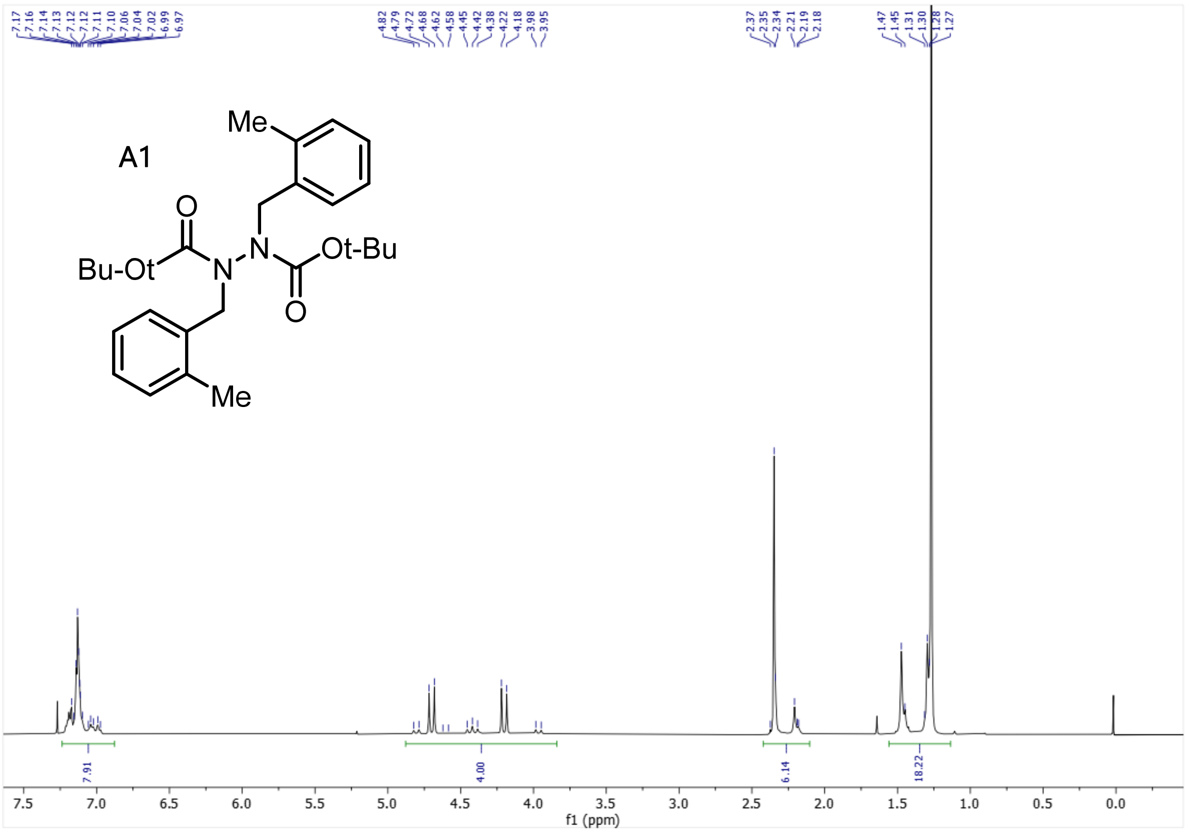


**¹³C NMR** (101 MHz, $CDCl_3$)

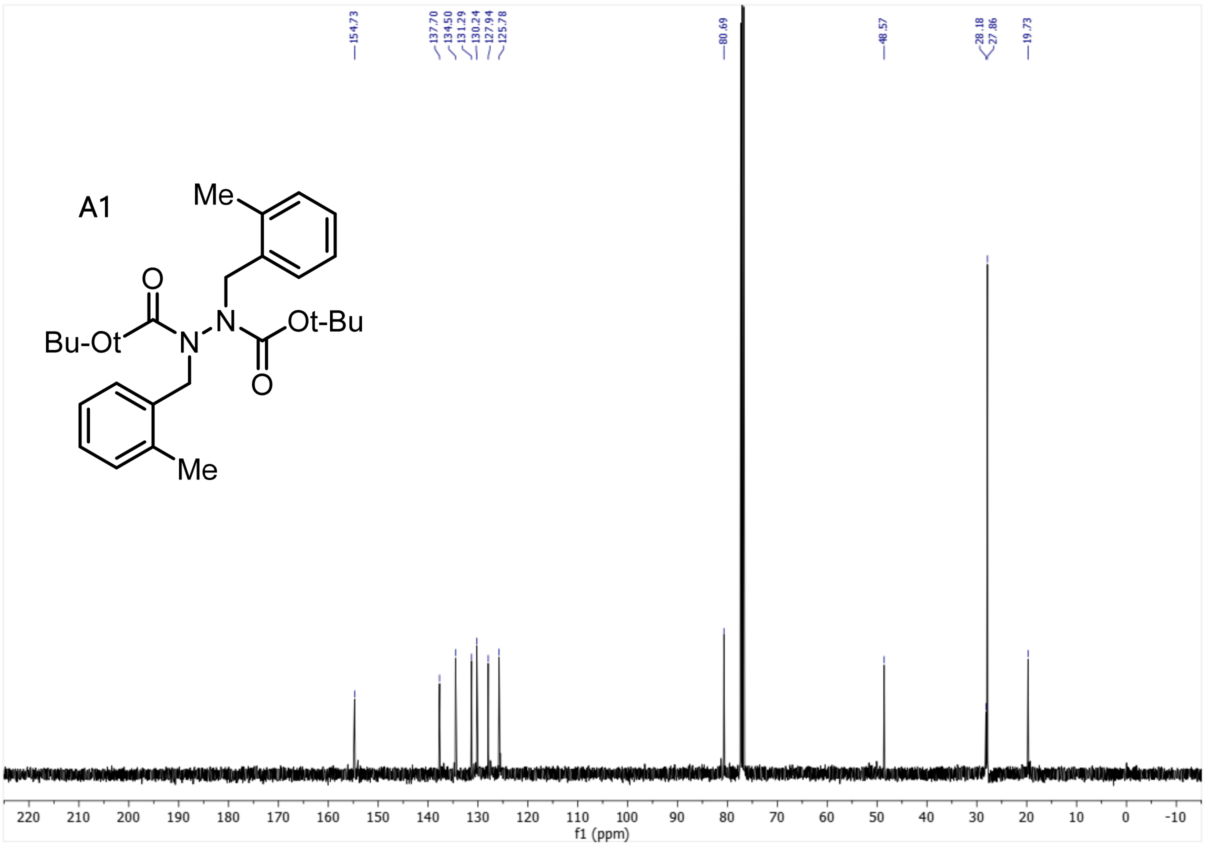

**$^{1}$H NMR** (400 Hz, CDCl3)

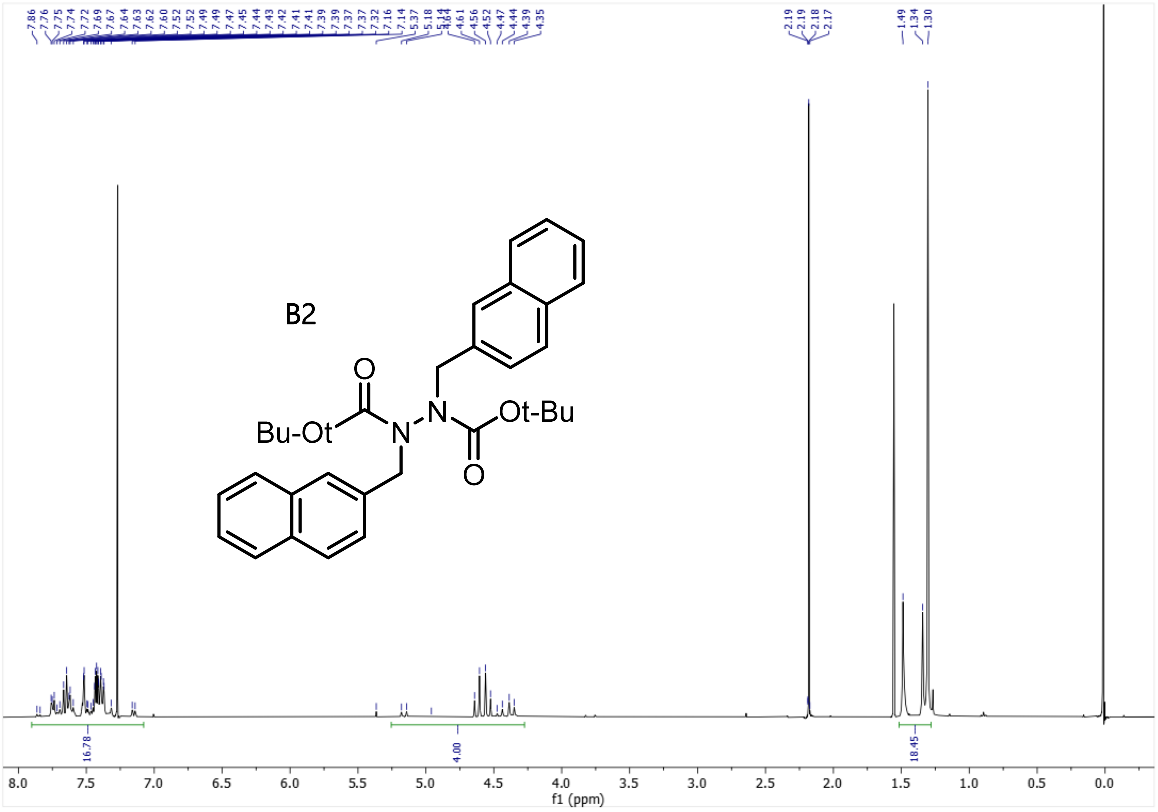


**$^{13}$C NMR** (101 MHz, CDCl3)

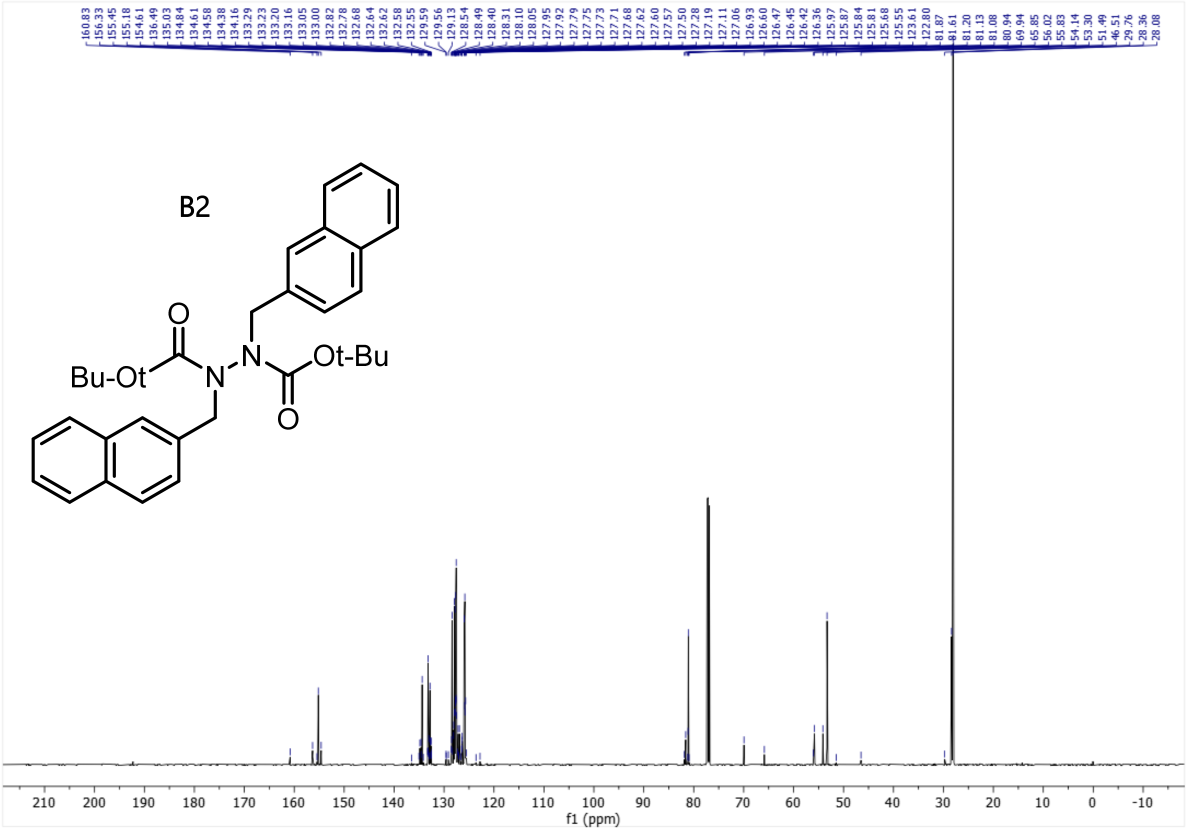

**¹H NMR** (400 Hz, $CDCl_3$)

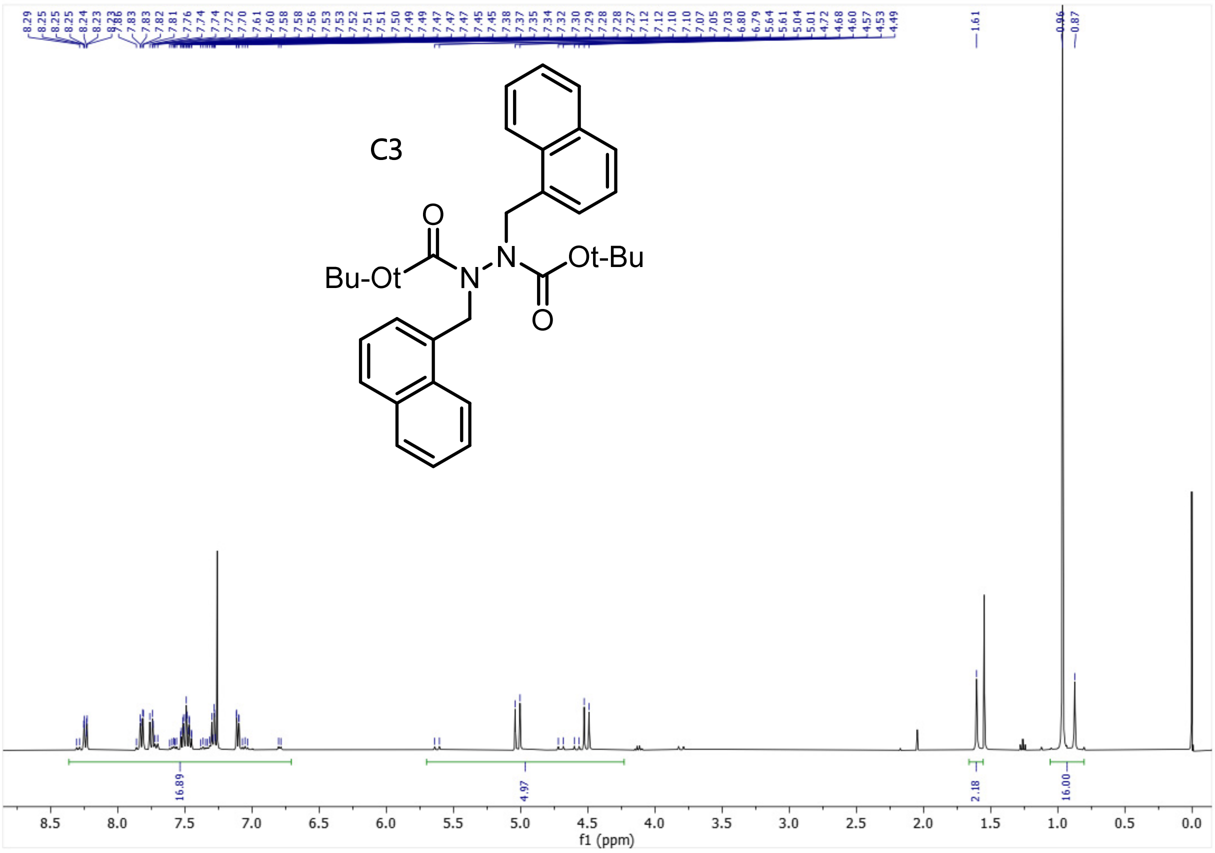


**¹³C NMR** (101 MHz, $CDCl_3$)

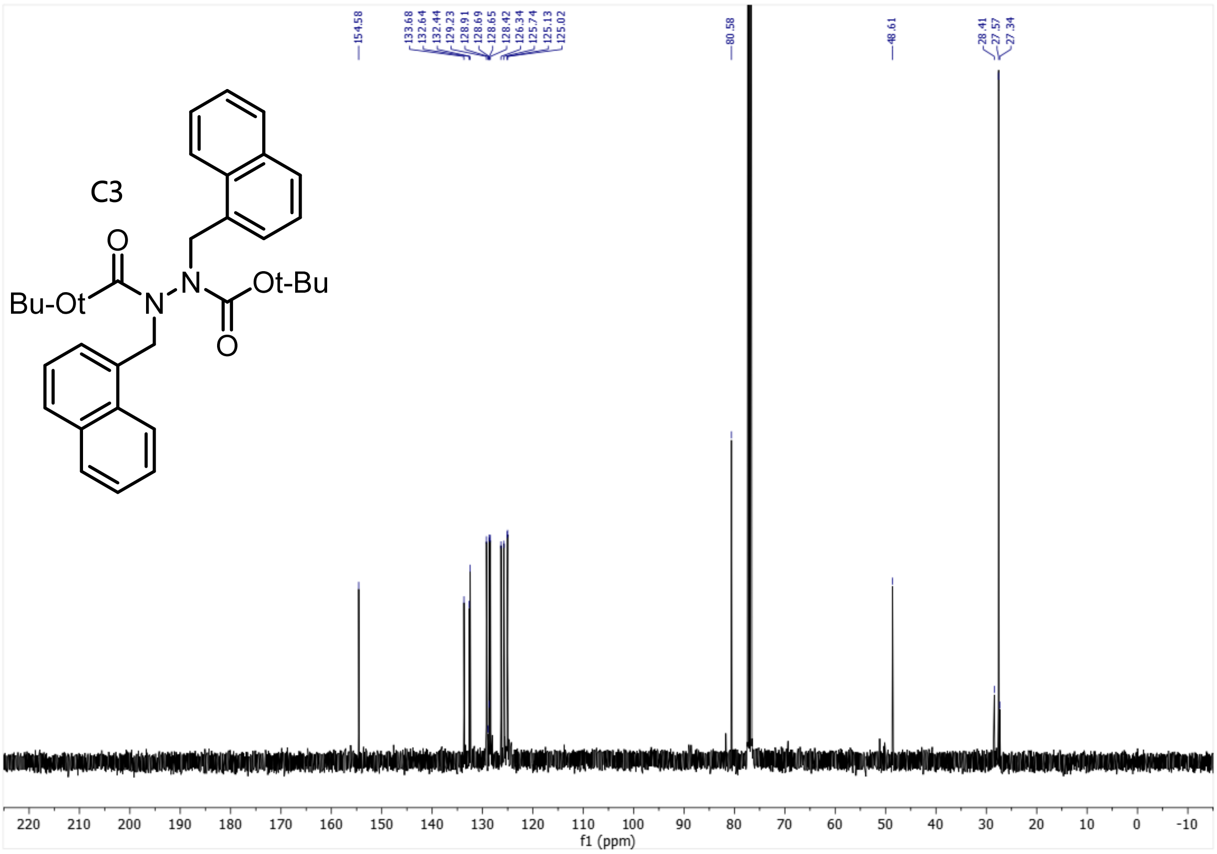

**¹H NMR** (400 Hz, $CDCl_3$)

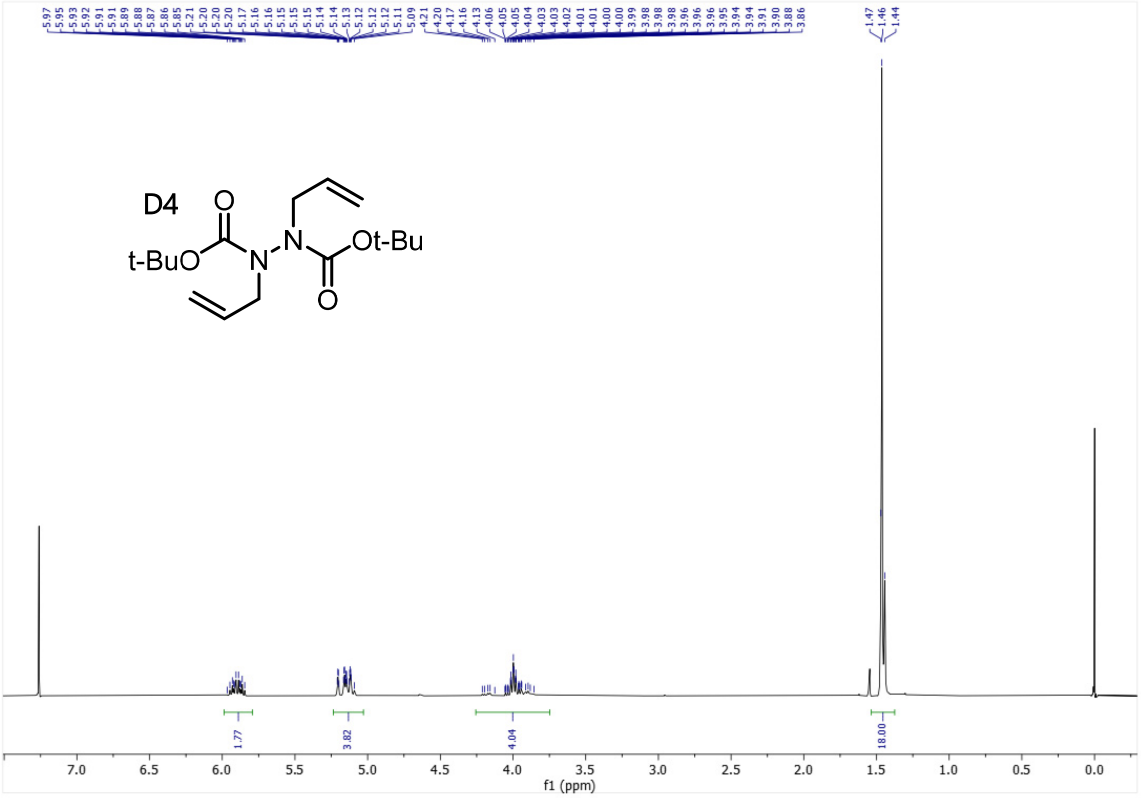


**¹³C NMR** (101 MHz, $CDCl_3$)

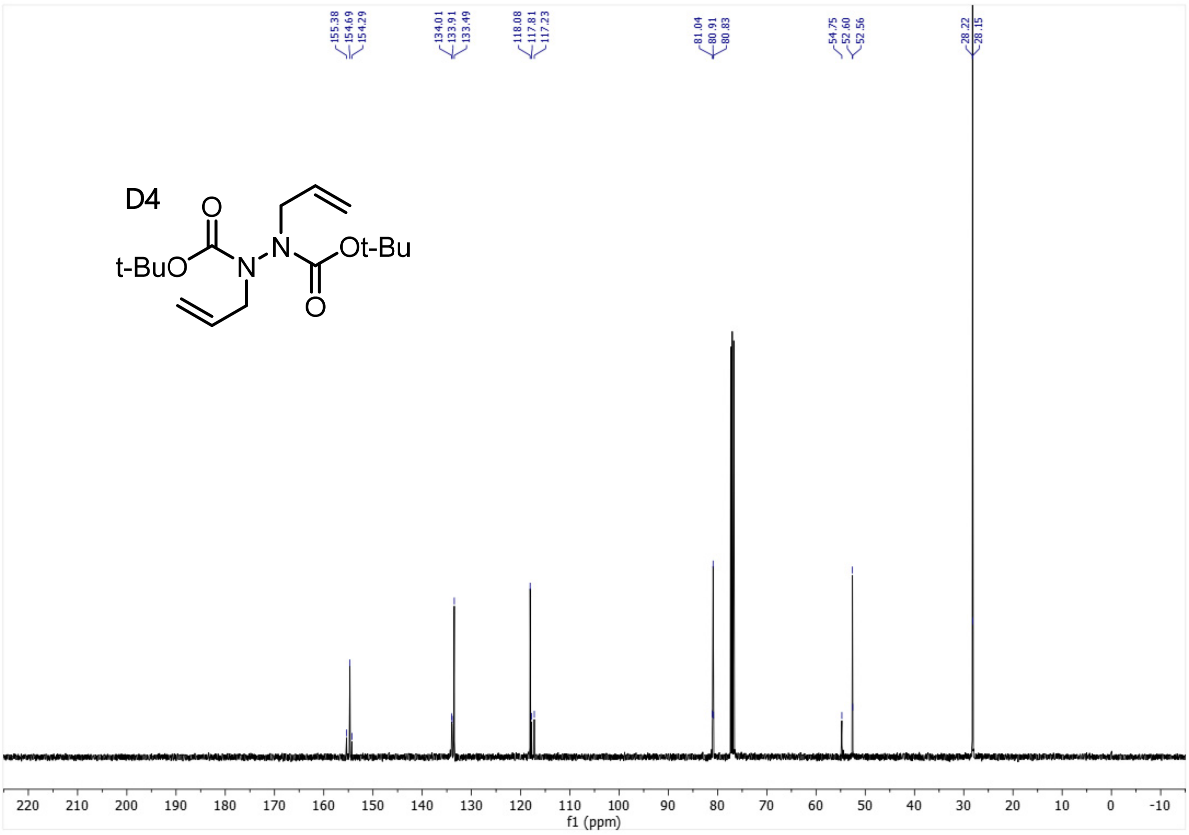

**$^1$H NMR** (400 Hz, CDCl$_3$)

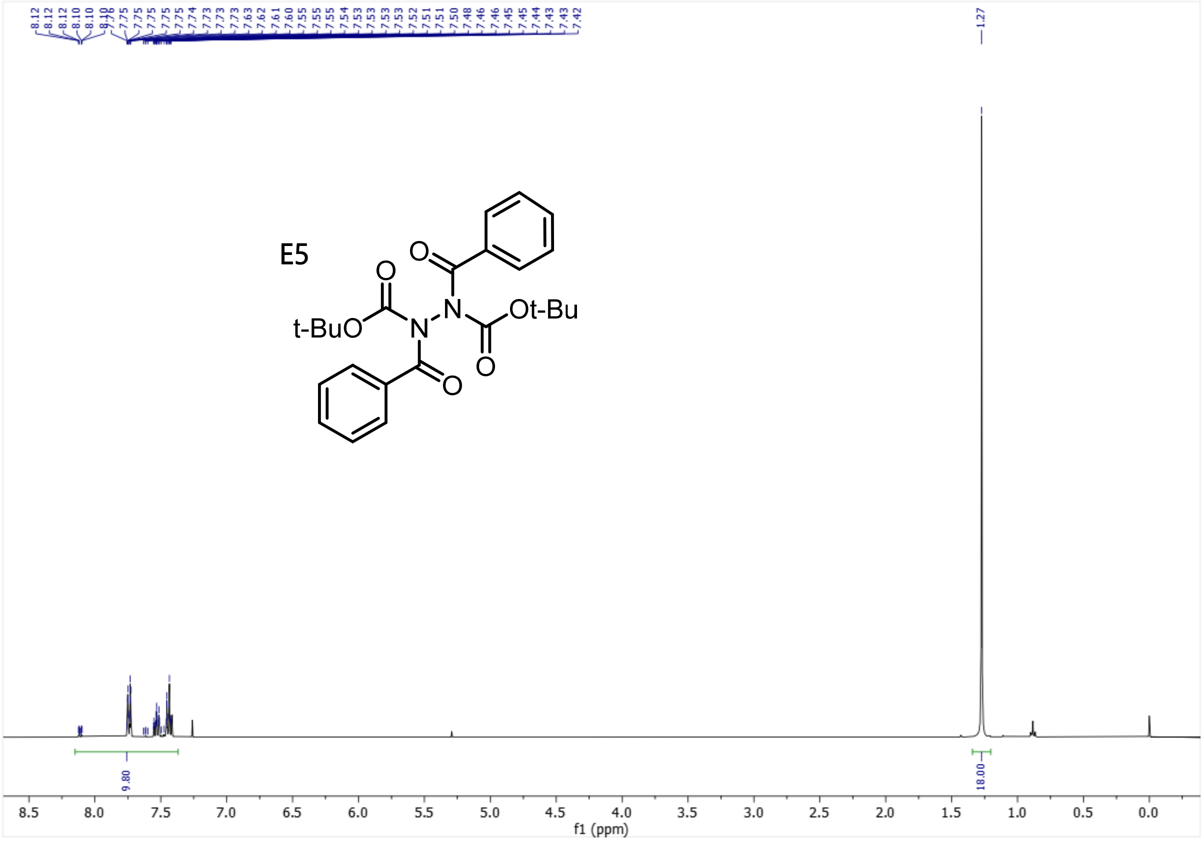

**$^{13}$C NMR** (101 MHz, CDCl$_3$)

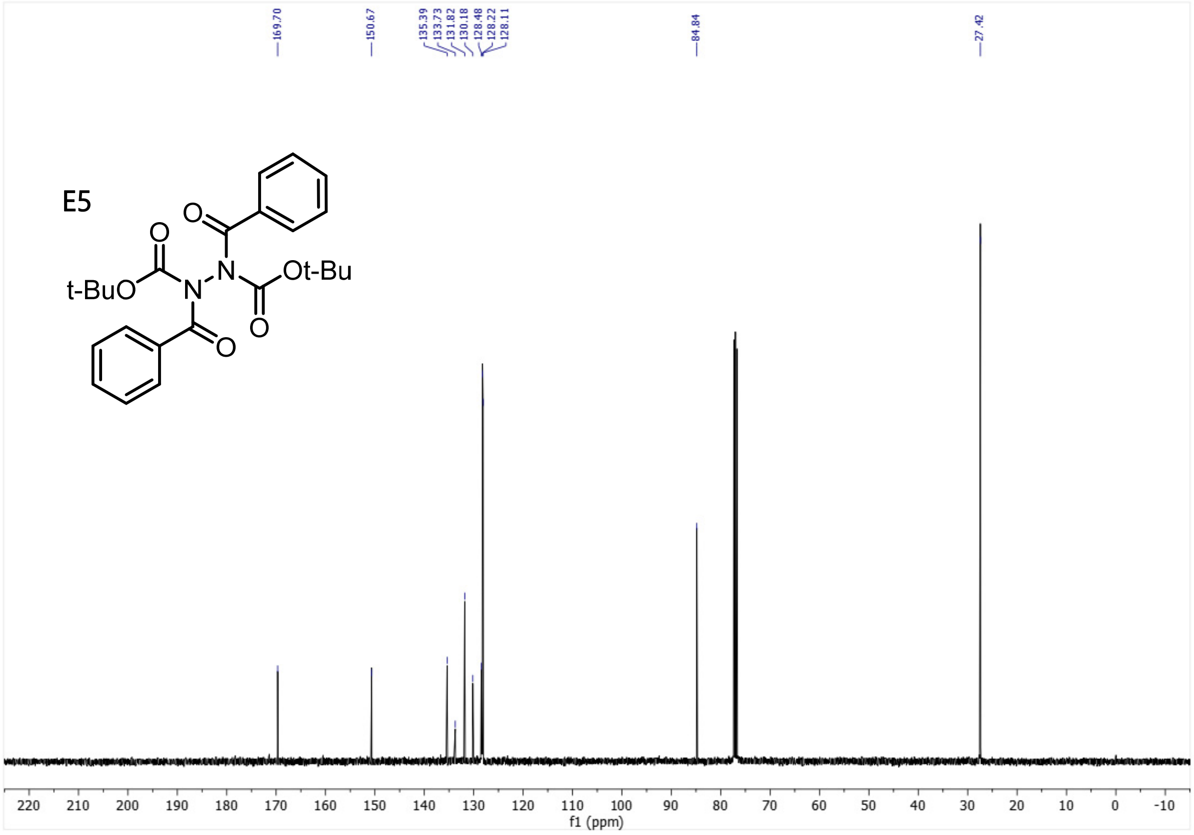

**$^{1}$H NMR** (400 Hz, $CDCl_3$)

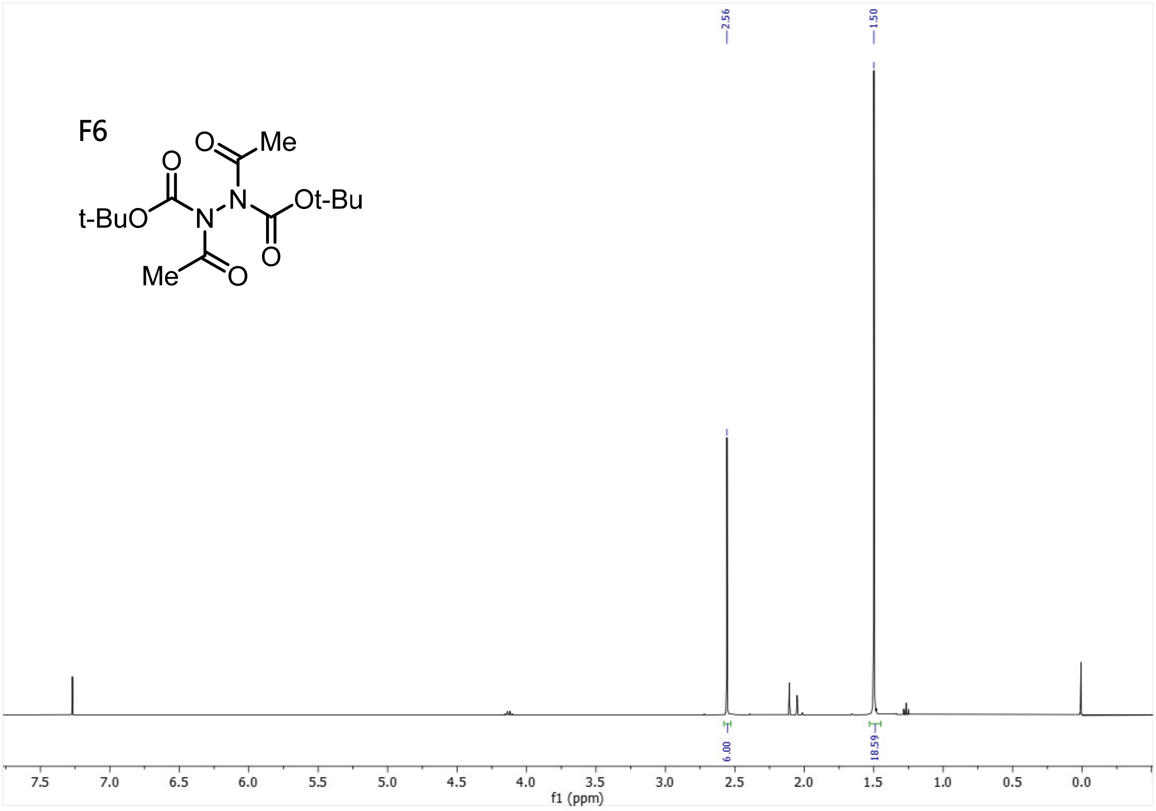


**$^{13}$C NMR** (101 MHz, $CDCl_3$)

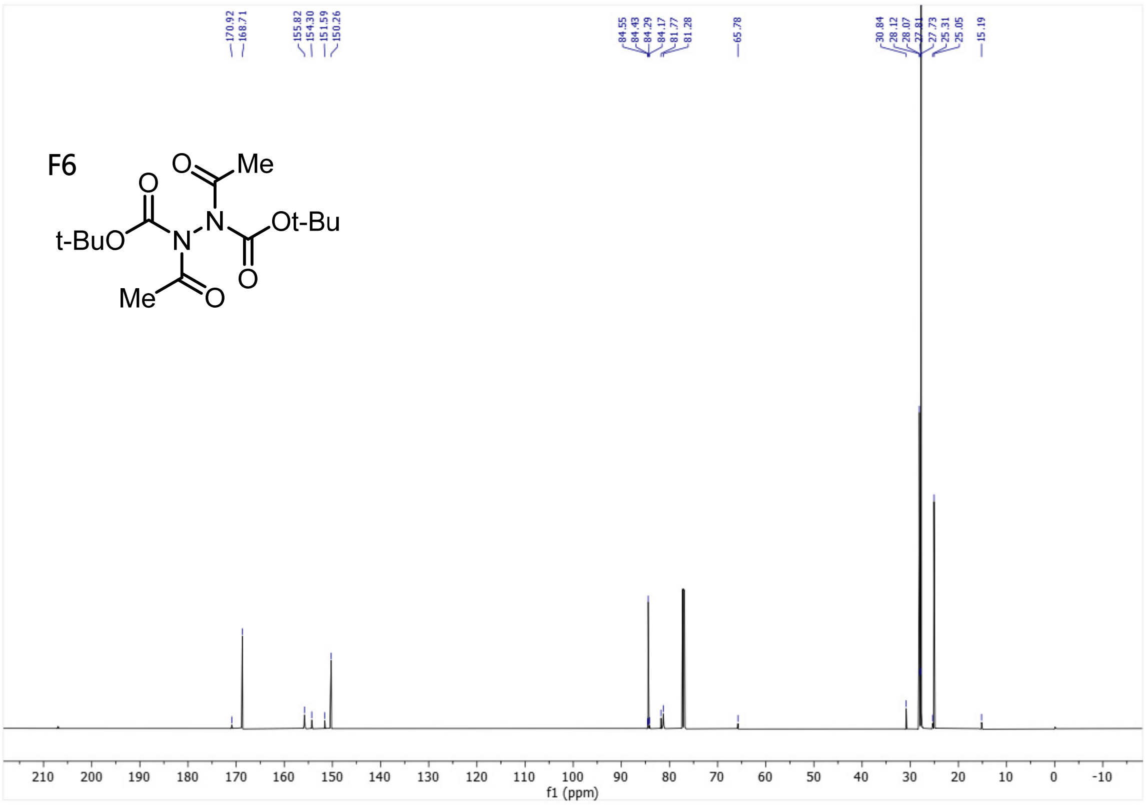

**$^{1}$H NMR** (400 Hz, $CDCl_3$)

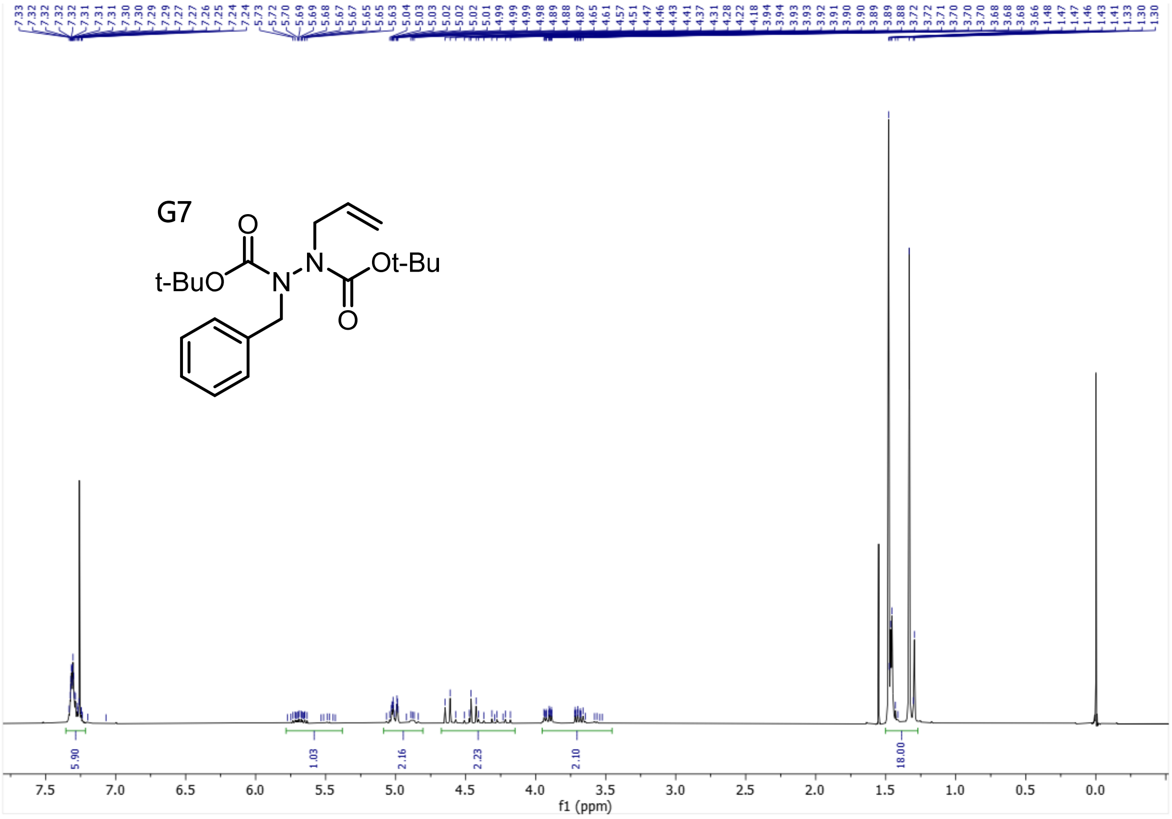


**$^{13}$C NMR** (101 MHz, $CDCl_3$)

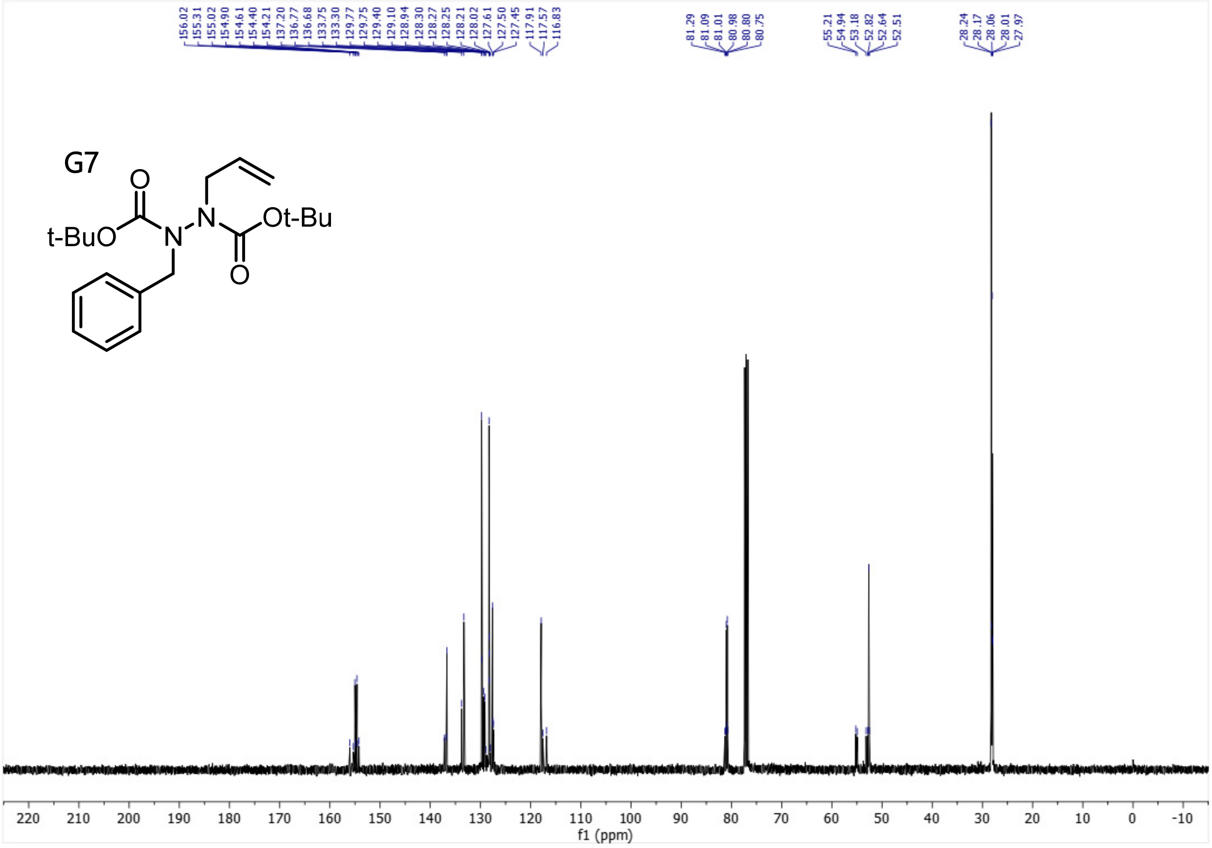

*Materials for HPLC method development*

All solvents were LC grade and were purchased from Sigma-Aldrich (St. Louis, MO, USA). Solutions of samples were prepared in mobile phases.

Analytical HPLC columns were employed: Chiralplak IB (250*4.6 mm ID) and Chiralpak IG-3 (250*4.6 mm ID) from Daicel Corporation (Osaka, Japan), Lux-cellulose 5micron (150*4.6 mm ID) from Phenomenex (Torrance, CA, USA), (R,R)-Whelk-O1 (250*4.6 mm ID) and (R,R)-Whelk-O1 (150*4.6 mm ID) from Regis Technologies (Morton Grove, IL, USA).

*Instrumentation*

A Jasco LC (Jasco Europe, LC, Italy) equipped with dual gradient pumps was employed and connected with UV975 and UV-CD 955 detectors and with a column module. The heating was set by using in-house sheath with a 30°-80°C temperature range. Column temperature was maintained within ± 0.5 °C by means of an electronic controller. For lower temperatures, an ice/water filled Dewar was employed. The uncertainty in temperature measurements can be estimated as ± 0.5 °C.

*Analytical conditions*

**Sample D4**: (R,R) Whelk-O1 CSP (150 x 4.6 mm, L x ID), eluent hexane/2-propanol 90/10, flow rate 1.0 ml/min, UV 254 (weak CD signal at 265 nm: 1$^{st}$ eluted (-), 2$^{nd}$ eluted (+)).

**Sample C3**: Chiralplak IG-3 CSP (250 x 4.6 mm, L x ID), eluent hexane/ethanol 100/5, flow rate 1.0 ml/min, UV 254 (CD signal at 265 nm: 1$^{st}$ eluted (-), 2$^{nd}$ eluted (+)).

**Sample B2**: (R,R) Whelk-O1 CSP (250 x 4.6 mm, L x ID), eluent hexane/2-propanol 98/2, flow rate 1.0 ml/min, UV 254 (CD signal at 230 nm: 1$^{st}$ eluted (-), 2$^{nd}$ eluted (+)).

**Sample A1**: Lux 5 μm Cellulose-1 (150 x 4.6 mm, L x ID), eluent hexane/ethanol 100/1, flow rate 1.0 ml/min, UV 280 (CD signal at 254 nm: 1$^{st}$ eluted (+), 2$^{nd}$ eluted (-)).

**Sample F6**: Chiralplak IG-3 CSP (250 x 4.6 mm, L x ID), eluent hexane/2-propanol 100/1, flow rate 1.0 ml/min, UV 254 (CD signal at 265 nm: 1$^{st}$ eluted (-), 2$^{nd}$ eluted (+)).

**Sample E5**: Chiralplak IB (250 x 4.6 mm, L x ID), eluent hexane/2-propanol 98/2, flow rate 1.0 ml/min, UV 254 (CD signal at 254 nm: 1$^{st}$ eluted (+), 2$^{nd}$ eluted (-)).

*Racemization studies*

Racemization experiments were made by off-column approach. Previously HPLC separated enantiomer of each racemate was dissolved in a closed vial and it was heated at a fixed temperature. Samples were withdrawn at fixed time intervals and analyzed by enantioselective HPLC under the analytical conditions reported in the previous section. The solvent used to dissolve samples was 2-pronanol in all cases. In addition, only for sample 3, some experiments were done also in decalin.

*Enantiomerization studies*

Variable temperature chromatography was performed placing the chiral HPLC column in the specific temperature control module and chromatograms were acquired (n.3 replicate injection for each temperature). Simulations of variable-temperature experimental chromatograms were performed by Auto DHPLC y2k[3–5] based on the stochastic model.

| Sample | $k_1$ | $k_2$ | $\alpha$ | T °C |
|---|---|---|---|---|
| **D4** | 6.65 | 8.21 | 1.23 | 25 |
| **C3** | 0.43 | 1.93 | 4.49 | 25 |
| **B2** | 2.75 | 3.35 | 1.22 | 25 |
| **A1** | 0.80 | 0.99 | 1.12 | 25 |
| **F6** | 2.95 | 4.13 | 1.40 | 10 |
| **E5** | 1.06 | 1.73 | 1.63 | 0 |

*Table S1. Chromatographic parameters of samples after baseline optimized separation.*

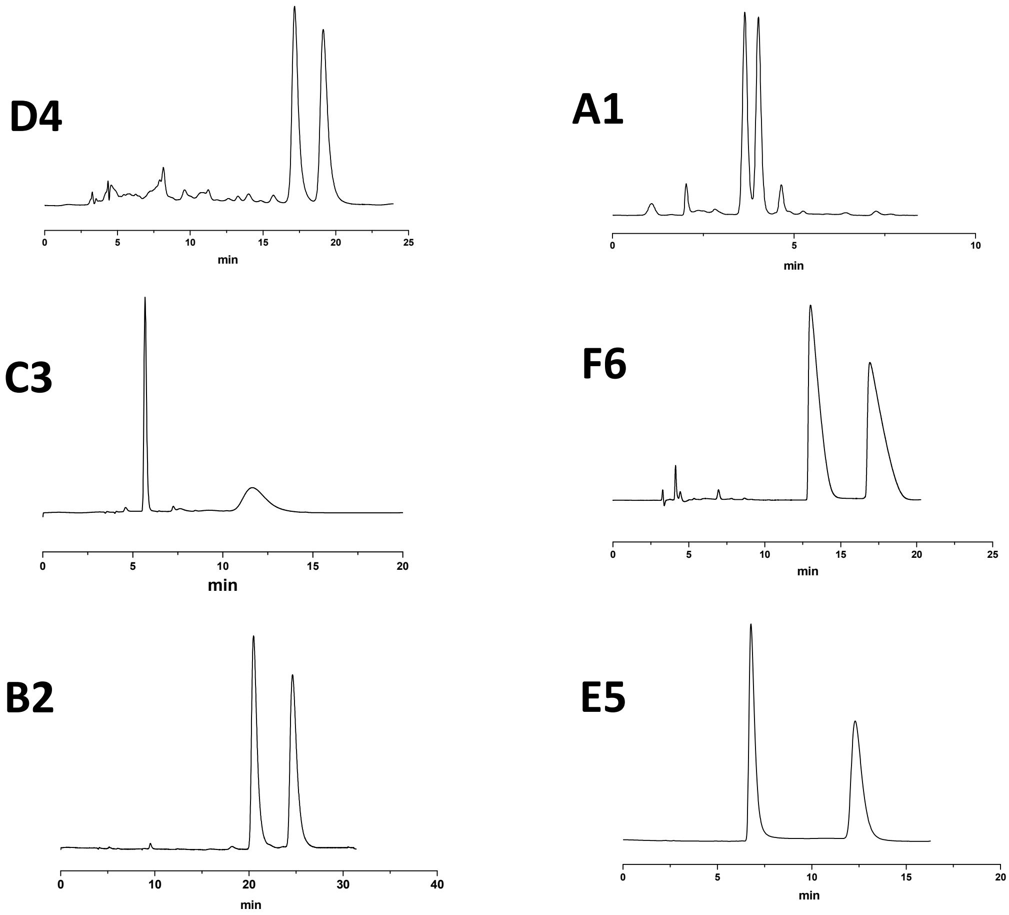


*Figure S1. UV Chromatographic profiles of investigated samples.*

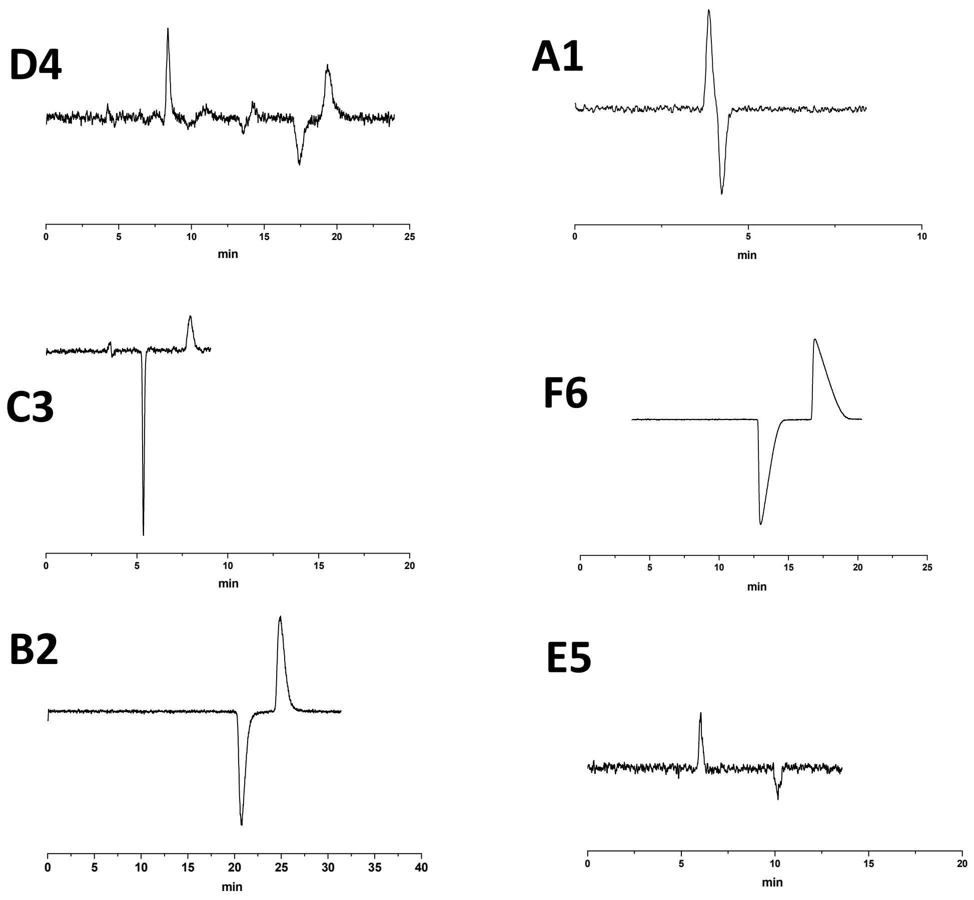


*Figure S2. CD Chromatographic profiles of investigated samples.*

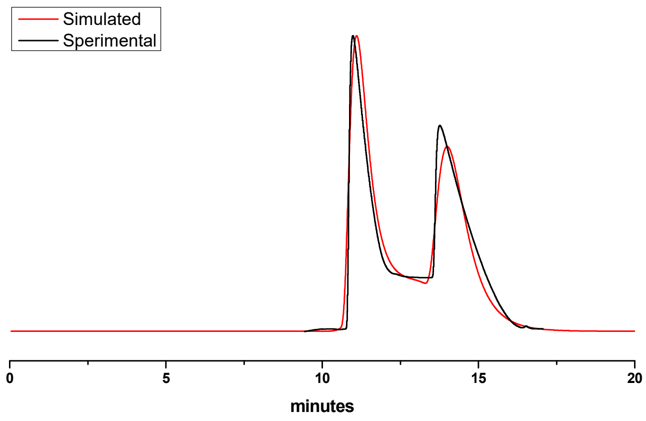


*Figure S3. Experimental (black) and simulated (red) chromatographic profiles of F6 sample at 40°C.*

| Sample | T (°C) | $\Delta G_{enant}^{\#}$ (kcal/mol) | Chromatographic approach |
|---|---|---|---|
| **B2** | 50 | 24.49 [a] | Off-column |
| | 57 | 24.68 | |
| | 62 | 24.73 | |
| | 71 | 25.09 | |
| | 80 | 25.31 | |
| **E5** | 5 | 20.36 [b] | On-column |
| | 10 | 20.37 [b] | |
| | 25 | 20.59 [b] | |

*Table S2. Chromatographic approach for enantiomerization studies of B2 and E5. [a] A value of 24.05 Kcal/mol was found when sample was dissolved in decalin. [b] averaged value between free energy of direct ($\Delta G_{1\text{-}2}^{\#}$) and reversed ($\Delta G_{2\text{-}1}^{\#}$) enantiomerization process.*

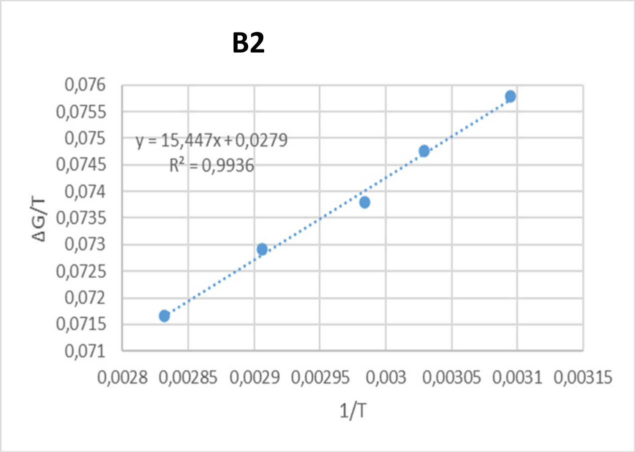


| Temperature | ΔG | 1/T(°K) | ΔG/T |
|---|---|---|---|
| 50 | 24,49 | 0,0031 | 0,0758 |
| 57 | 24,68 | 0,0030 | 0,0748 |
| 62 | 24,73 | 0,0030 | 0,0738 |
| 71 | 25,09 | 0,0029 | 0,0729 |
| 80 | 25,31 | 0,0028 | 0,0717 |

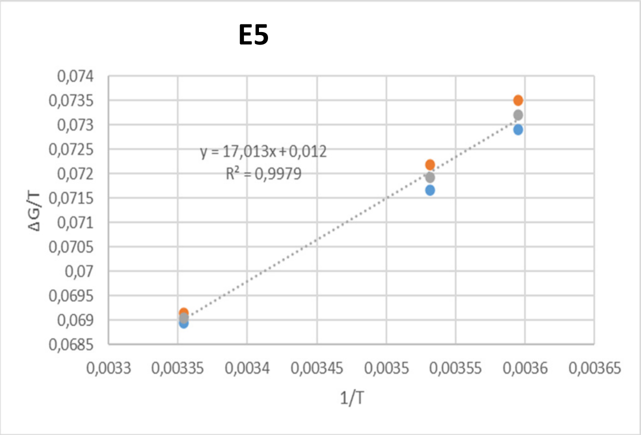


| Temperature | $\Delta G_{averaged}$ | 1/T(°K) | $\Delta G_{averaged}$/T |
|---|---|---|---|
| 25 | 20,59 | 0,00335 | 0,06905 |
| 10 | 20,37 | 0,00353 | 0,07193 |
| 5 | 20,36 | 0,00360 | 0,07320 |

*Figure S4. Eyring plots of B2 and E5.*

## *Computational study*

### *General information*

The surfaces were visualized with the Visual Molecular Dynamics[6] (VMD) software. Instead, the UCSF Chimera[7] software was utilized to visualize geometries and orbitals.

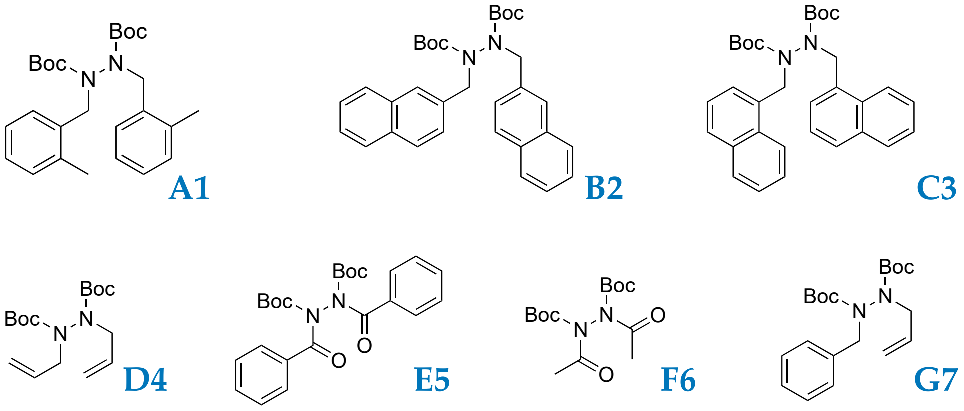


*Figure 5. Studied molecules*

### *Conformational searches and refinement of the ensembles*

An initial conformational search was performed for each of the seven hydrazides using the CREST[8] software utility (version 2.11.1), which is based on the xTB[9] engine (version 6.4.1). The obtained conformational ensembles were further refined with CENSO[10] (version 1.2.0). Each one of the seven structures was initially subjected to this conformational searches at 298.15 K at the GFN2-xTB[11] level, discarding all conformers with an energy higher than the predefined cutoff values for the energy (6 kcal/mol relative to the lowest-energy conformer) and the root mean square difference of cartesian coordinates (RMSD; 0.125 Å).

Visual inspection of the seven ensembles obtained by CREST algorithm showed that all ensembles contained both axial epimers regardless of the axial configuration of the starting structure.

The results obtained in these conformational searches are reported below.

| | Number of conformers | % Population of the lowest energy conformer |
|---|---|---|
| A1 | 93 | 67.0 |
| B2 | 241 | 22.2 |
| C3 | 125 | 46.9 |
| D4 | 71 | 24.7 |
| E5 | 86 | 30.1 |
| F6 | 66 | 9.8 |
| G7 | 115 | 38.1 |

*Table S3. Results of the conformational studies performed with CREST at the GFN2-xTB level using the default thresholds.*

As can be seen, the high number of conformers obtained for all 7 molecules under investigation is indicative of their high structural flexibility. This observation led to the investigation of the effect of different RMSD thresholds on the population for each substrate. Therefore, for each conformer a second CREST run was carried out using a larger RMSD threshold of 0.250 Å, instead of the default value of 0.125 Å used in the first run. Each conformer of the ensemble obtained with an RMSD of 0.250 Å was compared with all the conformers of the ensemble obtained with an RMSD of 0.125 Å. In case the difference in energy was below 0.05 kcal/mol, the two conformers were considered equivalent. Here below is reported the number of conformers of one ensemble contained in the other and *vice versa* showing that the conformational searches run with either the RMSD threshold at 0.125 Å or at 0.250 Å resulted in almost identically populated ensembles. Also, most of the structures were visually superimposable, thus confirming the data previously gathered.

| | RMSD Threshold 0.125 Å | RMSD Threshold 0.250 Å |
|---|---|---|
| A1 | 92 out of 93 (98.92%) | 89 out of 92 (96.74%) |
| B2 | 233 out of 241 (96.68%) | 205 out of 208 (98.56%) |
| C3 | 120 out of 125 (96.00%) | 112 out of 117 (95.73%) |
| D4 | 69 out of 71 (97.18%) | 91 out of 124 (73.39%) |
| E5 | 84 out of 86 (97.67%) | 77 out of 80 (96.25%) |
| F6 | 60 out of 66 (90.91%) | 58 out of 59 (98.31%) |
| G7 | 109 out of 115 (94.78%) | 99 out of 112 (88.39%) |

*Table S4. Comparison between the ensembles generated using two different RMSD thresholds.*

The seven conformer ensembles obtained with CREST in the previous step, were refined to DFT-theory level using CENSO (version 1.2.0) framework interfaced with ORCA[12] (version 5.0.1) according to the following protocol:

1. part0 *cheap prescreening*: b97-d3[13]/def2-SV(P) // GFN2-xTB (Input geometry)
2. part1 *prescreening*: r$^2$scan-3c[14]+ C-PCM[hexane] + GmRRHO[15](GFN2[ALPB[16]]-bhess[17]) // GFN2-xTB (Input geometry)
3. part2 *optimization*: r$^2$scan-3c + C-PCM[hexane] + GmRRHO(GFN2[ALPB]-bhess) // r$^2$scan-3c[SMD]

Thermochemistry data were obtained at 298.15 K.

The following table shows the CENSO refinement results for the conformer ensembles obtained from CREST, highlighting the pruning that occurred at the three different steps. At each part the conformers are analyzed and those with a relative energy greater than the default (for *part0* 4.0 kcal/mol, *part1* 3.5 kcal/mol and *part2* 2.5 kcal/mol). For the last two parts, the free energy was calculated via the mRRHO approximation. Furthermore, to consider the dispersion of the various

conformers' energy, in part2 a Spearman's correlation coefficient automatically generated was taken into account to smooth the energy threshold.

| | Input ensemble (CREST) | After part0 | After part1 | After part2 | % Population of lowest conf. |
|---|---|---|---|---|---|
| A1 | 93 | 89 | 78 | 48 | 31.5 |
| B2 | 241 | 177 | 127 | 96 | 4.5 |
| C3 | 125 | 72 | 37 | 31 | 29.4 |
| D4 | 71 | 47 | 37 | 30 | 17.0 |
| E5 | 86 | 60 | 59 | 51 | 11.2 |
| F6 | 66 | 40 | 40 | 35 | 7.5 |
| G7 | 115 | 83 | 62 | 50 | 13.1 |

*Table S5. Pruning process of CENSO framework after each part of the process.*

Then, for each molecule, the lowest-energy conformer produced by CENSO was optimized using Gaussian 16 (rev. A.03)[18] at the DFT level with the chemical model ωB97x-D/6-31G(d) and implicit solvation C-PCM in n-hexane. These parameters were chosen after previous works on similar compounds[19]. Thermochemical corrections were obtained from frequency calculations at the same level for a state of 1 atm and 298.15 K.

As reported in Table 4, the comparison of several structural parameters showed good consistency between the CENSO energy lowest-lying conformer its Gaussian DFT optimized geometry

| | A1 | | B2 | | C3 | | D4 | |
|---|---|---|---|---|---|---|---|---|
| | CENSO | GAUSSIAN | CENSO | GAUSSIAN | CENSO | GAUSSIAN | CENSO | GAUSSIAN |
| ϑ1 | 2.29 | -0.75 | 4.91 | 9.26 | 0.59 | -1.26 | -8.41 | -10.41 |
| ϑ2 | 2.33 | -0.75 | 4.94 | 8.84 | 0.59 | -1.25 | -8.39 | -10.41 |
| ϑ3 | 83.33 | 77.89 | -67.64 | -61.55 | 81.61 | 77.58 | 63.93 | 62.73 |
| ϑ4 | 75.75 | 80.37 | -83.69 | -91.12 | 79.68 | 81.69 | 91.35 | 96.91 |
| ϑ5 | 177.67 | 179.87 | 173.66 | 171.67 | 177.79 | 179.23 | -171.47 | -171.38 |
| ϑ6 | 177.60 | 179.87 | 174.21 | 171.35 | 177.79 | 179.23 | -171.44 | -171.38 |
| r1 | 5.51 | 5.45 | 5.41 | 5.34 | 5.51 | 5.46 | 5.35 | 5.31 |
| r2 | 3.53 | 3.52 | 3.53 | 3.52 | 3.53 | 3.52 | 3.53 | 3.52 |
| r3 | 3.53 | 3.52 | 3.53 | 3.52 | 3.53 | 3.52 | 3.53 | 3.52 |
| r4 | 1.38 | 1.37 | 1.38 | 1.38 | 1.38 | 1.37 | 1.38 | 1.38 |
| r5 | 1.38 | 1.38 | 1.39 | 1.38 | 1.38 | 1.38 | 1.38 | 1.38 |
| r6 | 1.38 | 1.38 | 1.39 | 1.38 | 1.38 | 1.38 | 1.38 | 1.38 |
| r7 | 1.46 | 1.46 | 1.46 | 1.46 | 1.46 | 1.46 | 1.46 | 1.46 |
| r8 | 1.46 | 1.46 | 1.46 | 1.46 | 1.46 | 1.46 | 1.46 | 1.46 |

| | E5 | | F6 | | G7 | |
|---|---|---|---|---|---|---|
| | CENSO | GAUSSIAN | CENSO | GAUSSIAN | CENSO | GAUSSIAN |
| ϑ1 | 6.50 | 4.35 | 0.43 | 1.48 | -4.76 | -8.31 |
| ϑ2 | -8.18 | -5.82 | 0.39 | 1.48 | -7.80 | -11.52 |
| ϑ3 | 87.87 | 87.70 | -86.43 | -84.12 | 66.05 | 62.11 |
| ϑ4 | 90.74 | 90.27 | -87.79 | -89.03 | 86.56 | 94.64 |

| | | | | | | |
|---|---|---|---|---|---|---|
| ϑ5 | 21.43 | 19.01 | 178.33 | 179.24 | -171.93 | -170.04 |
| ϑ6 | -22.86 | -20.29 | 178.37 | 179.24 | -173.26 | -172.92 |
| r1 | 3.44 | 3.43 | 5.43 | 5.41 | 5.37 | 5.32 |
| r2 | 2.68 | 2.67 | 3.52 | 3.51 | 3.53 | 3.52 |
| r3 | 2.66 | 2.65 | 3.52 | 3.51 | 3.53 | 3.52 |
| r4 | 1.38 | 1.37 | 1.39 | 1.38 | 1.34 | 1.38 |
| r5 | 1.42 | 1.40 | 1.41 | 1.41 | 1.38 | 1.38 |
| r6 | 1.42 | 1.40 | 1.41 | 1.41 | 1.38 | 1.38 |
| r7 | 1.41 | 1.41 | 1.42 | 1.42 | 1.46 | 1.41 |
| r8 | 1.42 | 1.42 | 1.42 | 1.42 | 1.46 | 1.42 |

*Table S6. Geometrical parameters comparison for the CENSO low-lying conformer and its DFT optimized geometry.*

| Geometrical Parameter definition | |
|---|---|
| θ1 | 3-2-5-1 |
| θ2 | 4-1-6-2 |
| θ3 | 5-1-2-6 |
| θ4 | 3-1-2-4 |
| θ5 | 1-2-6-8 |
| θ6 | 2-1-5-7 |
| r1 | 7-8 |
| r2 | 7-2 |
| r3 | 8-1 |
| r4 | 1-2 |
| r5 | 1-5 |
| r6 | 2-6 |

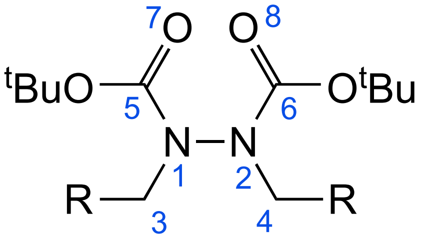


*Figure S6. Geometrical parameter definition..*

All the DFT optimized structures show nearly perfectly planar $sp^2$-hybridized nitrogen atoms, as can be seen from dihedral angles ϑ1 and ϑ2 in Table 4. In addition, the best conformer for molecules A1, B2, C3, D4 and F6 has an almost perfect C2 symmetry.

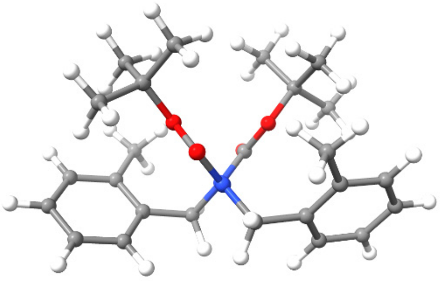

**A1**

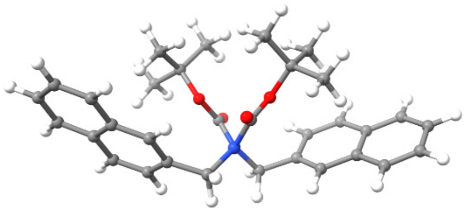

**B2**

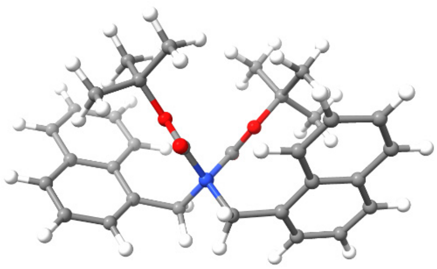

**C3**

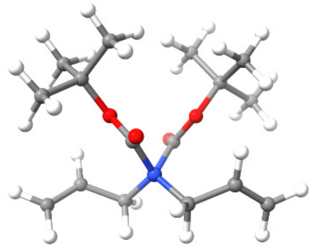

**D4**

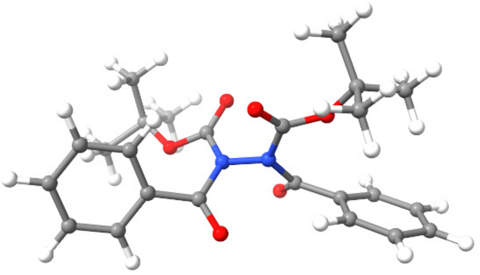

**E5**

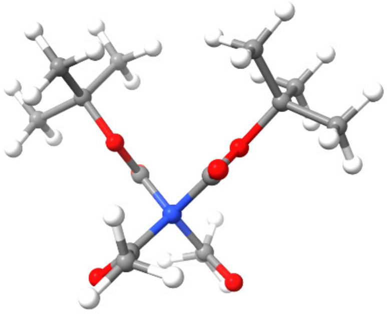

**F6**

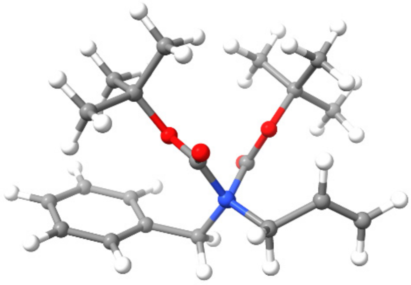

**G7**

*Figure S7. Best conformers geometry.*

Three of these structures (B2, D4 and G7) have a $\vartheta3$ dihedral angle around the N—N bond of about 60°, while for the other four structures this angle is closer to the orthogonal arrangement, varying from 77 to about 88°. To accurately describe noncovalent interactions, the NCI index was employed using NCIPLOT[20] software. The following color maps encode the type of contribution, blue for attractive, red for repulsive and green for van der Waals interactions.

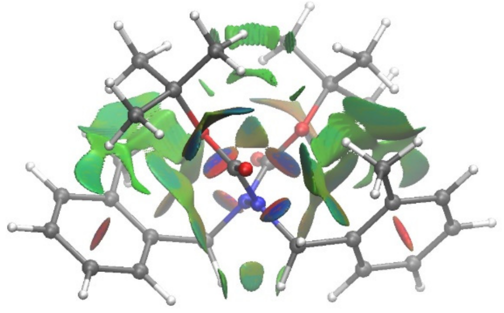

**A1**

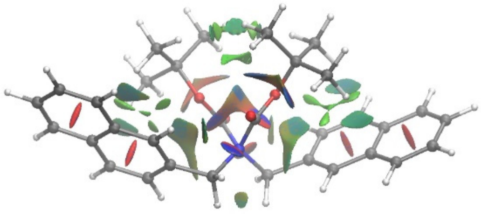

**B2**

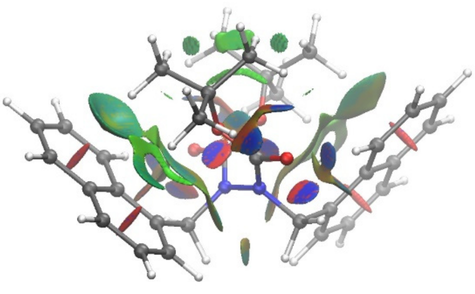

**C3**

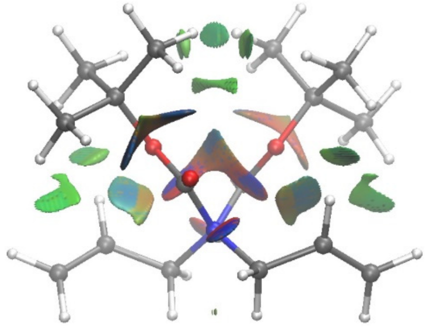

**D4**

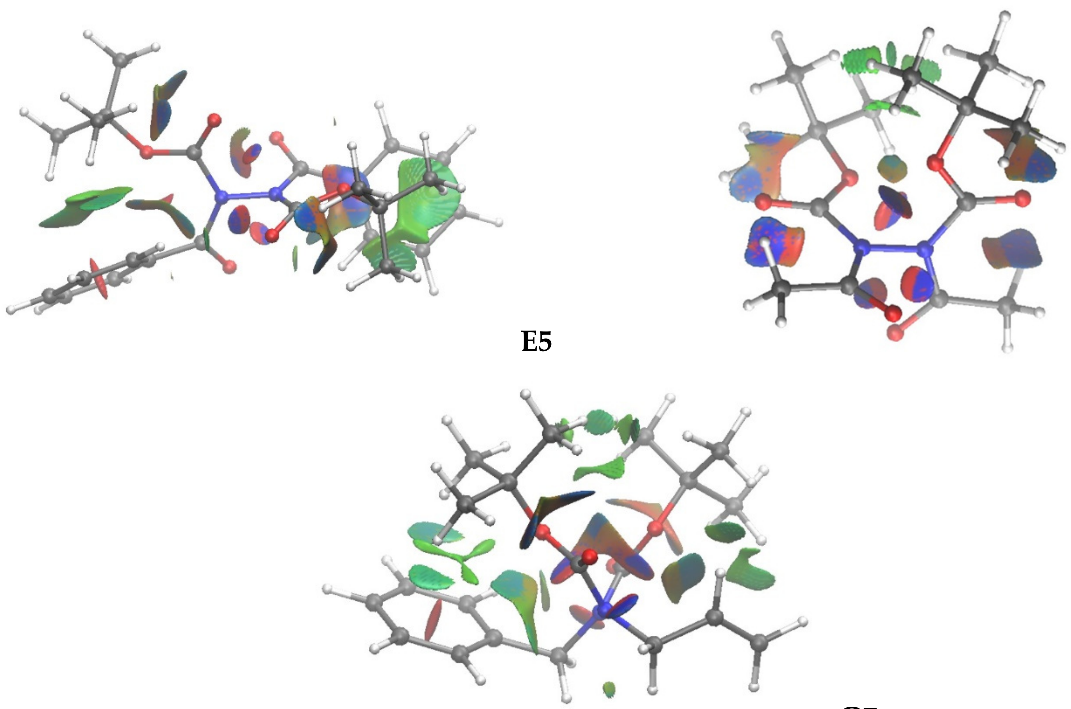

E5 F6 G7

*Figure SS8. NCI-index surfaces.*

As can be seen in Figure 4, these favored conformations appear to be preferred due to two main non-covalent intramolecular interactions: (a) an interaction between one or two methyl protons of one *t*-butyl group with the π-system of an aromatic substituent on the opposite nitrogen atom (when present like in **B2** and **C3**) and (b) the non-covalent interaction between protons of opposite BOC groups (as in **F6**). Another important parameter that is observed in all the molecules but E5 is the relative orientation of the carbonyl groups of the BOCs. This parameter can be confirmed by the distances of those two oxygen atoms (r1) which fluctuates around 5 Å, and by comparing the dihedral angles $\vartheta 4$ and $\vartheta 5$, it's possible to conclude that all these structures are in s-*trans* configuration (regarding the NNCO fragment). The E5 structure represents an exception from these structural parameters, its NNCO fragments are in s-*cis* configuration. These observations are also confirmed by looking at r2 and r3 parameters whose convey the distance of the oxygen atom and the further nitrogen atom.  In Table 5 dipole momentum of different conformers is listed with the associated ΔG that was calculated at the ωB97x-D/6-31G(d) DFT-level considering the implicit *n*-hexane solvation with CPCM theory. Structures with both BOC fragments in s-*cis* configuration are noted as ***inside***, as opposed to s-*trans* as ***outside*** (Figure 5). The CREST--CENSO protocol in all cases but E5 afforded as the best conformer one with both the carbonyls in the "outside" disposition. To obtain the structures with the opposite disposition, the previously optimized structures were distorted by a manual 180° rotation of both the $\vartheta 4$ and $\vartheta 5$ dihedral angles. These distorted geometries were then optimized at the same level. It is worth of note that all the manually generated geometries show higher energies than the conformations obtained by the CREST-CENSO protocol.

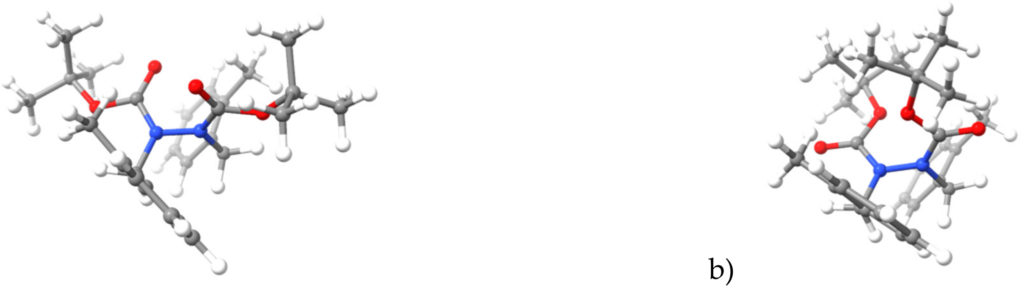

a) b)

*Figure S9. Configuration of BOC fragments: a) inside; b) outside.*

| Dipole momentum | Inside | Outside | ΔG kcal/mol (In-Out) |
|---|---|---|---|
| A1 | 1.9848 | 0.9121 | +5.85 |
| B2 | 1.5692 | 0.8129 | +8.21 |
| C3 | 1.9374 | 0.8278 | +8.47 |
| D4 | 1.5456 | 0.8058 | +3.32 |
| E5 | 2.1177 | 5.2156 | -1.75 |
| F6 | 2.7641 | 5.7090 | +3.92 |
| G7 | 1.7223 | 0.6977 | +5.43 |

*Table S7. Dipole momentum of the optimized structures. Green structures generated from CREST-CENSO protocol. Blue manually generated by manually rotating by 180° the ϑ4 and ϑ5 dihedral angles of the structures.*

From the results shown in Table 5, it is not possible to conclude that all the structures tend to minimize the dipole momentum, in fact for F6 the lowest energy *inside* conformation has the highest dipole momentum. Probably thanks to the gaining in NCIs between methyl groups and carbonyl oxygen atoms (as visible in Figure 4, **F6**). From the visualization of the molecular orbitals of the molecules, it is possible to exclude any type of conjugation between the two nitrogen atoms. HOMO orbitals of all molecules are shown in Figure 3 and most of them are localized on the aromatic system (when present), otherwise it is possible to observe a conjugation of the carbamate system [NC(=O)O].

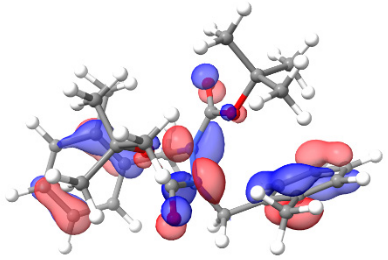

**A1**

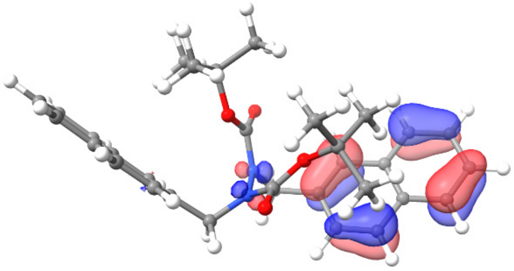

**B2**

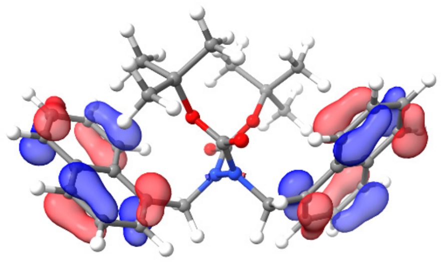

**C3**

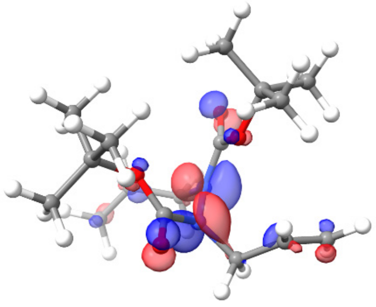

**D4**

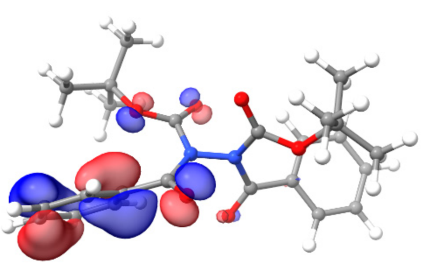

E5

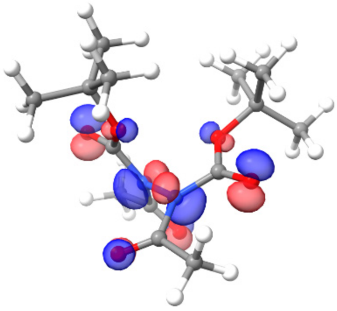

F6

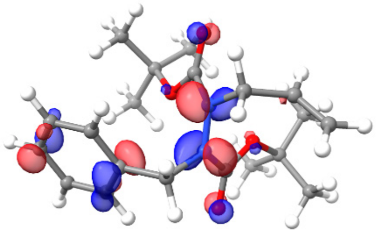

G7

*Figure S10. HOMO molecular orbitals of each low-lying conformer.*

### *TS search for the N—N axial epimerization*

The following step was the quantification of the rotational barrier around the σ N-N bond. To achieve this, an initial analysis of the PES was performed by a relaxed 360° scan of the ϑ3 dihedral (Figure 2) with a 10° step in both clockwise (here noted as **C**) and counterclockwise (**CC**) directions. Each scan allows to obtain two maximum energy geometries that can be used as a good starting guess geometry for the optimization towards the actual transition state (TS) stationary point. To perform these scans in an acceptable time, the DTF-level was lowered to ωB97x-D/3-21G. The following figures show the energy plot for both the scan of each molecule:

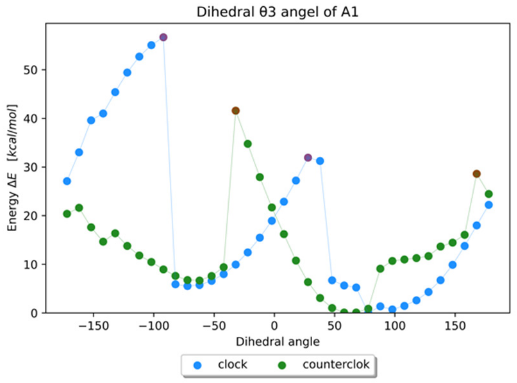


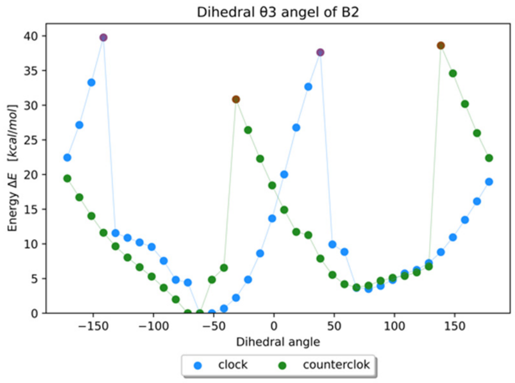

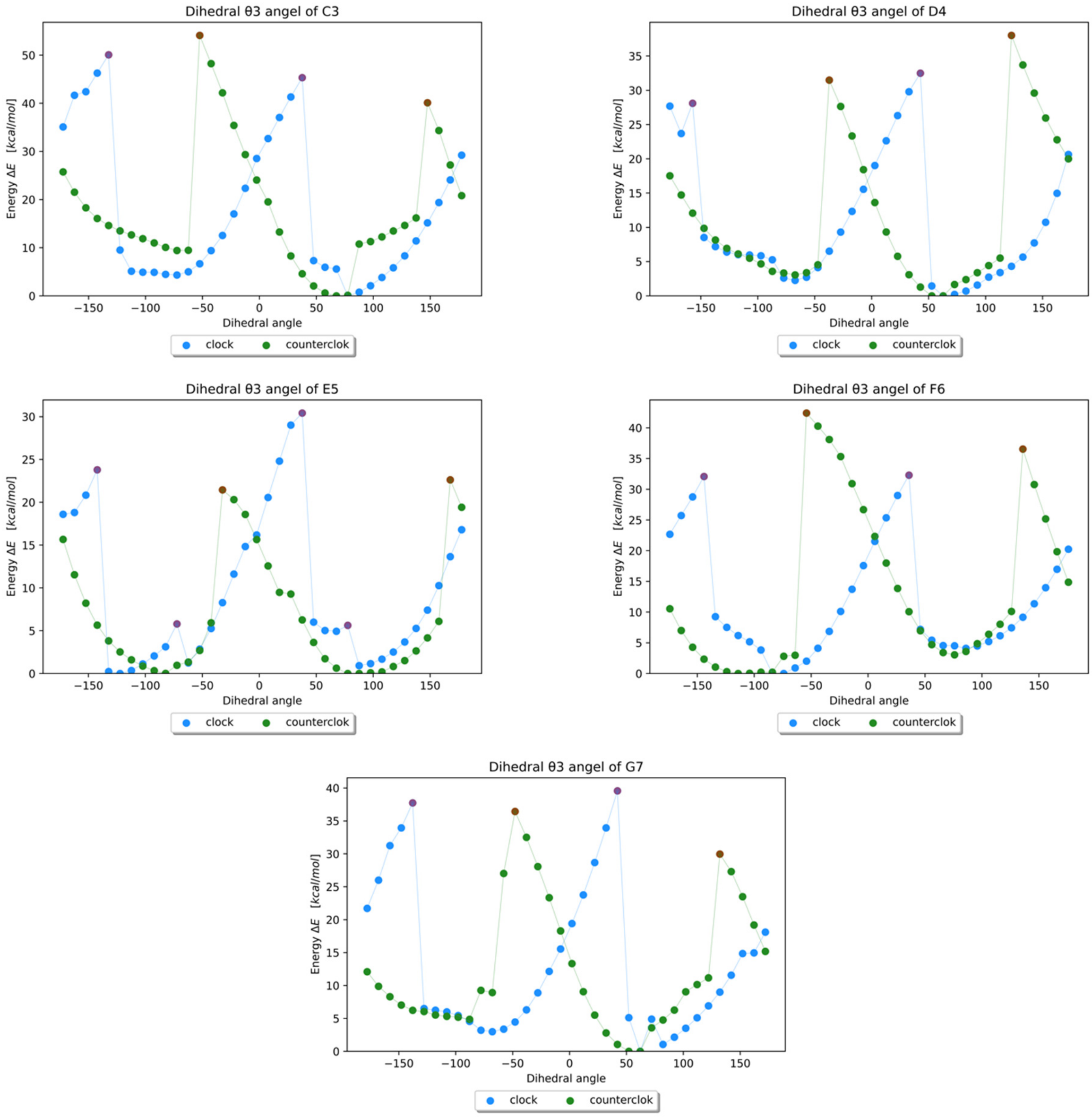


*Figure S11. Scans of the dihedral θ3 angle.*

From each maximum so obtained, an initial optimization had been performed at the ωB97x-D/6-31G(d) level keeping the ϑ3 dihedral angle frozen at the initial value. This allows to acquire a conceivably better candidate for the saddle point search algorithm (Berny optimization). After each of these optimizations, a vibrational frequency calculation was performed at the same level of theory to check for the presence of only a single negative normal mode representing the "epimerization" displacement. Unfortunately, with this protocol only a very few structures have been found with the correct negative frequency.

A modified protocol was therefore attempted:

1. Partial optimization of the maximum structure arising from the scan at ωB97x-D/3-21G with the ϑ3 dihedral angle frozen and subsequent diagonalization of the hessian matrix.
2. If the sought frequency is present, a constrain-free Berny optimization at ωB97x-D/3-21G reading the previously computed force constants is run (*opt=readfc,ts,noeigentest*) with a second hessian calculation.

3. If the frequency is still present, a second Berny optimization at ωB97x-D/6-31G(d) again reading the previously calculated force constants with a final hessian calculation.

In the second and third step described above, it became necessary to change the maximum displacement for each cycle of optimization from 0.30 Bohr (the default value) to between 0.05 Bohr and 0.01 Bohr (*maxstep=5* or *maxstep=1*). With some more tedious molecules a second partial optimization when rising the DFT level was crucial to ensure the maintenance of the correct negative normal mode. This workflow allowed to find most of the correct TS structures except those for E5 and F6. For these elusive TS, new guess geometries were obtained using the Growing String Method[21] (GSM) coupled with xTB. These guess geometries were then optimized according to the above protocol and all but one of the remaining TS could be located. As it was done for ground potentials structures, the same geometrical parameters are here below reported for all located transition states. TS's with BOC groups by the same side of the σ N-N bond are hereby called ***cis***, as opposed to ***trans*** ones that have BOC groups on opposite sides.

| CIS | A1 | | B2 | | C3 | | D4 | |
|---|---|---|---|---|---|---|---|---|
| | C | CC | C | CC | C | CC | C | CC |
| ϑ1 | 28.37 | -31.72 | 29.73 | -21.04 | 36.64 | -20.10 | 37.71 | -16.83 |
| ϑ2 | 35.29 | -14.39 | 16.00 | -31.08 | 24.62 | -25.20 | 22.18 | -34.56 |
| ϑ3 | 32.89 | -14.96 | 15.91 | -20.50 | 16.69 | -36.88 | 27.90 | -18.89 |
| ϑ4 | -67.13 | 59.15 | -56.56 | 62.69 | -81.94 | 38.19 | -67.56 | 65.54 |
| ϑ5 | 62.60 | 178.77 | 176.47 | -73.30 | -177.87 | -21.27 | 169.49 | -69.22 |
| ϑ6 | -4.33 | -63.54 | 63.03 | -175.06 | 65.39 | 175.99 | 63.31 | -174.20 |
| r1 | 3.54 | 4.47 | 4.53 | 4.60 | 4.38 | 3.99 | 4.50 | 4.57 |
| r2 | 2.89 | 3.11 | 3.14 | 3.59 | 3.10 | 3.58 | 3.06 | 3.61 |
| r3 | 3.41 | 3.61 | 3.60 | 3.17 | 3.59 | 2.86 | 3.60 | 3.14 |
| r4 | 1.44 | 1.43 | 1.43 | 1.43 | 1.44 | 1.44 | 1.45 | 1.43 |
| r5 | 1.38 | 1.45 | 1.44 | 1.38 | 1.46 | 1.39 | 1.46 | 1.37 |
| r6 | 1.45 | 1.37 | 1.37 | 1.45 | 1.39 | 1.41 | 1.38 | 1.45 |
| r7 | 1.47 | 1.48 | 1.47 | 1.46 | 1.49 | 1.48 | 1.48 | 1.46 |
| r8 | 1.47 | 1.46 | 1.46 | 1.48 | 1.48 | 1.49 | 1.47 | 1.48 |

| CIS | E5 | | F6 | | G7 | |
|---|---|---|---|---|---|---|
| | C | CC | C | CC | C | CC |
| ϑ1 | 25.41 | -22.29 | 27.69 | -25.77 | 17.26 | -18.53 |
| ϑ2 | 30.43 | -28.86 | 24.93 | -24.11 | 34.65 | -35.54 |
| ϑ3 | 32.89 | -39.54 | 28.61 | -25.02 | 13.49 | -23.57 |
| ϑ4 | -56.62 | 47.62 | -54.84 | 57.16 | -68.81 | 63.94 |
| ϑ5 | 61.83 | 132.05 | 52.46 | 122.03 | 64.98 | -75.51 |
| ϑ6 | 157.73 | 9.31 | 171.35 | 12.53 | 176.98 | -175.00 |
| r1 | 4.59 | 2.94 | 4.42 | 3.01 | 4.50 | 4.51 |
| r2 | 3.56 | 2.67 | 3.57 | 2.72 | 3.61 | 3.60 |
| r3 | 3.06 | 3.43 | 3.02 | 3.41 | 3.12 | 3.10 |
| r4 | 1.42 | 1.43 | 1.42 | 1.42 | 1.43 | 1.44 |
| r5 | 1.40 | 1.40 | 1.39 | 1.39 | 1.37 | 1.38 |
| r6 | 1.47 | 1.47 | 1.46 | 1.46 | 1.45 | 1.46 |
| r7 | 1.46 | 1.47 | 1.49 | 1.48 | 1.47 | 1.46 |

| | | | | | | |
|---|---|---|---|---|---|---|
| r8 | 1.42 | 1.41 | 1.40 | 1.40 | 1.48 | 1.42 |

| TRANS | A1 | | B2 | | C3 | | D4 | |
|---|---|---|---|---|---|---|---|---|
| | C | CC | C | CC | C | CC | C | CC |
| ϑ1 | -13.13 | 20.71 | -21.33 | 29.34 | 35.49 | 23.38 | 23.46 | 23.46 |
| ϑ2 | 22.91 | -29.53 | 28.85 | -30.50 | -20.30 | -32.94 | -34.37 | -34.37 |
| ϑ3 | -152.26 | -173.62 | -161.50 | -168.35 | 170.61 | 160.85 | -177.37 | -177.37 |
| ϑ4 | -173.32 | -161.53 | -177.87 | -166.94 | 144.39 | -176.39 | -158.83 | -158.83 |
| ϑ5 | 14.61 | 162.93 | -166.00 | -14.41 | -179.60 | 105.76 | 158.85 | 11.77 |
| ϑ6 | -179.86 | -1.71 | 13.97 | 177.06 | 39.47 | 169.34 | 170.40 | -172.14 |
| r1 | 4.70 | 4.87 | 5.11 | 5.03 | 4.99 | 5.40 | 5.74 | 5.75 |
| r2 | 3.60 | 2.73 | 2.90 | 3.54 | 2.82 | 3.62 | 3.57 | 3.57 |
| r3 | 3.49 | 3.55 | 3.52 | 2.82 | 3.55 | 3.19 | 3.53 | 3.53 |
| r4 | 1.43 | 1.44 | 1.44 | 1.44 | 1.44 | 1.46 | 1.45 | 1.44 |
| r5 | 1.39 | 1.38 | 1.41 | 1.38 | 1.38 | 1.44 | 1.40 | 1.39 |
| r6 | 1.41 | 1.40 | 1.41 | 1.42 | 1.47 | 1.39 | 1.40 | 1.43 |
| r7 | 1.48 | 1.47 | 1.47 | 1.49 | 1.49 | 1.48 | 1.48 | 1.49 |
| r8 | 1.48 | 1.50 | 1.49 | 1.49 | 1.47 | 1.49 | 1.50 | 1.48 |

| TRANS | E5 | | F6 | | G7 | |
|---|---|---|---|---|---|---|
| | C | CC | C | CC | C | CC |
| ϑ1 | -25.12 | -25.68 | -31.56 | ***N.F.*** | -25.83 | -31.60 |
| ϑ2 | 28.25 | 23.63 | 24.53 | ***N.F.*** | 24.48 | 19.85 |
| ϑ3 | 169.33 | 179.27 | -166.89 | ***N.F.*** | -168.33 | -176.93 |
| ϑ4 | 166.89 | -175.74 | -174.11 | ***N.F.*** | -168.21 | -160.46 |
| ϑ5 | -129.29 | 172.15 | -179.69 | ***N.F.*** | 179.10 | -2.07 |
| ϑ6 | -40.38 | 14.21 | -8.82 | ***N.F.*** | 169.96 | 169.36 |
| r1 | 4.72 | 4.80 | 4.75 | ***N.F.*** | 5.79 | 4.88 |
| r2 | 2.73 | 2.66 | 2.62 | ***N.F.*** | 3.58 | 3.57 |
| r3 | 3.43 | 3.56 | 3.56 | ***N.F.*** | 3.56 | 2.74 |
| r4 | 1.42 | 1.43 | 1.43 | ***N.F.*** | 1.44 | 1.44 |
| r5 | 1.40 | 1.46 | 1.41 | ***N.F.*** | 1.41 | 1.41 |
| r6 | 1.40 | 1.47 | 1.40 | ***N.F.*** | 1.39 | 1.40 |
| r7 | 1.40 | 1.48 | 1.49 | ***N.F.*** | 1.48 | 1.40 |
| r8 | 1.41 | 1.48 | 1.48 | ***N.F.*** | 1.48 | 1.41 |

*Table S8. Geometrical parameters of obtained TSs. N.F.: not found.*

From these parameters, a constant trend in the spacing of the carbonyl oxygen atoms (r1) of 4-5 Å is observed, except for some *cis* geometries of A1, E5 and F6. Similarly, there are N-N bond distances (r4) around 1.4 Å, slightly longer than the bonds in the minimal structures. Both nitrogen atoms also show strong opposite pyramidalization, as shown by dihedral angles ϑ1 and ϑ2. Compared with equilibrium structures, TS geometries exhibit a mixed s-*cis* and s-*trans* configuration for BOC fragments, evidenced by ϑ5 and ϑ6.

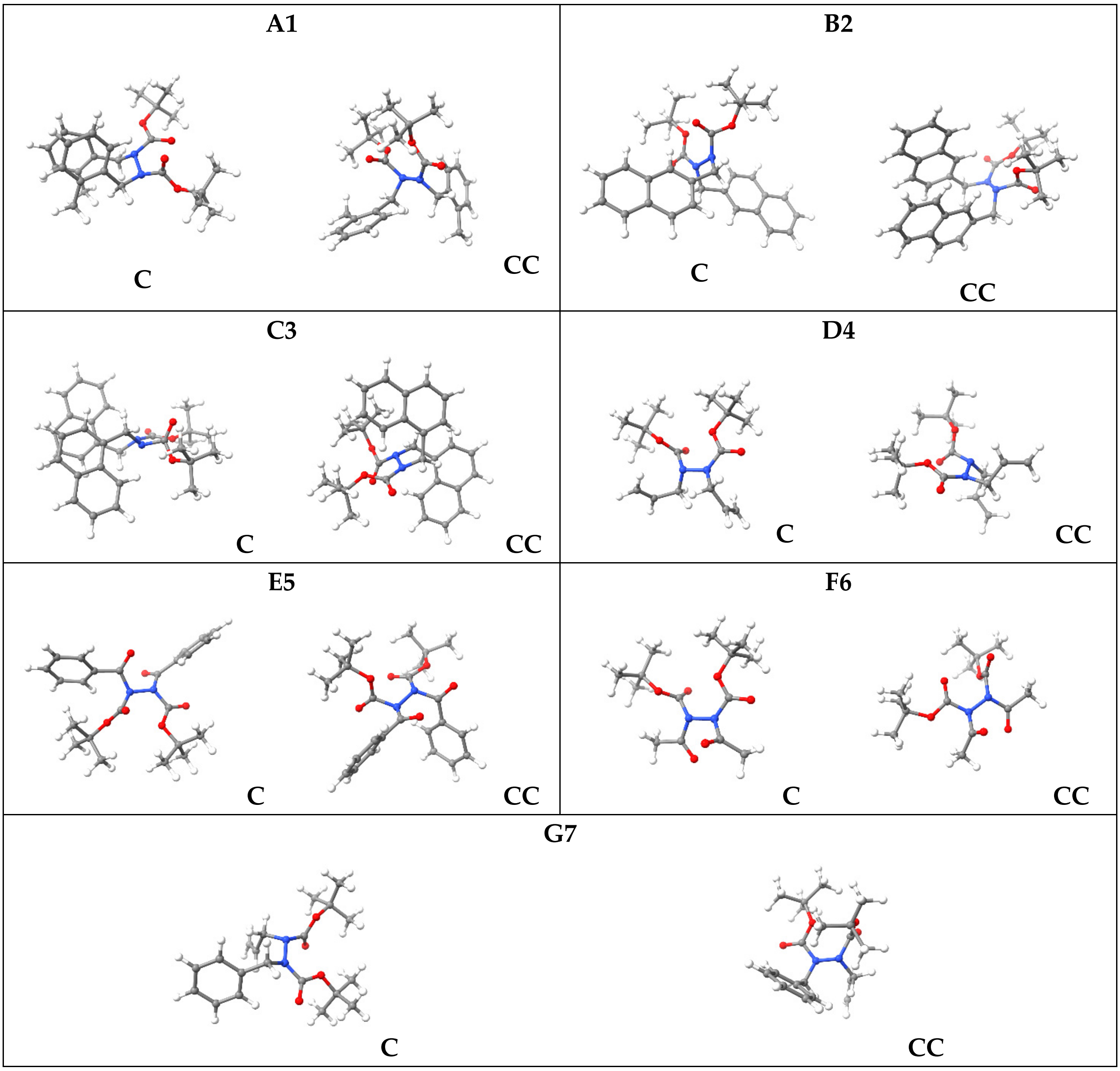


*Figure S12. Cis TS geometries.*

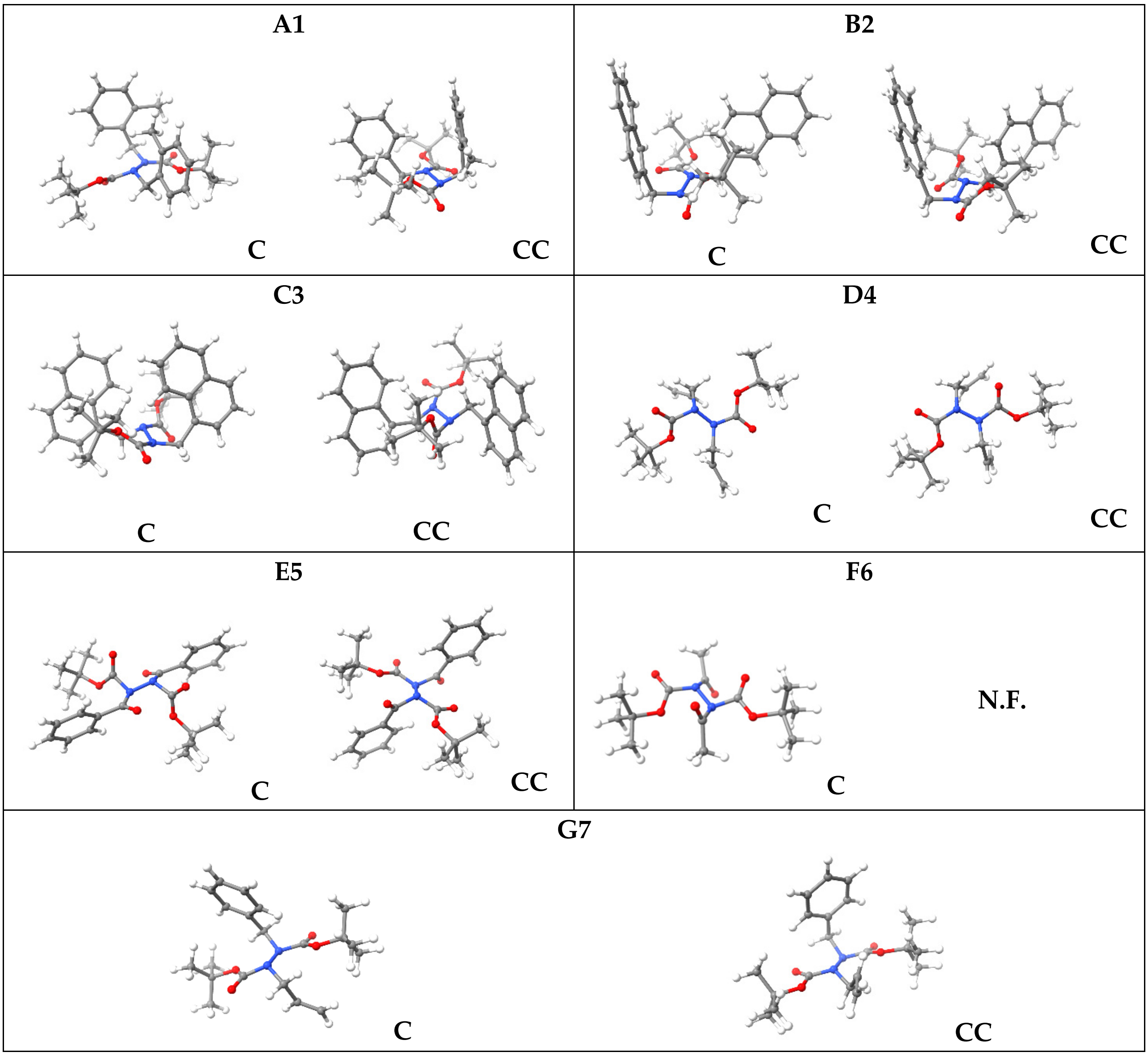


*Figure S13. Trans TS geometries. N.F.: not found.*

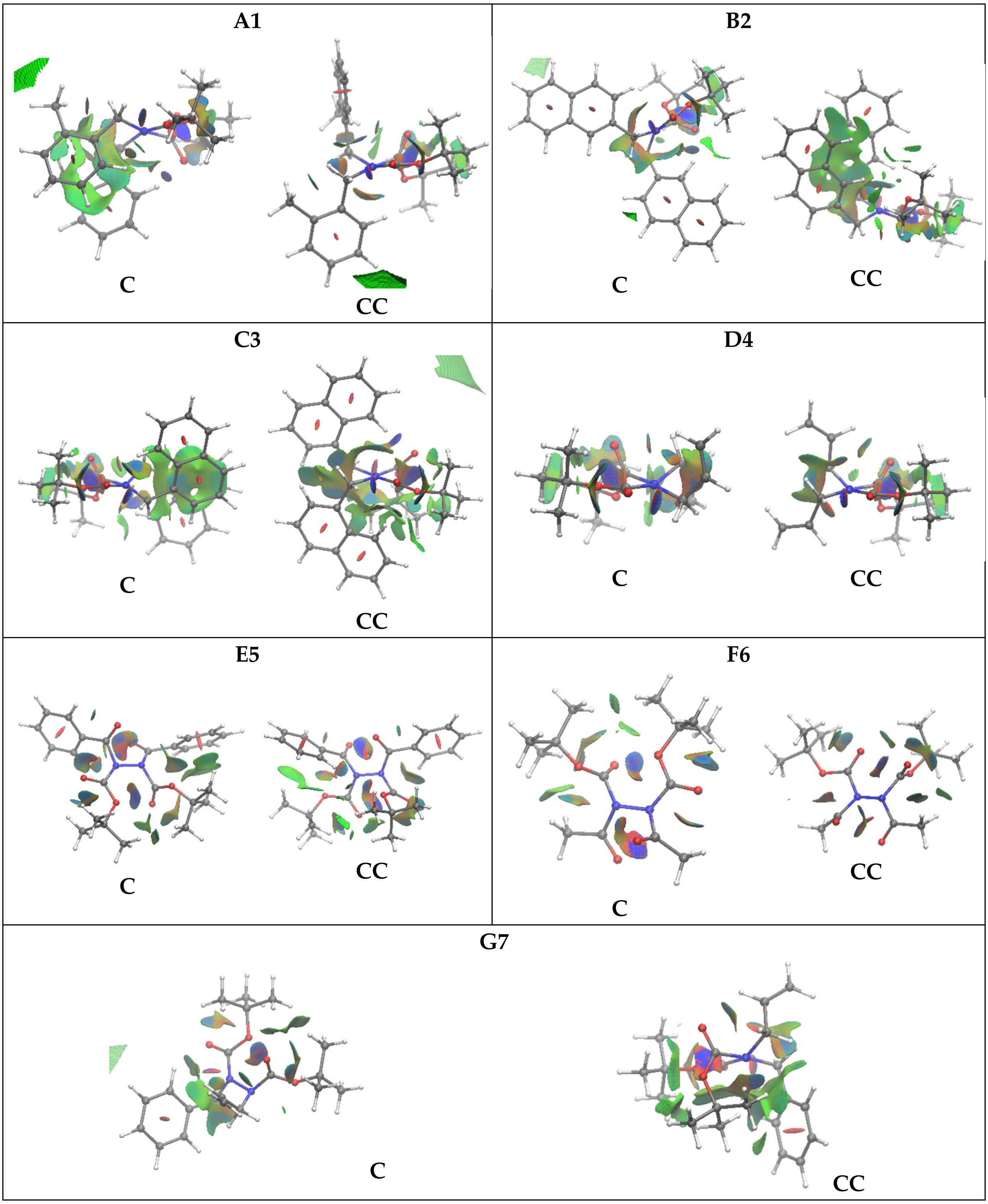


*Figure S14. NCI-index of cis TSs.*

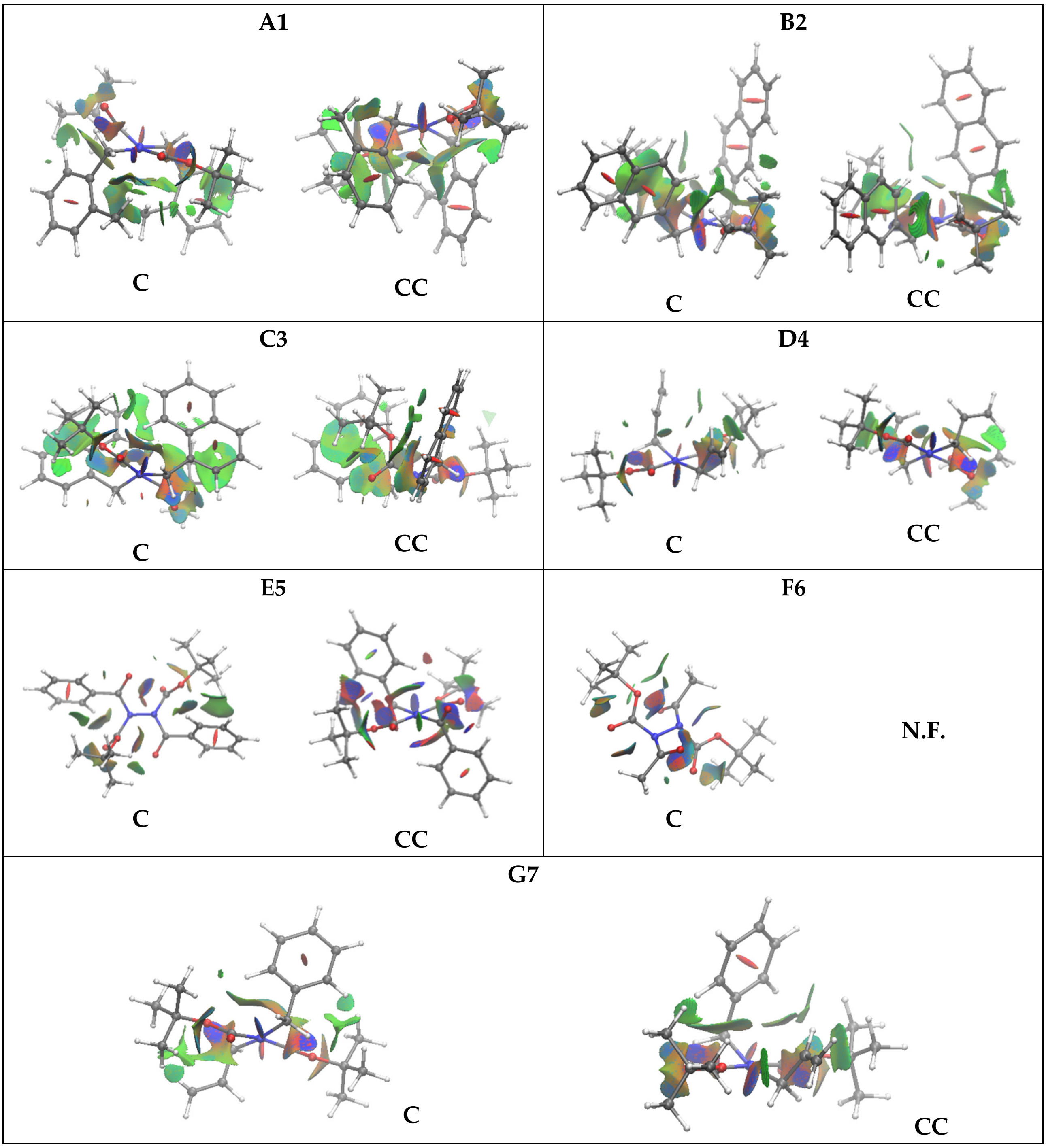


*Figure S15. NCI-index of trans TSs.*

From NCI surface analysis is possible to observe some important interactions: (i) π-π stacking between aromatic systems (for example in cis-**B2**-**CC**); (ii) methyl groups pointing towards a π system (as in trans-**C3**-**C**); (iii) carbonyl oxygen atoms interacting with hydrogen of opposite $CH_2$ groups (as can be seen in both trans-**G7** structures), (iv) the carbonyl oxygen of the BOCs can also give hydrogen bonds on its own *tert*-butyl (as for cis-**F6**-**c** TS), as shown by the blue attractive and weakly red repulsive zones between the oxygen and the carbons.

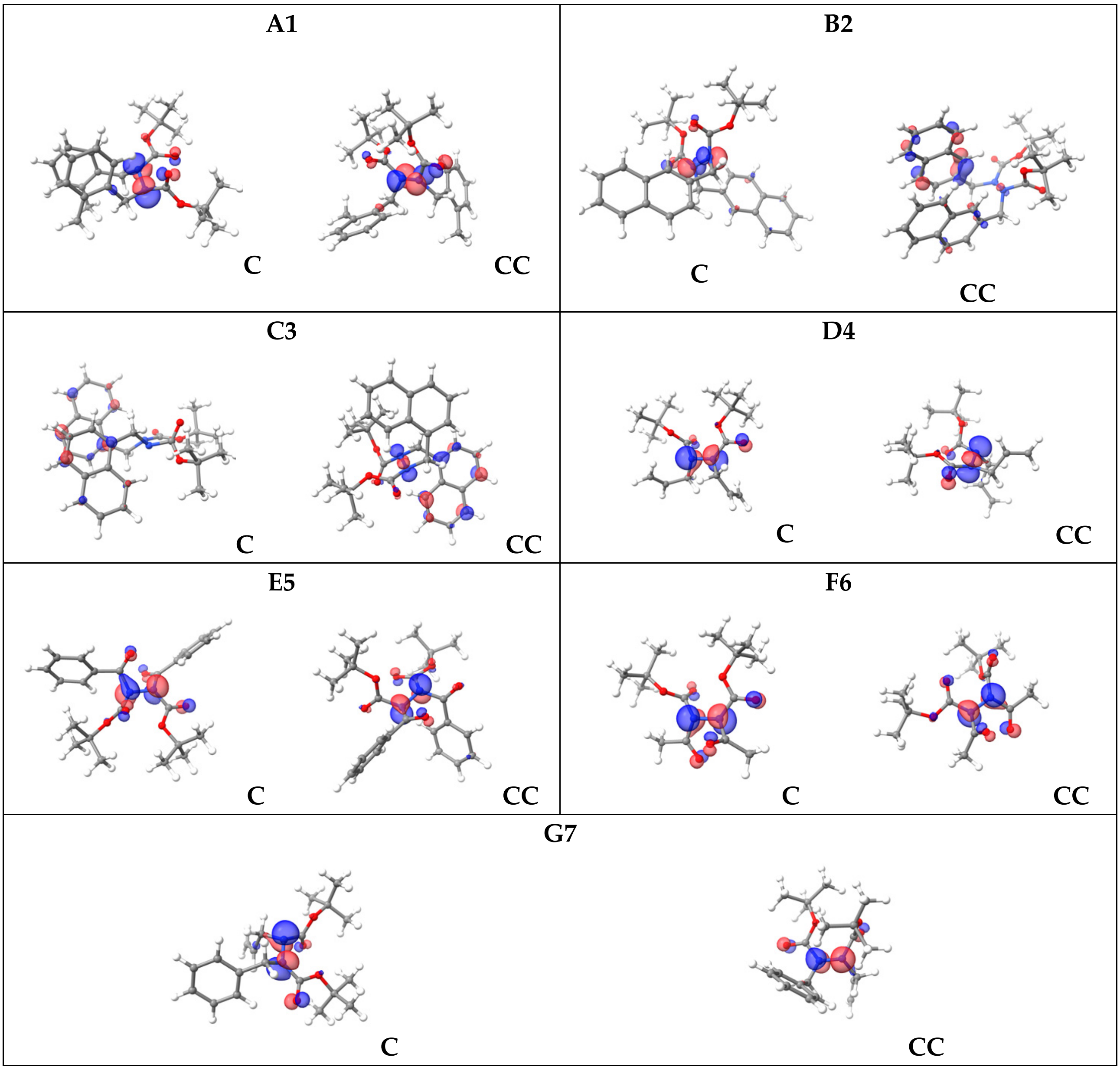


*Figure S16. HOMO orbital cis TSs.*

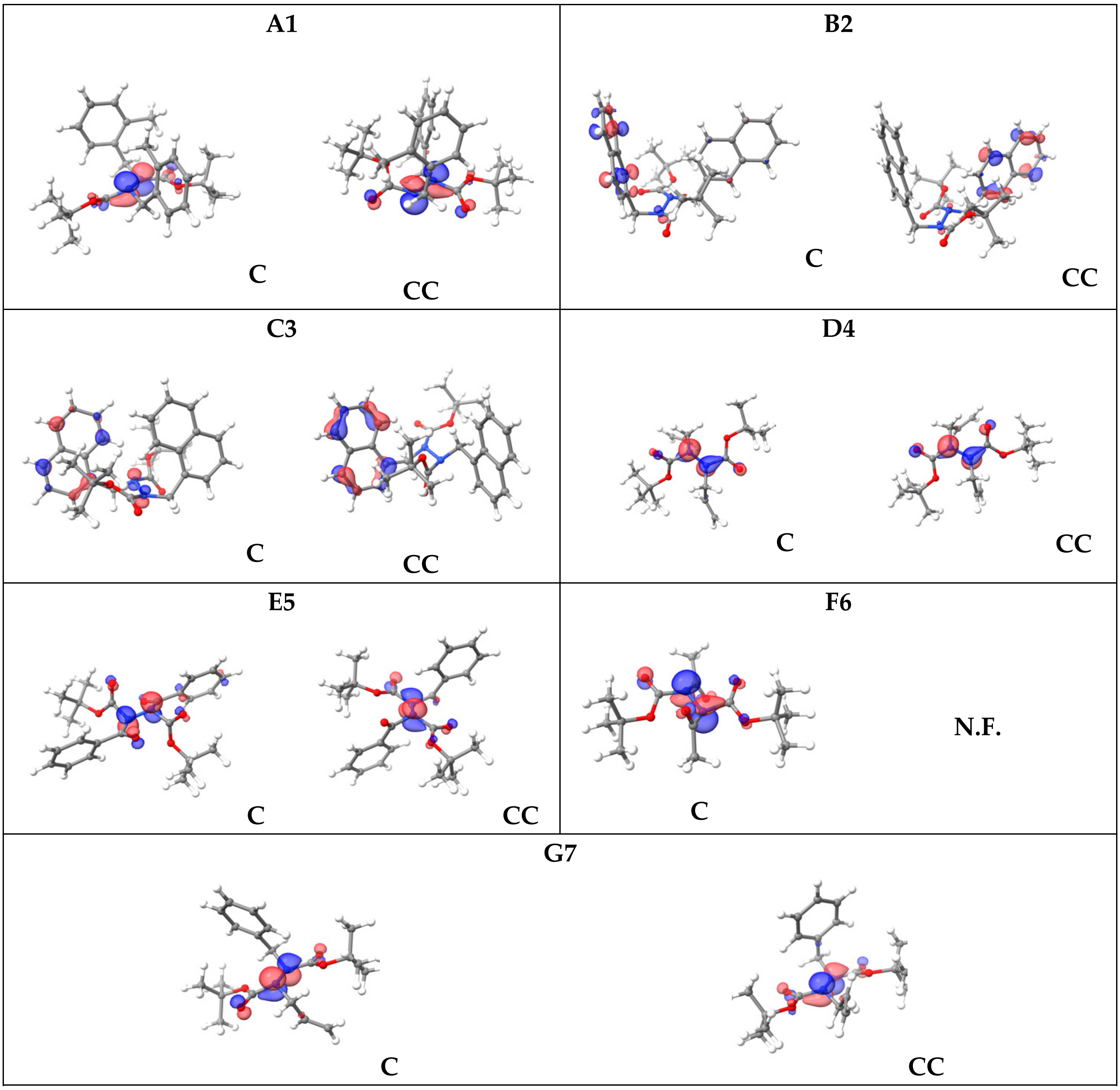


*Figure S17. HOMO orbital trans TSs.*

It can be seen from that the HOMO orbitals are located on the π system as in the case of A1, B2, and C3 structures. For the TSs of the other structures, these orbitals are centered on the carbonyls of the BOCs or on the two substituent groups and on the N-C bond as for F6 and D4. It is possible to observe a significant decrease in NCO conjugation probably due to the strong opposite pyramidalization of both nitrogen atoms. Normal mode vibrational analysis on the stationary points allowed to confirm minima (zero imaginary frequencies) or transition structures (TSs, one imaginary frequency). Zero-point energy, enthalpy and free energy corrections were obtained with a state of 1 atm pressure and 298.15 K temperature using Goodvibes[22] (version 3.2), with Grimme's quasiharmonic oscillator approximation at 298.15 K, with a frequency cut-off value of 100 $cm^{-1}$.[15] In the following tables, *GP1* and *GP2* names will describe respectively the favorited conformer (from CREST+CENSO protocol) and the conformer of the enantiomer derived from the scan.

| Clock | | | | | Counterclock | | | |
|---|---|---|---|---|---|---|---|---|
| ΔE | ΔH | ΔG | Im. Freq. | ***A1*** | ΔE | ΔH | ΔG | Im. Freq. |
| 0.00 | 0.00 | 0.00 | -- | *GP1* | 0.00 | 0.00 | 0.00 | -- |
| 7.31 | 7.24 | 6.15 | -- | *GP2* | 7.66 | 7.73 | 6.70 | -- |
| 34.05 | 34.08 | 35.70 | -18.28 | *Trans* | 31.39 | 30.76 | 31.66 | -22.38 |
| 36.67 | 35.51 | 36.37 | -43.38 | *Cis* | 42.00 | 41.31 | 41.88 | -43.38 |

| Clock | | | | | Counterclock | | | |
|---|---|---|---|---|---|---|---|---|
| ΔE | ΔH | ΔG | Im. Freq. | ***B2*** | ΔE | ΔH | ΔG | Im. Freq. |
| 0.00 | 0.00 | 0.00 | -- | *GP1* | 0.00 | 0.00 | 0.00 | -- |
| 1.57 | 1.28 | 1.32 | -- | *GP2* | 1.57 | 1.28 | 1.32 | -- |
| 28.01 | 27.42 | 29.24 | -12.73 | *Trans* | 29.37 | 28.71 | 29.81 | -17.05 |
| 37.91 | 37.01 | 37.68 | -39.72 | *Cis* | 33.50 | 32.21 | 33.63 | -39.72 |

| Clock | | | | | Counterclock | | | |
|---|---|---|---|---|---|---|---|---|
| ΔE | ΔH | ΔG | Im. Freq. | ***C3*** | ΔE | ΔH | ΔG | Im. Freq. |
| 0.00 | 0.00 | 0.00 | -- | *GP1* | 0.00 | 0.00 | 0.00 | -- |
| 2.28 | 2.09 | 1.43 | -- | *GP2* | 8.28 | 8.06 | 7.08 | -- |
| 37.79 | 36.85 | 38.02 | -7.17 | *Trans* | 40.36 | 39.40 | 39.83 | -20.25 |
| 43.20 | 41.86 | 42.25 | -32.57 | *Cis* | 45.78 | 45.27 | 47.09 | -32.57 |

| Clock | | | | | Counterclock | | | |
|---|---|---|---|---|---|---|---|---|
| ΔE | ΔH | ΔG | Im. Freq. | ***D4*** | ΔE | ΔH | ΔG | Im. Freq. |
| 0.00 | 0.00 | 0.00 | -- | *GP1* | 0.00 | 0.00 | 0.00 | -- |
| 2.61 | 2.21 | 1.87 | -- | *GP2* | 2.90 | 2.73 | 1.83 | -- |
| 31.03 | 30.26 | 31.40 | -40.72 | *Trans* | 27.48 | 26.52 | 27.60 | -25.76 |
| 32.86 | 31.93 | 33.82 | -37.83 | *Cis* | 31.79 | 30.80 | 32.07 | -37.83 |

| Clock | | | | | Counterclock | | | |
|---|---|---|---|---|---|---|---|---|
| ΔE | ΔH | ΔG | Im. Freq. | ***E5*** | ΔE | ΔH | ΔG | Im. Freq. |
| 0.00 | 0.00 | 0.00 | -- | *GP1* | 0.00 | 0.00 | 0.00 | -- |
| 2.68 | 2.55 | 2.65 | -- | *GP2* | 1.00 | 0.87 | 0.49 | -- |
| 24.07 | 23.01 | 24.36 | -18.92 | *Trans* | 26.11 | 24.89 | 25.93 | -18.14 |
| 27.91 | 26.82 | 28.32 | -25.83 | *Cis* | 25.31 | 24.13 | 24.94 | -25.83 |

| Clock | | | | | Counterclock | | | |
|---|---|---|---|---|---|---|---|---|
| ΔE | ΔH | ΔG | Im. Freq. | ***F6*** | ΔE | ΔH | ΔG | Im. Freq. |
| 0.00 | 0.00 | 0.00 | -- | *GP1* | 0.00 | 0.00 | 0.00 | -- |
| 5.98 | 5.90 | 5.72 | -- | *GP2* | 6.72 | 6.33 | 6.20 | -- |
| 30.33 | 29.04 | 30.10 | -14.99 | *Trans* | *N.F.* | *N.F.* | *N.F.* | *N.F.* |
| 32.28 | 31.30 | 32.71 | 32.28 | *Cis* | 27.30 | 26.06 | 26.82 | -40.9 |

| Clock | | | | | Counterclock | | | |
|---|---|---|---|---|---|---|---|---|
| ΔE | ΔH | ΔG | Im. Freq. | ***G7*** | ΔE | ΔH | ΔG | Im. Freq. |
| 0.00 | 0.00 | 0.00 | -- | *GP1* | 0.00 | 0.00 | 0.00 | -- |
| 3.25 | 3.17 | 2.34 | -- | *GP2* | 3.83 | 3.76 | 3.22 | -- |
| 30.07 | 29.51 | 30.95 | -24.61 | *Trans* | 28.99 | 28.42 | 29.87 | -24.36 |
| 34.70 | 33.67 | 34.62 | -52.57 | *Cis* | 30.32 | 29.44 | 31.35 | -52.57 |

*Table S9. Energy results. All energies are in kcal/mol.*

Table 8 summarizes all the minor energy values for the molecular rotational barrier at 298.15K and 1atm.

| | ΔE | ΔH | ΔG | Im. Freq. |
|---|---|---|---|---|
| ***A1*** | | | | |
| *GP1* | 0.00 | 0.00 | 0.00 | -- |
| *GP2* | 7.31 | 7.24 | 6.15 | -- |
| *Trans* | 31.39 | 30.76 | 31.66 | -22.38 |
| *Cis* | 36.67 | 35.51 | 36.37 | -43.38 |
| ***B2*** | | | | |
| *GP1* | 0.00 | 0.00 | 0.00 | -- |
| *GP2* | 1.57 | 1.28 | 1.32 | -- |
| *Trans* | 28.01 | 27.42 | 29.24 | -12.73 |
| *Cis* | 33.50 | 32.21 | 33.63 | -39.72 |
| ***C3*** | | | | |
| *GP1* | 0.00 | 0.00 | 0.00 | -- |
| *GP2* | 2.28 | 2.09 | 1.43 | -- |
| *Trans* | 37.79 | 36.85 | 38.02 | -7.17 |
| *Cis* | 43.20 | 41.86 | 42.25 | -32.57 |
| ***D4*** | | | | |
| *GP1* | 0.00 | 0.00 | 0.00 | -- |
| *GP2* | 2.90 | 2.73 | 1.83 | -- |
| *Trans* | 27.48 | 26.52 | 27.60 | -25.76 |
| *Cis* | 31.79 | 30.80 | 32.07 | -37.83 |
| ***E5*** | | | | |
| *GP1* | 0.00 | 0.00 | 0.00 | -- |
| *GP2* | 1.00 | 0.87 | 0.49 | -- |
| *Trans* | 24.07 | 23.01 | 24.36 | -18.92 |
| *Cis* | 25.31 | 24.13 | 24.94 | -25.83 |
| ***F6*** | | | | |
| *GP1* | 0.00 | 0.00 | 0.00 | -- |
| *GP2* | 5.98 | 5.90 | 5.72 | -- |
| *Trans* | 30.33 | 29.04 | 30.10 | -14.99 |
| *Cis* | 27.30 | 26.06 | 26.82 | -40.9 |
| ***G7*** | | | | |
| *GP1* | 0.00 | 0.00 | 0.00 | -- |
| *GP2* | 3.25 | 3.17 | 2.34 | -- |
| *Trans* | 28.99 | 28.42 | 29.87 | -24.36 |
| *Cis* | 30.32 | 29.44 | 31.35 | -52.57 |

*Table S10. Summary table of the lowest barrier for each molecule. N.A.: not available.*

As can be seen from the values above, the TS that determined the barrier is always the trans TS, as it has the lower energy value between the two TSs. To further improve the agreement of the

computational values for the racemization barrier with the experimental ones, the electronic energies of only GP1 and trans geometries for each molecule have been refined. As suggested by Goodman[23], this was conducted by modifying the functional and the basis set to the M06-2X/def2-TZVP level and performing a vertical energy calculation, but using the thermochemical correction previously obtained. Results are summarized in Table 9. To compare the computational data with the experimental data, all the computed ΔG of enantiomerization are recalculated to match the experimental temperature used for the kinetic experiments.

| Molecule | ΔG exp [kcal/mol] | T exp [°C] | ΔG computational [kcal/mol] | Deviation from experimental value [%] |
|---|---|---|---|---|
| A1 | < 24.3 | 70 | 29.23 | 20.29[a] |
| B2 | 25.09 | 71 | 26.31 | 4.87 |
| C3 | 26.01 | 71 | 36.23 | 39.29 |
| D4 | 25.57 | 70 | 24.81 | -2.96 |
| E5 | 20.61 | 25 | 22.57 | 9.52 |
| F6 | 23.10 | 40 | 28.46 | 23.20 |
| G7 | N.A. | | 26.87[b] | N.A. |

*Table S11. Single-Point refined rotational ΔG at M06-2x/def2-TZVP// ωB97x-D/6-31g(d) level. N.A.: not available. [a]: considering experimental ΔG=24.3 kcal/mol; [b]: calculated at 298.15 K.*

For most of the structures a good agreement with the experimental data was obtained. The deviation for the calculated epimerization barrier for the experimental value turned out to be particularly high for C3. Therefore, to locate a lower lying TS, a conformational search was performed on the calculated TS of C3. This study was conducted through CREST by imposing a force constant on the 6 atoms close to the stereogenic axis (the two nitrogen atoms and the four atoms bound to them) equal to 0.25 Bohr/Å$^2$ during the optimizations and excluding them from the meta-dynamics to generate the conformers' generation. The best conformer has then been used as the guess structure for the TS algorithm following the previously described protocol. The new TS geometry lowered the rotational ΔG to 36.09 kcal/mol at 298.15K. [8],[24],[25]

### *DIM Analysis*

To study the energetic contributions to the TSs energy an analysis was carried out employing the distortion-interaction model (DIM)[26],[27]. This was accomplished by dividing the molecule in 5 sections: the two BOC residues, the two substituent groups and the N-N fragment (Figure 14).

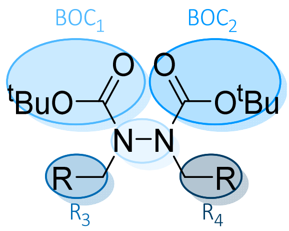


*Figure S18. Fragmentation of the molecule for DIM analysis.*

For each fragment, the distortion energy ($E_d^i$) was calculated as the difference of the energy of the segment at its TS geometry ($E_{TS_i}^*$) and at its GP1 geometry ($E_{GP_i}$). Then, the **interaction energy** ($E_i$)

was obtained by the difference between the single point energy of the whole TS and the sum of all distortion energies.

$$E_d^i = E_{TS_i}^* - E_{GP_i} \qquad E_i = E_{TS} - \sum^{\text{fragment}} E_d^i$$

The fragments were cut manually and then a dummy atom was used to cover the free valence.

<table>
<tr><th></th><th></th><th>Clock</th><th>Counterclock</th></tr>
<tr><td rowspan="2">A1</td><td>Cis</td><td>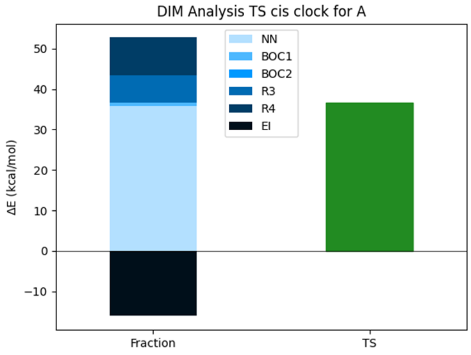
</td><td>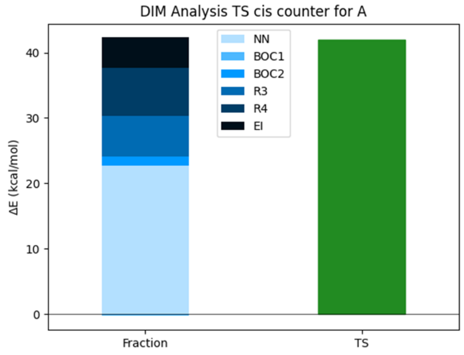
</td></tr>
<tr><td>Trans</td><td>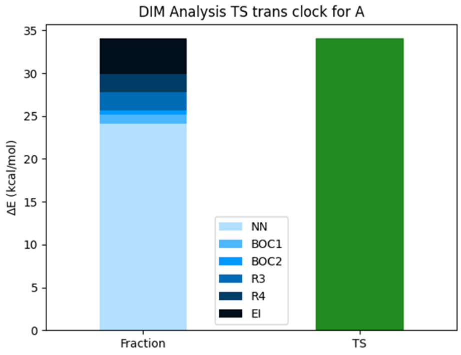
</td><td>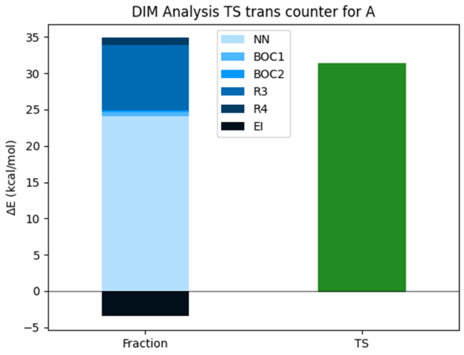
</td></tr>
<tr><td rowspan="2">B2</td><td>Cis</td><td>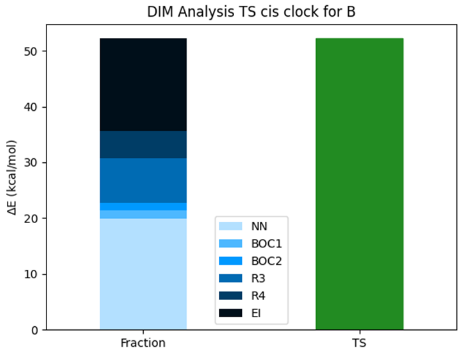
</td><td>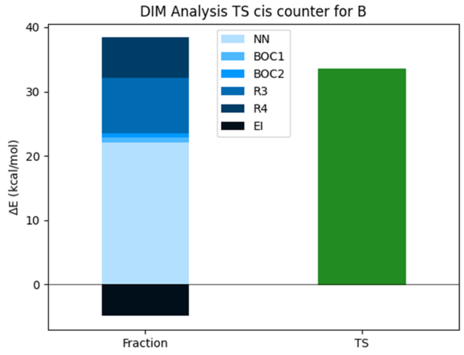
</td></tr>
<tr><td>Trans</td><td>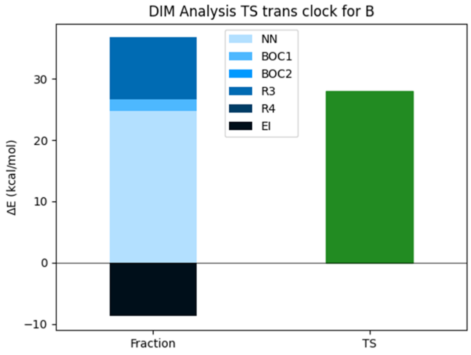
</td><td>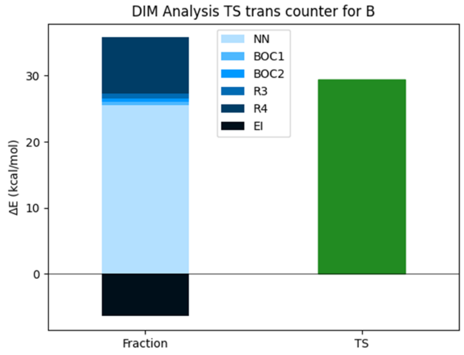
</td></tr>
</table>

<table>
<tr><td rowspan="2">C3</td><td>Cis</td><td>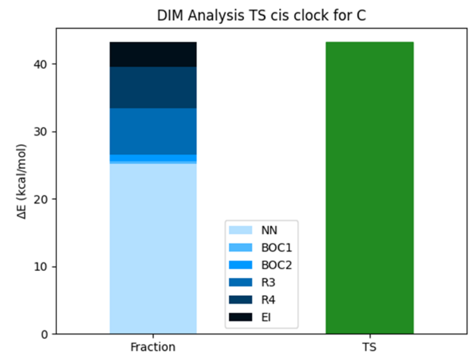
</td><td>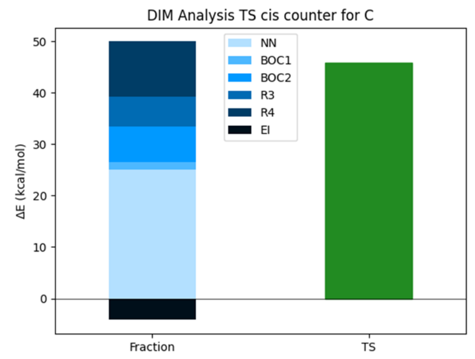
</td></tr>
<tr><td>Trans</td><td>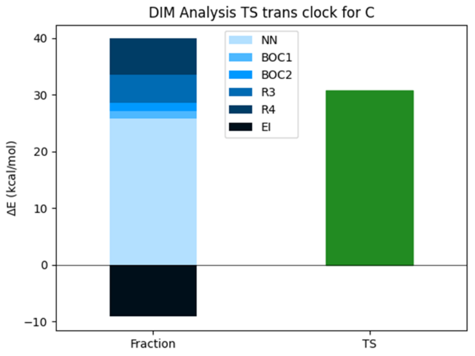
</td><td>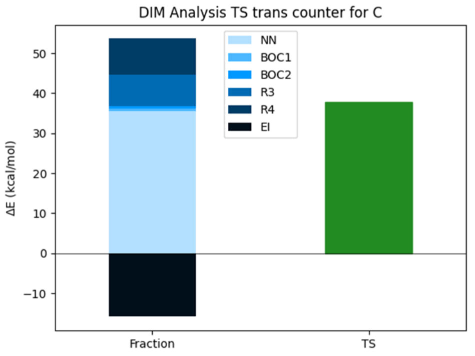
</td></tr>
<tr><td rowspan="2">D4</td><td>Cis</td><td>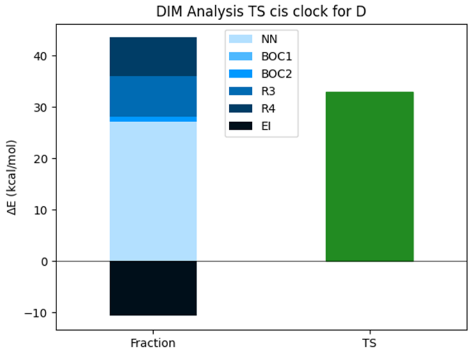
</td><td>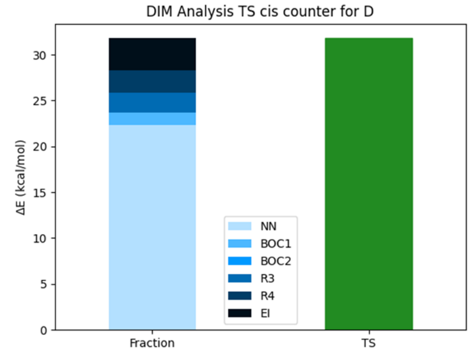
</td></tr>
<tr><td>Trans</td><td>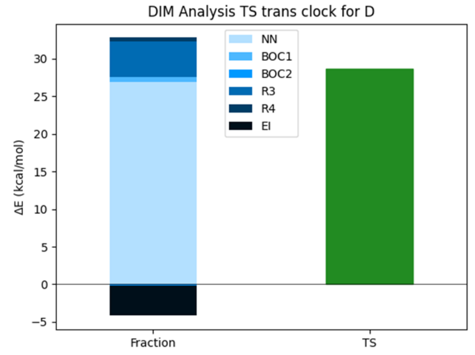
</td><td>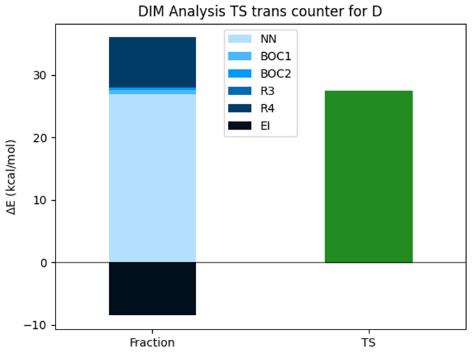
</td></tr>
</table>

<table>
<tr><td rowspan="2">**E5**</td><td>Cis</td><td>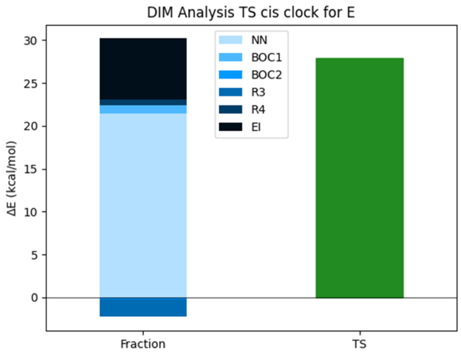</td><td>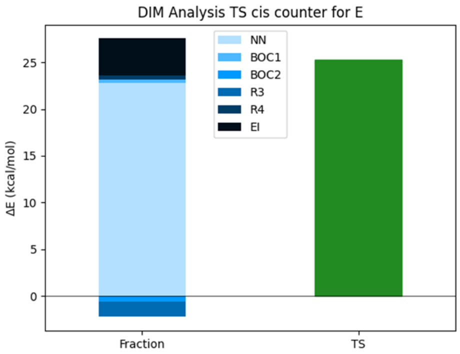</td></tr>
<tr><td>Trans</td><td>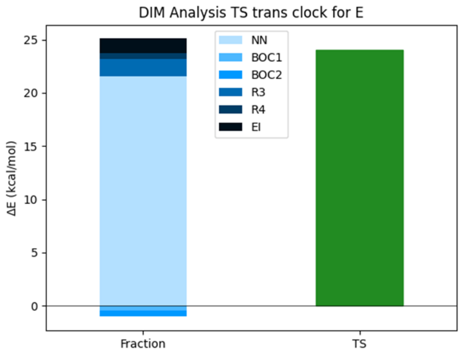</td><td>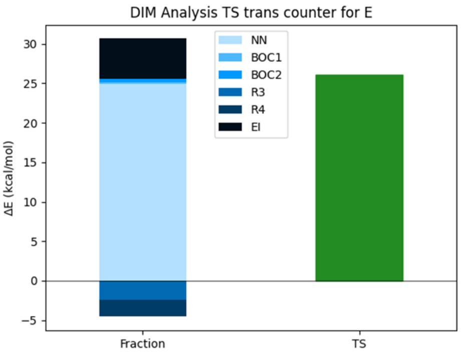</td></tr>
<tr><td rowspan="2">**F6**</td><td>Cis</td><td>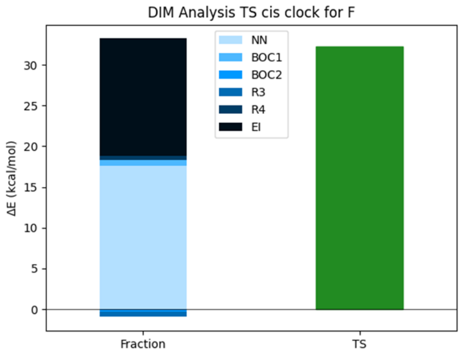</td><td>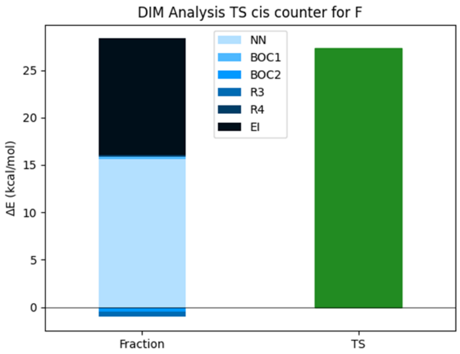</td></tr>
<tr><td>Trans</td><td>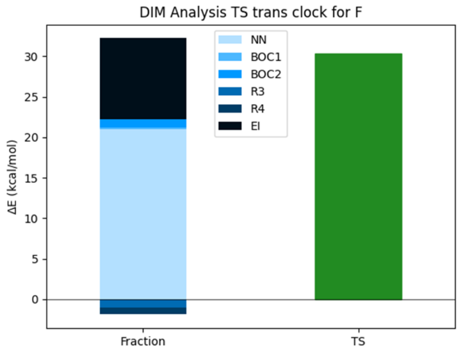</td><td>**N.F.**</td></tr>
</table>

<table>
<tr><td rowspan="2">G7</td><td>Cis</td><td>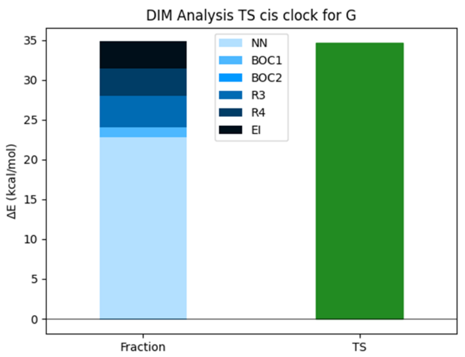
</td><td>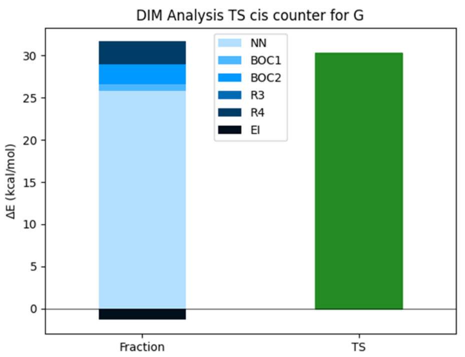
</td></tr>
<tr><td>Trans</td><td>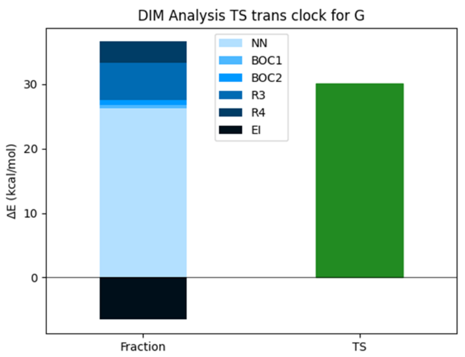
</td><td>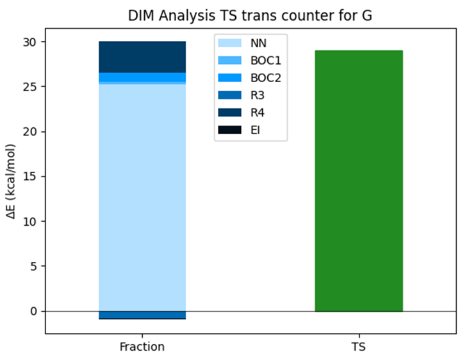
</td></tr>
</table>

*Table S12. DIM energies. N.F.: not found.*

From these graphs, it is notable that the major contribution to the distortion of the structure from the GP1 minimum state to TS is due to the N-N fragment, followed by the substituent groups $R_3$ and $R_4$, confirming that for both BOCs there is minimal distortion.

In addition, it is observed that for some structures such as in the cis clock and trans counter case of A1, the contribution of interaction energy is stabilizing. While in cases such as the cis clock of B there is for the interaction energy a destabilizing contribution. The magnitude of this contribution to the total TS energy does not have a constant trend. In the specific case of E5, the interaction energy of all four TS structures shows a significant destabilizing contribution.

## *Geometries*

**A1_GP2_C**

```
Inner Energy (Hartree)      : -1422.484942
Zero Point Energy (Hartree) : 0.590395
Enthalpy (Hartree)          : -1421.860464
Entropy (Hartree)           : 0.092613
Gibbs Energy (Hartree)      : -1421.953077
Minimum geometry
N  0.391281  -0.073629  -0.285055
N  0.604149  -1.277767  0.350810
C  -0.570670  -2.032115  0.768945
C  -0.124368  -0.117846  -1.649592
C  -1.631851  0.033170  -1.752688
C  -1.478557  -1.265664  1.706051
C  -2.332661  0.780030  -0.807038
C  -3.711309  0.940010  -0.898384
C  -4.403794  0.353790  -1.951160
C  -3.708475  -0.384435  -2.904751
C  -2.325034  -0.556003  -2.824623
C  -2.872163  -1.424252  1.630212
C  -3.675367  -0.689249  2.504855
C  -3.125640  0.187890  3.434275
C  -1.744207  0.331336  3.509496
C  -0.929393  -0.400048  2.651653
C  -1.604084  -1.366096  -3.875438
C  -3.515022  -2.340144  0.617385
O  0.677716  2.114905  -0.460189
C  1.066856  3.456292  -0.035943
C  0.393049  3.794730  1.292255
C  0.513355  4.328895  -1.158602
C  2.588442  3.563630  0.035240
C  0.952371  1.043984  0.292963
O  1.587630  1.034323  1.328691
O  2.686602  -1.134665  -0.434362
C  4.132631  -1.283114  -0.304556
C  4.535570  -1.141197  1.161314
C  4.655432  -0.103952  -1.120078
C  4.578590  -2.610853  -0.910710
C  1.847975  -1.874875  0.293106
O  2.098088  -2.929297  0.844974
H  -1.125679  -2.343800  -0.123917
H  -0.187745  -2.938487  1.243760
H  0.371585  0.662739  -2.232476
H  0.200351  -1.075512  -2.064801
H  -1.798455  1.219932  0.027960
H  -4.238232  1.509773  -0.139282
H  -5.480884  0.466583  -2.032322
H  -4.249664  -0.843162  -3.728670
H  -4.755305  -0.801506  2.446345
H  -3.774758  0.753878  4.096301
H  -1.298642  1.009537  4.231311
H  0.147933  -0.275290  2.689614
H  -2.295587  -1.692367  -4.656919
H  -0.804901  -0.790944  -4.356852
H  -1.142990  -2.264813  -3.447092
H  -4.600865  -2.355157  0.745700
H  -3.310205  -2.003974  -0.406537
H  -3.154551  -3.370997  0.708687
H  0.583375  4.844225  1.538713
H  -0.689759  3.651475  1.213112
H  0.775525  3.167980  2.099000
H  0.728536  5.381844  -0.953478
H  -0.570335  4.202123  -1.242008
H  0.970286  4.060047  -2.116032
H  3.032815  3.249372  -0.915067
H  2.871024  4.605529  0.217529
H  2.989537  2.943721  0.838365
H  4.201482  -1.998223  1.748975
H  5.625647  -1.071063  1.233091
H  4.094441  -0.230878  1.578885
H  5.749670  -0.102671  -1.118006
H  4.301912  0.839137  -0.691724
H  4.309113  -0.169673  -2.156321
H  4.232455  -2.689989  -1.946498
H  5.672322  -2.664091  -0.908860
H  4.182209  -3.451144  -0.338815
```

**A1_CIS_C**

```
Inner Energy (Hartree)      : -1422.438149
Zero Point Energy (Hartree) : 0.589761
Enthalpy (Hartree)          : -1421.815410
Entropy (Hartree)           : 0.089502
Gibbs Energy (Hartree)      : -1421.904912
Imaginary frequency (cm-1): -43.38
N  0.294985  0.596899  -0.438985
N  0.694441  -0.761814  -0.685106
C  -0.430599  -1.704411  -0.565786
C  -0.675036  1.058946  -1.448592
C  -2.123547  0.968119  -1.017866
C  -1.346358  -1.688266  0.650813
C  -2.469041  1.361127  0.274602
C  -3.783361  1.294376  0.715682
C  -4.774092  0.840622  -0.149526
C  -4.439162  0.471802  -1.447071
C  -3.119068  0.530350  -1.903857
C  -2.603387  -2.309237  0.525153
C  -3.478569  -2.292534  1.611406
C  -3.135436  -1.676128  2.809713
C  -1.890343  -1.072682  2.931746
C  -1.004443  -1.082151  1.858895
C  -2.801517  0.121675  -3.323880
C  -3.029401  -2.982135  -0.757210
O  0.795506  2.697983  0.078088
C  1.649900  3.830006  0.432253
C  2.241353  3.617909  1.823859
C  0.664213  4.994868  0.436018
C  2.724199  4.035831  -0.633282
C  1.297805  1.466147  -0.066008
O  2.453051  1.137280  0.134710
O  2.718820  -1.696306  -0.824409
C  3.968389  -2.327657  -0.392706
C  4.800191  -1.337621  0.418752
C  4.651045  -2.644792  -1.720120
C  3.666288  -3.611107  0.377897
C  1.847022  -1.238632  0.061395
O  1.924849  -1.340320  1.261527
H  -1.046666  -1.565776  -1.459651
H  0.022879  -2.696275  -0.686751
H  -0.434769  2.098302  -1.678778
H  -0.482240  0.477754  -2.354654
H  -1.683025  1.692429  0.945933
H  -4.029508  1.581025  1.733254
H  -5.805785  0.774450  0.183318
H  -5.215803  0.123484  -2.123809
H  -4.450755  -2.769227  1.510560
H  -3.835625  -1.672637  3.640266
H  -1.601408  -0.590987  3.861564
H  -0.033883  -0.616637  1.962923
H  -3.711725  -0.174215  -3.852794
H  -2.339104  0.937361  -3.890953
H  -2.110085  -0.729744  -3.363609
H  -3.982723  -3.500858  -0.623044
H  -3.165661  -2.250266  -1.562834
H  -2.295169  -3.719137  -1.102568
H  2.770805  4.524596  2.134022
H  1.443140  3.421897  2.546932
H  2.940573  2.780778  1.831638
H  1.182932  5.920282  0.703591
H  -0.134434  4.819508  1.163043
H  0.213093  5.121601  -0.552974
H  2.264287  4.119809  -1.623482
H  3.262974  4.966138  -0.426826
H  3.438948  3.211817  -0.641060
H  4.330904  -1.117449  1.378189
H  5.791574  -1.766219  0.598563
H  4.917146  -0.401901  -0.135071
H  5.613026  -3.132881  -1.536654
H  4.827900  -1.727091  -2.288916
H  4.029756  -3.314400  -2.322815
H  3.016966  -4.264773  -0.214092
H  4.602700  -4.144405  0.571065
H  3.182705  -3.397614  1.332100
```

**A1_GP2_CC**

Inner Energy (Hartree)      : -1422.484374
Zero Point Energy (Hartree) : 0.590589
Enthalpy (Hartree)          : -1421.859684
Entropy (Hartree)           : 0.092512
Gibbs Energy (Hartree)      : -1421.952196
Minimum geometry
N  0.693020  -0.523717  -0.005777
N  -0.293541  0.440037  -0.166633
C  -1.121566  0.332874  -1.380162
C  0.626406  -1.320883  1.219146
C  -0.622008  -2.164140  1.290682
C  -2.593061  0.603989  -1.143902
C  -1.543847  -1.921613  2.308009
C  -2.716402  -2.664088  2.411339
C  -2.974646  -3.660395  1.476834
C  -2.053342  -3.914023  0.463389
C  -0.868330  -3.184055  0.354163
C  -3.265947  1.674034  -1.756233
C  -4.631106  1.833790  -1.499224
C  -5.325070  0.965012  -0.663510
C  -4.655049  -0.100886  -0.071679
C  -3.299624  -0.274578  -0.321665
C  0.104782  -3.492549  -0.757595
C  -2.563115  2.642408  -2.675878
O  2.796685  -1.188293  -0.383453
C  4.092712  -1.208608  -1.059181
C  3.901568  -1.504028  -2.544765
C  4.806614  -2.365698  -0.366603
C  4.830814  0.105609  -0.814263
C  1.856354  -0.300942  -0.718364
O  1.956205  0.576273  -1.555320
O  0.949953  1.678475  1.223911
C  1.582455  2.902021  1.706098
C  2.173343  3.675173  0.529100
C  2.691674  2.362963  2.604885
C  0.582307  3.728688  2.509855
C  -0.015925  1.716125  0.301239
O  -0.620897  2.699539  -0.071364
H  -0.706234  0.982506  -2.153819
H  -0.996056  -0.699800  -1.712337
H  1.519461  -1.945186  1.238769
H  0.675845  -0.644489  2.079211
H  -1.345874  -1.124876  3.020383
H  -3.425081  -2.456431  3.207074
H  -3.888306  -4.244411  1.535161
H  -2.256063  -4.700843  -0.259011
H  -5.158831  2.662378  -1.965392
H  -6.385123  1.118623  -0.482172
H  -5.179661  -0.797706  0.575689
H  -2.774496  -1.110284  0.127569
H  -0.101397  -4.475439  -1.190407
H  0.040427  -2.752349  -1.562994
H  1.142645  -3.484201  -0.408764
H  -3.245147  3.435750  -2.995127
H  -1.709533  3.101437  -2.169512
H  -2.190537  2.145518  -3.579433
H  4.879731  -1.652430  -3.013325
H  3.316578  -2.420565  -2.674626
H  3.391775  -0.681647  -3.048386
H  5.808785  -2.490354  -0.787431
H  4.251994  -3.298807  -0.506637
H  4.901004  -2.172883  0.706453
H  4.889126  0.311174  0.259784
H  5.851152  0.025904  -1.202953
H  4.329034  0.937517  -1.310171
H  1.389399  4.096201  -0.102295
H  2.797082  4.491386  0.907373
H  2.795236  3.010640  -0.079029
H  3.253745  3.192379  3.044496
H  3.381695  1.739809  2.027150
H  2.270948  1.759108  3.415030
H  0.149930  3.123351  3.313210
H  1.096592  4.583150  2.961918
H  -0.221735  4.096481  1.870935

**A1_CIS_CC**
Inner Energy (Hartree)      : -1422.429650
Zero Point Energy (Hartree) : 0.590542
Enthalpy (Hartree)          : -1421.806161
Entropy (Hartree)           : 0.089972
Gibbs Energy (Hartree)      : -1421.896133
Imaginary frequency (cm-1): -38.33
N  0.256426  -0.219293  -0.313190
N  0.710051  0.059303  1.012591
C  2.032761  -0.491523  1.279394
C  0.179045  -1.667629  -0.600848
C  -0.824162  -2.516674  0.164638
C  3.093264  -0.089941  0.272758
C  -0.872989  -2.484288  1.562045
C  -1.769829  -3.273572  2.273382
C  -2.628989  -4.126241  1.590825
C  -2.577719  -4.177725  0.202704
C  -1.688468  -3.385234  -0.527321
C  4.230097  -0.896152  0.100985
C  5.216453  -0.482748  -0.797726
C  5.090853  0.701020  -1.516824
C  3.963154  1.495925  -1.339546
C  2.973388  1.097897  -0.448311
C  -1.682540  -3.493808  -2.034695
C  4.405458  -2.183866  0.870624
O  -0.639356  1.013108  -1.931849
C  -1.642639  1.398483  -2.928706
C  -2.368268  0.149165  -3.420336
C  -0.784281  2.000267  -4.037225
C  -2.607904  2.436928  -2.370453
C  -0.962612  0.382202  -0.808013
O  -2.073309  0.203438  -0.374716
O  -0.613101  1.871998  1.250451
C  -1.474028  2.726404  2.077659
C  -2.217601  1.863698  3.094748
C  -2.450440  3.314835  1.068156
C  -0.645720  3.831367  2.728707
C  0.280508  1.073601  1.833831
O  0.721375  1.209779  2.964070
H  2.297428  -0.138891  2.277706
H  1.960865  -1.581513  1.354653
H  -0.025840  -1.707054  -1.672899
H  1.189770  -2.074622  -0.494107
H  -0.217221  -1.817709  2.109466
H  -1.791748  -3.220535  3.357742
H  -3.333113  -4.750607  2.132896
H  -3.245743  -4.847812  -0.332832
H  6.099071  -1.103088  -0.933443
H  5.870551  0.999586  -2.211418
H  3.850126  2.422194  -1.894908
H  2.085465  1.709726  -0.324428
H  -2.411220  -4.237115  -2.369161
H  -1.945284  -2.542338  -2.509588
H  -0.703183  -3.795923  -2.422714
H  5.373721  -2.640222  0.647727
H  4.355413  -2.021263  1.953285
H  3.629981  -2.918099  0.618281
H  -3.046366  0.419393  -4.235707
H  -1.648407  -0.581214  -3.804974
H  -2.955679  -0.306893  -2.620134
H  -1.418904  2.313325  -4.871725
H  -0.059825  1.266964  -4.403483
H  -0.238289  2.873298  -3.667275
H  -2.055466  3.277699  -1.941929
H  -3.233101  2.816822  -3.184985
H  -3.253959  2.006309  -1.605231
H  -1.538229  1.459003  3.846772
H  -2.974870  2.471853  3.599615
H  -2.719935  1.037162  2.581990
H  -3.144595  3.989593  1.578196
H  -3.020173  2.519448  0.582933
H  -1.914198  3.884241  0.303323
H  -0.082521  4.378491  1.965585
H  -1.317671  4.536396  3.229463
H  0.052176  3.422386  3.459137

**A1_GP1**
Inner Energy (Hartree)      : -1422.496585
Zero Point Energy (Hartree) : 0.590922
Enthalpy (Hartree)          : -1421.871997
Entropy (Hartree)           : 0.090873

Gibbs Energy (Hartree)      : -1421.962870
Minimum geometry
N  -0.029172  -0.839063  -0.686007
N  0.029222  -0.839113  0.685890
C  0.894789  -1.831142  1.318463
C  -0.894754  -1.831036  -1.318660
C  -2.338574  -1.701019  -0.889747
C  2.338618  -1.701068  0.889598
C  -2.799482  -2.482349  0.168224
C  -4.109232  -2.375865  0.629068
C  -4.974231  -1.479722  0.013311
C  -4.520133  -0.696667  -1.046392
C  -3.206842  -0.784979  -1.508581
C  3.206841  -0.785044  1.508517
C  4.520155  -0.696693  1.046404
C  4.974321  -1.479700  -0.013307
C  4.109363  -2.375819  -0.629156
C  2.799587  -2.482335  -0.168393
C  -2.739682  0.097097  -2.637611
C  2.739606  0.096955  2.637579
O  1.358602  0.909520  -0.637618
C  2.165088  1.986705  -1.201067
C  3.268094  1.415142  -2.088838
C  2.767124  2.639717  0.039282
C  1.260585  2.969876  -1.941060
C  0.681569  0.078628  -1.428142
O  0.647714  0.097250  -2.646031
O  -1.358692  0.909373  0.637679
C  -2.165143  1.986543  1.201215
C  -3.268174  1.414944  2.088933
C  -2.767156  2.639664  -0.039086
C  -1.260630  2.969652  1.941279
C  -0.681579  0.078459  1.428113
O  -0.647732  0.096935  2.646005
H  0.766880  -1.699483  2.392623
H  0.524923  -2.831845  1.061967
H  -0.766860  -1.699293  -2.392810
H  -0.524872  -2.831752  -1.062243
H  -2.118445  -3.186053  0.641846
H  -4.447816  -2.989890  1.457855
H  -6.001328  -1.386281  0.353823
H  -5.201360  0.003299  -1.524400
H  5.201345  0.003265  1.524475
H  6.001436  -1.386234  -0.353757
H  4.447998  -2.989801  -1.457955
H  2.118584  -3.186024  -0.642085
H  -3.546585  0.751667  -2.979216
H  -1.899239  0.724472  -2.326717
H  -2.396835  -0.490166  -3.496837
H  3.546405  0.751698  2.979097
H  1.898992  0.724149  2.326780
H  2.396987  -0.490384  3.496847
H  3.963033  2.216541  -2.361082
H  3.823067  0.645671  -1.542404
H  2.857299  0.978922  -3.000057
H  3.358822  3.513220  -0.251205
H  3.418169  1.934096  0.562593
H  1.978670  2.964323  0.725646
H  0.467579  3.325400  -1.274184
H  1.848787  3.834292  -2.265615
H  0.806858  2.504044  -2.817120
H  -2.857403  0.978695  3.000149
H  -3.963134  2.216325  2.361180
H  -3.823118  0.645484  1.542453
H  -3.358819  3.513171  0.251461
H  -3.418230  1.934106  -0.562448
H  -1.978686  2.964284  -0.725425
H  -0.467632  3.325233  1.274424
H  -1.848832  3.834039  2.265912
H  -0.806896  2.503754  2.817299

**A1_TRANS_CC**
Inner Energy (Hartree)      : -1422.446556
Zero Point Energy (Hartree) : 0.590734
Enthalpy (Hartree)          : -1421.822974
Entropy (Hartree)           : 0.089448
Gibbs Energy (Hartree)      : -1421.912423
Imaginary frequency (cm-1): -22.38
N  0.633526  -0.187029  -1.018183
N  -0.425921  -0.918775  -1.659731
C  -0.502204  -2.365296  -1.265361
C  0.586980  1.284508  -0.981289
C  0.428520  1.872803  0.402761
C  -0.668181  -2.582236  0.227000
C  0.213286  1.061121  1.511873
C  0.052452  1.613165  2.778339
C  0.109527  2.993122  2.942884
C  0.333233  3.807244  1.836187
C  0.497544  3.266116  0.559841
C  -1.927127  -2.766502  0.829602
C  -1.994294  -2.846939  2.223562
C  -0.853915  -2.780540  3.017720
C  0.394549  -2.669590  2.416428
C  0.475545  -2.579667  1.031299
C  0.720502  4.169956  -0.627553
C  -3.190279  -2.978235  0.026942
O  2.772611  0.035152  -0.507463
C  4.186137  -0.311053  -0.417509
C  4.348533  -1.645667  0.307266
C  4.743698  0.831216  0.427623
C  4.822891  -0.319889  -1.805143
C  1.898870  -0.728099  -1.177179
O  2.172595  -1.754317  -1.770738
O  -1.952019  0.549632  -0.739455
C  -3.205597  1.316551  -0.696194
C  -3.391824  2.073969  -2.007917
C  -2.979578  2.282548  0.461715
C  -4.375215  0.386108  -0.387735
C  -1.729542  -0.371723  -1.668194
O  -2.554257  -0.799362  -2.450633
H  -1.342882  -2.755133  -1.836549
H  0.400633  -2.847508  -1.627294
H  1.516391  1.651708  -1.421315
H  -0.208591  1.629372  -1.642177
H  0.159677  -0.012789  1.373987
H  -0.119961  0.961660  3.630284
H  -0.016376  3.436730  3.926274
H  0.379920  4.886382  1.962191
H  -2.965111  -2.978928  2.694983
H  -0.941583  -2.840836  4.098629
H  1.299376  -2.651724  3.016617
H  1.450982  -2.515460  0.559261
H  0.725506  5.220881  -0.325857
H  1.676458  3.961458  -1.123642
H  -0.065020  4.045480  -1.383979
H  -4.072779  -2.902057  0.668543
H  -3.309490  -2.269050  -0.792780
H  -3.191239  -3.982809  -0.414169
H  5.409657  -1.823322  0.509388
H  3.817128  -1.619941  1.264688
H  3.963911  -2.471082  -0.293956
H  5.816369  0.688128  0.589056
H  4.242684  0.867281  1.399582
H  4.591220  1.790807  -0.076021
H  4.635174  0.632021  -2.312687
H  5.905800  -0.446492  -1.705573
H  4.428291  -1.132755  -2.416182
H  -3.547460  1.391304  -2.844902
H  -4.264647  2.728988  -1.923111
H  -2.517588  2.701359  -2.213545
H  -3.887185  2.871778  0.625145
H  -2.152275  2.964773  0.251599
H  -2.741573  1.736792  1.378910
H  -4.159146  -0.201834  0.509928
H  -5.269136  0.988498  -0.196692
H  -4.578868  -0.289830  -1.218736

**A1_TRANS_C**
Inner Energy (Hartree)      : -1422.442323
Zero Point Energy (Hartree) : 0.592162
Enthalpy (Hartree)          : -1421.817681
Entropy (Hartree)           : 0.088295
Gibbs Energy (Hartree)      : -1421.905976
Imaginary frequency (cm-1): -18.28
N  0.121947  0.788546  -1.367972
N  -0.488937  -0.347710  -0.746815

C 0.252664 -1.608906 -0.533231
C -0.691229 2.030951 -1.376814
C -1.224323 2.464184 -0.023884
C 1.005356 -1.883977 0.757884
C -2.539971 2.119442 0.304714
C -3.102611 2.463226 1.526887
C -2.346006 3.187179 2.443198
C -1.050148 3.563595 2.114003
C -0.467646 3.216576 0.890155
C 1.168695 -1.031784 1.857923
C 1.929129 -1.487318 2.942648
C 2.511605 -2.748153 2.963807
C 2.328513 -3.598525 1.878093
C 1.578476 -3.159914 0.796257
C 0.948886 3.677602 0.633594
C 0.583870 0.352488 1.915766
O 2.239623 -0.025476 -1.120643
C 3.701883 0.089152 -1.121645
C 4.225999 0.182734 -2.552419
C 4.147695 -1.217598 -0.473073
C 4.120947 1.275762 -0.256774
C 1.476038 0.965077 -1.579537
O 1.906034 1.973914 -2.117940
O -2.404911 -1.378754 -0.305620
C -3.722801 -1.950912 -0.571427
C -4.776753 -0.846251 -0.587804
C -3.926554 -2.870923 0.628948
C -3.700970 -2.754265 -1.869824
C -1.800555 -0.574404 -1.187271
O -2.321582 -0.082511 -2.166789
H -0.507984 -2.384589 -0.608895
H 0.930002 -1.762467 -1.373950
H -0.036341 2.782525 -1.807259
H -1.520983 1.889278 -2.065206
H -3.135317 1.576852 -0.423521
H -4.124489 2.176080 1.757101
H -2.765646 3.467548 3.404992
H -0.464388 4.143741 2.823689
H 2.062038 -0.824570 3.794388
H 3.096389 -3.064545 3.822423
H 2.767017 -4.591881 1.870975
H 1.443089 -3.820954 -0.058221
H 0.996692 4.772649 0.631749
H 1.613296 3.328907 1.432521
H 1.359918 3.325696 -0.313093
H 0.788961 0.822925 2.882021
H 1.019628 0.986787 1.140268
H -0.496504 0.353052 1.754561
H 5.319513 0.130082 -2.537453
H 3.852375 -0.658185 -3.145939
H 3.923210 1.116079 -3.027018
H 5.241222 -1.260501 -0.458224
H 3.774106 -2.077431 -1.037469
H 3.778067 -1.292240 0.553082
H 3.656475 1.197556 0.732267
H 5.207769 1.260067 -0.125835
H 3.835841 2.224216 -0.713550
H -4.648799 -0.191171 -1.450902
H -5.773751 -1.296301 -0.632434
H -4.709975 -0.250581 0.328501
H -4.900133 -3.365244 0.558998
H -3.891092 -2.298379 1.560542
H -3.146832 -3.638035 0.662845
H -2.901086 -3.501915 -1.840421
H -4.653892 -3.280459 -1.984519
H -3.553906 -2.108045 -2.736597

**B2_GP2_C**
Inner Energy (Hartree) : -1651.062243
Zero Point Energy (Hartree) : 0.628644
Enthalpy (Hartree) : -1650.397480
Entropy (Hartree) : 0.096335
Gibbs Energy (Hartree) : -1650.493815
Minimum geometry
N 1.364116 -0.085071 -0.073496
N 1.714542 -1.316394 -0.589564
C 0.893341 -1.828792 -1.681155
C 0.728813 -0.058663 1.243507
C -0.740186 0.315049 1.246258
C -0.510276 -2.098240 -1.202115
C -1.282168 1.195487 0.344018
C -2.654717 1.543945 0.385545
C -3.486645 0.973489 1.386597
C -2.908590 0.066588 2.310014
C -1.577723 -0.251213 2.240019
C -3.227332 2.442774 -0.553071
C -4.559616 2.761498 -0.495889
C -5.386644 2.195307 0.503951
C -4.861736 1.321324 1.420601
C -1.577835 -1.390565 -1.687217
C -2.886627 -1.580007 -1.170152
C -3.083535 -2.521752 -0.124976
C -1.965655 -3.258321 0.350765
C -0.718266 -3.052991 -0.171885
C -3.995095 -0.830897 -1.640168
C -5.240101 -1.005309 -1.093595
C -5.436187 -1.940855 -0.051296
C -4.382897 -2.682132 0.420521
O 1.735595 2.098315 0.079152
C 2.164779 3.422403 -0.361159
C 1.566962 3.736741 -1.730677
C 3.689338 3.501364 -0.359111
C 1.568502 4.332128 0.708625
C 2.004872 1.001490 -0.633667
O 2.687544 0.932210 -1.637180
O 3.609835 -1.071015 0.556344
C 5.050602 -1.168812 0.767490
C 5.784755 -0.869975 -0.537642
C 5.405083 -2.538099 1.340972
C 5.304878 -0.065651 1.790807
C 2.999652 -1.786550 -0.392418
O 3.451729 -2.746817 -0.984069
H 0.897539 -1.105193 -2.504286
H 1.382267 -2.741680 -2.026054
H 0.851531 -1.062790 1.654321
H 1.288195 0.627023 1.887685
H -0.663768 1.628811 -0.435420
H -3.541995 -0.388421 3.066936
H -1.155023 -0.962574 2.945304
H -2.588537 2.875113 -1.319853
H -4.986661 3.451001 -1.218303
H -6.440707 2.454625 0.538554
H -5.494714 0.876675 2.184337
H -1.426725 -0.640721 -2.461002
H -2.116100 -3.984512 1.145727
H 0.134595 -3.610046 0.208280
H -3.837665 -0.099997 -2.428499
H -6.077894 -0.413921 -1.449175
H -6.426293 -2.066660 0.376991
H -4.529575 -3.401361 1.222563
H 0.477086 3.630206 -1.701981
H 1.800161 4.772176 -1.998322
H 1.970902 3.075678 -2.498801
H 4.079475 3.220801 0.624809
H 4.000960 4.529487 -0.569920
H 4.117742 2.840928 -1.114601
H 1.811133 5.376123 0.488817
H 0.479972 4.222960 0.739129
H 1.970581 4.080739 1.694801
H 5.641527 -1.670742 -1.265024
H 5.414136 0.066307 -0.966708
H 6.855596 -0.765060 -0.336115
H 4.825281 -2.729875 2.249755
H 6.468519 -2.559873 1.600976
H 5.201878 -3.327885 0.616387
H 4.726549 -0.245967 2.702303
H 6.366951 -0.034622 2.051987
H 5.016397 0.907382 1.380846

**B2_CIS_C**
Inner Energy (Hartree) : -1651.004326
Zero Point Energy (Hartree) : 0.628506
Enthalpy (Hartree) : -1650.340546
Entropy (Hartree) : 0.095316
Gibbs Energy (Hartree) : -1650.435861
Imaginary frequency (cm-1): -39.72

N 0.164582 0.291308 0.439887
N -0.123293 0.005181 -0.928466
C 0.648858 -1.130060 -1.421955
C -0.067291 -0.877103 1.307726
C -1.333088 -1.683110 1.084956
C 2.129565 -1.020466 -1.132482
C -2.505774 -1.133429 0.628735
C -3.659111 -1.933539 0.424361
C -3.603807 -3.325993 0.707291
C -2.383041 -3.872733 1.181333
C -1.282116 -3.076426 1.355005
C -4.873679 -1.378462 -0.059535
C -5.977783 -2.168323 -0.250567
C -5.922024 -3.554635 0.032657
C -4.762973 -4.119121 0.499715
C 2.838231 -2.124861 -0.733485
C 4.238596 -2.063217 -0.503084
C 4.909991 -0.822661 -0.679925
C 4.152926 0.311040 -1.082296
C 2.806451 0.215647 -1.302724
C 4.986759 -3.198206 -0.095662
C 6.337655 -3.104758 0.124133
C 7.005886 -1.870087 -0.052301
C 6.307572 -0.756223 -0.444502
O 0.717411 2.108512 1.589434
C 0.586997 3.135563 2.629520
C 2.043209 3.466134 2.942410
C -0.107904 2.536220 3.849820
C -0.141736 4.361582 2.092232
C -0.336429 1.480843 1.086610
O -1.494429 1.766792 1.275816
O -0.840412 2.098272 -1.349080
C -1.692171 3.048790 -2.070091
C -3.051168 2.409763 -2.346781
C -0.991967 3.518525 -3.342999
C -1.838060 4.199484 -1.082235
C -0.603669 0.889685 -1.861476
O -0.761711 0.571615 -3.029240
H 0.247864 -2.059095 -1.002463
H 0.463465 -1.160092 -2.496260
H 0.800204 -1.540540 1.210124
H -0.031401 -0.498355 2.335295
H -2.562986 -0.069090 0.424981
H -2.329666 -4.937227 1.394685
H -0.349091 -3.514034 1.703496
H -4.910892 -0.314165 -0.277285
H -6.901219 -1.733838 -0.621608
H -6.803380 -4.169557 -0.123266
H -4.716078 -5.183340 0.716641
H 2.327213 -3.075449 -0.588868
H 4.662310 1.262768 -1.209549
H 2.239771 1.095441 -1.593725
H 4.470874 -4.145662 0.038478
H 6.900682 -3.979890 0.434534
H 8.075545 -1.808821 0.124353
H 6.817233 0.194211 -0.581076
H 2.548710 3.850994 2.051683
H 2.091649 4.226707 3.727455
H 2.575218 2.574002 3.285571
H -1.147574 2.283248 3.633845
H -0.090886 3.263529 4.667444
H 0.419571 1.636514 4.183805
H -0.095277 5.161262 2.838482
H 0.341175 4.718324 1.177697
H -1.187952 4.139968 1.880370
H -2.964032 1.577878 -3.047470
H -3.490971 2.048349 -1.411308
H -3.722944 3.160167 -2.775524
H 0.005350 3.901637 -3.103136
H -1.571146 4.330947 -3.794395
H -0.896007 2.705408 -4.062506
H -0.861242 4.635364 -0.853839
H -2.471980 4.978874 -1.515921
H -2.291453 3.847433 -0.152542

**B2_GP2_CC**
Inner Energy (Hartree) : -1651.062243
Zero Point Energy (Hartree) : 0.628644
Enthalpy (Hartree) : -1650.397480
Entropy (Hartree) : 0.096335
Gibbs Energy (Hartree) : -1650.493815
Minimum geometry
N 1.364116 -0.085071 -0.073496
N 1.714542 -1.316394 -0.589564
C 0.893341 -1.828792 -1.681155
C 0.728813 -0.058663 1.243507
C -0.740186 0.315049 1.246258
C -0.510276 -2.098240 -1.202115
C -1.282168 1.195487 0.344018
C -2.654717 1.543945 0.385545
C -3.486645 0.973489 1.386597
C -2.908590 0.066588 2.310014
C -1.577723 -0.251213 2.240019
C -3.227332 2.442774 -0.553071
C -4.559616 2.761498 -0.495889
C -5.386644 2.195307 0.503951
C -4.861737 1.321324 1.420601
C -1.577835 -1.390565 -1.687217
C -2.886627 -1.580007 -1.170152
C -3.083535 -2.521752 -0.124976
C -1.965655 -3.258321 0.350765
C -0.718266 -3.052991 -0.171885
C -3.995095 -0.830897 -1.640168
C -5.240101 -1.005309 -1.093595
C -5.436187 -1.940855 -0.051296
C -4.382897 -2.682132 0.420521
O 1.735595 2.098315 0.079152
C 2.164779 3.422403 -0.361159
C 1.566962 3.736741 -1.730677
C 3.689338 3.501364 -0.359111
C 1.568502 4.332128 0.708625
C 2.004872 1.001490 -0.633667
O 2.687544 0.932210 -1.637180
O 3.609835 -1.071015 0.556344
C 5.050602 -1.168812 0.767490
C 5.784755 -0.869975 -0.537642
C 5.405083 -2.538099 1.340972
C 5.304878 -0.065651 1.790807
C 2.999652 -1.786550 -0.392418
O 3.451729 -2.746817 -0.984069
H 0.897539 -1.105193 -2.504286
H 1.382267 -2.741680 -2.026054
H 0.851531 -1.062790 1.654321
H 1.288195 0.627023 1.887685
H -0.663768 1.628811 -0.435420
H -3.541995 -0.388421 3.066936
H -1.155023 -0.962574 2.945304
H -2.588537 2.875113 -1.319853
H -4.986661 3.451001 -1.218303
H -6.440707 2.454625 0.538554
H -5.494714 0.876675 2.184337
H -1.426725 -0.640721 -2.461002
H -2.116100 -3.984512 1.145727
H 0.134595 -3.610046 0.208280
H -3.837665 -0.099997 -2.428499
H -6.077894 -0.413921 -1.449175
H -6.426293 -2.066660 0.376991
H -4.529575 -3.401361 1.222563
H 0.477086 3.630206 -1.701981
H 1.800161 4.772176 -1.998322
H 1.970902 3.075678 -2.498801
H 4.079475 3.220801 0.624809
H 4.000960 4.529487 -0.569920
H 4.117742 2.840928 -1.114601
H 1.811133 5.376123 0.488817
H 0.479972 4.222960 0.739129
H 1.970581 4.080739 1.694801
H 5.641527 -1.670742 -1.265024
H 5.414136 0.066307 -0.966708
H 6.855596 -0.765059 -0.336115
H 4.825281 -2.729875 2.249755
H 6.468519 -2.559873 1.600976
H 5.201878 -3.327885 0.616387
H 4.726549 -0.245967 2.702303
H 6.366951 -0.034622 2.051987
H 5.016397 0.907382 1.380846

**B2_CIS_CC**
Inner Energy (Hartree) : -1651.011354
Zero Point Energy (Hartree) : 0.627994
Enthalpy (Hartree) : -1650.348197
Entropy (Hartree) : 0.094117
Gibbs Energy (Hartree) : -1650.442314
Imaginary frequency (cm-1): -46.22
N 1.509386 0.636210 -1.680230
N 1.130553 -0.603542 -1.071109
C 0.434720 -1.484928 -2.028915
C 0.353665 1.410144 -2.129821
C -0.666040 1.719576 -1.053730
C -1.070139 -1.409565 -1.862205
C -0.387566 1.617983 0.284495
C -1.397714 1.818886 1.259069
C -2.708266 2.175402 0.840574
C -2.963711 2.311143 -0.548332
C -1.974601 2.079456 -1.465357
C -1.147319 1.625349 2.643540
C -2.150856 1.780515 3.565639
C -3.453769 2.145452 3.148288
C -3.723249 2.339880 1.817874
C -1.615381 -1.335559 -0.604124
C -3.011922 -1.214646 -0.413254
C -3.865689 -1.185640 -1.550910
C -3.284111 -1.288043 -2.840248
C -1.924178 -1.394811 -2.990765
C -3.581170 -1.085346 0.881138
C -4.933889 -0.936851 1.037782
C -5.785360 -0.911079 -0.093964
C -5.264280 -1.032596 -1.356498
O 3.334279 0.762276 -0.364647
C 4.676158 1.246545 -0.022358
C 5.150724 0.221271 0.998509
C 4.587972 2.632546 0.611670
C 5.559902 1.220338 -1.266145
C 2.608338 1.376716 -1.298931
O 2.848142 2.469516 -1.781389
O 1.676312 -1.426555 0.936278
C 1.969219 -2.568351 1.810422
C 3.453797 -2.680933 2.135545
C 1.434345 -3.832829 1.140661
C 1.162267 -2.231180 3.060238
C 2.094984 -1.379093 -0.324940
O 2.997853 -2.018647 -0.805908
H 0.743185 -1.246567 -3.053866
H 0.769356 -2.511334 -1.847793
H 0.764262 2.325576 -2.557831
H -0.141928 0.874047 -2.945097
H 0.597167 1.308211 0.621463
H -3.968014 2.566099 -0.876104
H -2.196161 2.142277 -2.527935
H -0.145687 1.342503 2.958136
H -1.949723 1.624860 4.621563
H -4.239881 2.266438 3.887771
H -4.725349 2.605311 1.490954
H -0.958863 -1.305297 0.261474
H -3.931711 -1.265013 -3.713088
H -1.491425 -1.452994 -3.987096
H -2.920365 -1.074191 1.744290
H -5.356680 -0.821647 2.031155
H -6.855981 -0.790782 0.043549
H -5.915559 -1.008872 -2.226728
H 5.138795 -0.782625 0.566405
H 6.169592 0.459644 1.317956
H 4.499070 0.234393 1.876976
H 4.267756 3.380860 -0.113135
H 5.572488 2.910453 1.002329
H 3.879725 2.619750 1.446606
H 6.589449 1.460598 -0.982396
H 5.548708 0.221091 -1.712924
H 5.223129 1.948150 -2.006163
H 4.041268 -2.869816 1.236306
H 3.814812 -1.768069 2.615654
H 3.599939 -3.512278 2.833020
H 0.382709 -3.700643 0.864324
H 1.502926 -4.672808 1.838583
H 2.011277 -4.083022 0.247038
H 0.099802 -2.130367 2.817993
H 1.278333 -3.024109 3.805158
H 1.510038 -1.289722 3.496467

**B2_GP1**
Inner Energy (Hartree) : -1651.064743
Zero Point Energy (Hartree) : 0.629201
Enthalpy (Hartree) : -1650.399522
Entropy (Hartree) : 0.096390
Gibbs Energy (Hartree) : -1650.495912
Minimum geometry
N -0.016928 -0.548321 -1.116680
N 0.168987 0.815375 -1.196371
C 1.242786 1.278216 -2.074026
C -0.964678 -1.137474 -2.060663
C -2.405088 -0.824369 -1.733995
C 2.618194 1.083493 -1.484513
C -3.117072 -1.628554 -0.878447
C -4.448307 -1.309660 -0.509404
C -5.050804 -0.134225 -1.039387
C -4.301452 0.672866 -1.934920
C -3.016072 0.337211 -2.269704
C -5.189892 -2.113567 0.397111
C -6.464008 -1.763588 0.762353
C -7.063072 -0.593937 0.234627
C -6.373901 0.199065 -0.646134
C 3.328112 -0.065462 -1.726099
C 4.591529 -0.291690 -1.121185
C 5.133116 0.706071 -0.264052
C 4.389440 1.897060 -0.045921
C 3.163934 2.075913 -0.629095
C 5.323427 -1.490403 -1.330699
C 6.530757 -1.690994 -0.711893
C 7.069334 -0.698294 0.141271
C 6.387899 0.472198 0.356972
O 1.153991 -0.543716 0.792285
C 1.752918 -1.121828 1.993161
C 2.524524 0.053867 2.582447
C 2.709499 -2.250022 1.613969
C 0.656383 -1.583786 2.950144
C 0.402944 -1.278869 -0.021329
O 0.103242 -2.451398 0.120019
O -1.266513 1.053328 0.503544
C -2.005504 1.767962 1.541243
C -2.892832 2.839148 0.911965
C -1.032465 2.344846 2.566682
C -2.858536 0.667828 2.164214
C -0.401526 1.682893 -0.284875
O -0.119408 2.868107 -0.263936
H 1.148969 0.724127 -3.013263
H 1.050027 2.331307 -2.281015
H -0.706994 -0.758933 -3.055594
H -0.787005 -2.212572 -2.051071
H -2.646405 -2.511210 -0.452297
H -4.761668 1.567306 -2.347142
H -2.447898 0.968133 -2.948574
H -4.724751 -3.008297 0.802564
H -7.020235 -2.382569 1.459889
H -8.072791 -0.327756 0.532454
H -6.830143 1.098121 -1.052604
H 2.915918 -0.832744 -2.378484
H 4.803259 2.661100 0.607299
H 2.585399 2.974327 -0.435696
H 4.905325 -2.251411 -1.984652
H 7.079361 -2.613834 -0.874407
H 8.026202 -0.868933 0.625347
H 6.798170 1.236317 1.012299
H 1.854346 0.896156 2.779823
H 2.994881 -0.247378 3.523565
H 3.301918 0.386440 1.889978
H 2.170678 -3.100584 1.193890
H 3.248363 -2.580126 2.508132
H 3.443209 -1.892348 0.884871
H 1.111205 -1.915857 3.888959
H -0.023143 -0.755245 3.174333
H 0.082537 -2.408222 2.524784
H -2.295575 3.639030 0.471835

H -3.522322 2.391363 0.136694
H -3.544082 3.265469 1.682024
H -0.399469 1.550320 2.974468
H -1.597283 2.789540 3.392301
H -0.396994 3.111450 2.120881
H -2.228012 -0.148165 2.530555
H -3.429442 1.073683 3.005019
H -3.557985 0.260546 1.429972

**B2_TRANS_CC**
Inner Energy (Hartree) : -1651.017942
Zero Point Energy (Hartree) : 0.629255
Enthalpy (Hartree) : -1650.353771
Entropy (Hartree) : 0.094635
Gibbs Energy (Hartree) : -1650.448406
Imaginary frequency (cm-1): -17.05
N -1.170834 0.315227 2.306911
N -0.108459 -0.320827 1.570195
C 1.239814 0.249140 1.822710
C -2.465114 -0.418199 2.309480
C -2.956556 -0.736627 0.906553
C 2.071355 0.382597 0.566278
C -3.402220 0.271835 0.082341
C -3.799449 0.016744 -1.253421
C -3.752814 -1.316744 -1.746586
C -3.321817 -2.347697 -0.873300
C -2.935786 -2.066494 0.411990
C -4.240286 1.055886 -2.116518
C -4.608596 0.783053 -3.408171
C -4.559008 -0.544505 -3.900122
C -4.143201 -1.568304 -3.088805
C 3.439125 0.412248 0.665833
C 4.262215 0.548593 -0.483462
C 3.647655 0.644972 -1.762064
C 2.229583 0.604754 -1.838772
C 1.464646 0.479662 -0.711534
C 5.678961 0.579883 -0.397783
C 6.447043 0.702890 -1.527586
C 5.835175 0.799186 -2.800073
C 4.468356 0.770105 -2.912977
O -0.511222 2.177604 1.144240
C -0.525306 3.603293 0.795895
C 0.660165 3.732992 -0.154536
C -0.304108 4.452765 2.044905
C -1.830100 3.938244 0.077240
C -1.384832 1.674004 2.010292
O -2.291089 2.292460 2.536264
O 0.798930 -2.119476 0.702294
C 1.362784 -3.463674 0.658287
C 0.256872 -4.480085 0.385504
C 2.119552 -3.750678 1.952359
C 2.324974 -3.366515 -0.522741
C -0.037126 -1.722723 1.656508
O -0.600350 -2.443607 2.453673
H 1.107133 1.224945 2.283484
H 1.776208 -0.368355 2.554652
H -2.356476 -1.317191 2.906476
H -3.153310 0.255918 2.815662
H -3.460014 1.292556 0.452113
H -3.297832 -3.371182 -1.239190
H -2.598750 -2.865646 1.064602
H -4.279392 2.072621 -1.733323
H -4.941695 1.584351 -4.060939
H -4.854341 -0.747184 -4.925244
H -4.106330 -2.588404 -3.462591
H 3.918584 0.319996 1.639175
H 1.755667 0.670183 -2.815103
H 0.383067 0.434975 -0.772849
H 6.146668 0.504910 0.580829
H 7.529956 0.726509 -1.449881
H 6.453915 0.895844 -3.687202
H 3.994068 0.842896 -3.888501
H 0.514916 3.117821 -1.046198
H 0.768715 4.777451 -0.462386
H 1.586014 3.412255 0.332562
H -1.141830 4.369999 2.737875
H -0.192885 5.500772 1.749083
H 0.615116 4.144252 2.554187
H -1.770129 4.954092 -0.326313
H -1.984904 3.246180 -0.757506
H -2.682528 3.878869 0.755628
H -0.409114 -4.578522 1.244291
H -0.330266 -4.168940 -0.484656
H 0.703958 -5.456054 0.170333
H 2.874475 -2.976203 2.125656
H 2.631484 -4.714403 1.868044
H 1.443261 -3.790956 2.808560
H 3.067245 -2.581936 -0.345359
H 2.842112 -4.320424 -0.664199
H 1.780398 -3.122247 -1.439605

**B2_TRANS_C**
Inner Energy (Hartree) : -1651.020100
Zero Point Energy (Hartree) : 0.629558
Enthalpy (Hartree) : -1650.355827
Entropy (Hartree) : 0.093484
Gibbs Energy (Hartree) : -1650.449311
Imaginary frequency (cm-1): -12.73
N -0.113079 0.781193 -1.725594
N -1.083102 -0.011327 -2.433694
C -2.525048 0.363819 -2.316805
C 1.277670 0.353355 -1.950861
C 2.045405 0.047357 -0.680797
C -3.109086 0.019960 -0.958696
C 3.342727 -0.388794 -0.779375
C 4.116523 -0.679301 0.374524
C 3.531834 -0.501335 1.658766
C 2.189782 -0.045190 1.735151
C 1.467556 0.215163 0.601806
C 5.456408 -1.142168 0.288233
C 6.177855 -1.414586 1.422101
C 5.595308 -1.237518 2.700295
C 4.303704 -0.791331 2.814310
C -2.942064 0.841346 0.131957
C -3.400894 0.464367 1.419328
C -4.063718 -0.782905 1.584188
C -4.251522 -1.605487 0.443619
C -3.786219 -1.217828 -0.785959
C -3.207947 1.297308 2.553957
C -3.649970 0.907419 3.791561
C -4.310479 -0.334928 3.955066
C -4.513199 -1.158566 2.877848
O 0.853412 2.653067 -1.083527
C 0.908731 3.934026 -0.386973
C -0.048042 3.924056 0.804186
C 0.618428 5.074651 -1.358261
C 2.357339 3.974301 0.092602
C -0.301887 2.133197 -1.520158
O -1.331230 2.770122 -1.663962
O -0.236612 -1.758061 -1.183367
C 0.034486 -3.174891 -0.888982
C -1.273861 -3.909826 -0.606201
C 0.888656 -3.118185 0.372577
C 0.822151 -3.785316 -2.044501
C -0.981881 -1.412989 -2.223704
O -1.577273 -2.168463 -2.963087
H -2.624519 1.415732 -2.552145
H -3.010339 -0.220084 -3.098509
H 1.256783 -0.524357 -2.596035
H 1.804009 1.132676 -2.508013
H 3.801265 -0.520815 -1.758316
H 1.732879 0.081483 2.713449
H 0.431368 0.527009 0.672891
H 5.901937 -1.277357 -0.694148
H 7.201561 -1.768352 1.343946
H 6.177194 -1.457556 3.590278
H 3.851590 -0.654511 3.793432
H -2.453614 1.803011 0.012436
H -4.768389 -2.554949 0.558236
H -3.923415 -1.867415 -1.645417
H -2.700482 2.249844 2.422043
H -3.496395 1.550187 4.653246
H -4.656652 -0.631902 4.940586
H -5.020369 -2.112267 3.000255
H 0.132271 3.039804 1.424939
H 0.129450 4.813787 1.416490

H -1.090243 3.930224 0.479442
H 1.290607 5.016035 -2.220239
H 0.787848 6.032505 -0.855478
H -0.413596 5.036723 -1.709639
H 2.544168 4.902879 0.640478
H 2.563693 3.125068 0.751263
H 3.043384 3.927818 -0.758629
H -1.843757 -3.378169 0.162172
H -1.887468 -3.996618 -1.502872
H -1.043090 -4.913461 -0.234572
H 1.837131 -2.608737 0.185495
H 1.100206 -4.137808 0.709271
H 0.361773 -2.588292 1.170790
H 1.731293 -3.202253 -2.227981
H 1.120327 -4.804640 -1.779512
H 0.226000 -3.822703 -2.957890

**C3_GP2_C**
Inner Energy (Hartree) : -1651.065527
Zero Point Energy (Hartree) : 0.629674
Enthalpy (Hartree) : -1650.400030
Entropy (Hartree) : 0.095356
Gibbs Energy (Hartree) : -1650.495386
Minimum geometry
N 0.901868 0.515003 -0.464272
N 0.901870 -0.515351 0.463940
C 0.292097 -0.224960 1.764192
C 0.291990 0.224664 -1.764485
C -1.201147 0.034479 -1.723867
C -1.201031 -0.034682 1.723669
C -1.722226 -1.218504 -1.933034
C -3.116305 -1.443782 -1.958660
C -3.978728 -0.398543 -1.773714
C -3.487108 0.913735 -1.547450
C -2.082562 1.140383 -1.517864
C -2.082528 -1.140533 1.517744
C -3.487060 -0.913813 1.547498
C -3.978586 0.398491 1.773805
C -3.116087 1.443678 1.958685
C -1.722023 1.218325 1.932914
C -4.370597 -2.005543 1.344686
C -4.370564 2.005510 -1.344524
C -3.888854 3.268366 -1.118527
C -2.493930 3.495586 -1.089783
C -1.613573 2.461583 -1.285613
C -1.613636 -2.461754 1.285420
C -2.494071 -3.495701 1.089653
C -3.888980 -3.268414 1.118590
O 2.612792 1.340061 0.716175
C 3.698511 2.259392 1.044817
C 4.233816 1.677427 2.349717
C 3.142886 3.664337 1.262603
C 4.767593 2.215884 -0.044437
C 1.884152 1.487195 -0.391165
O 2.007931 2.363384 -1.227397
O 2.612843 -1.340353 -0.716479
C 3.699269 -2.259038 -1.044587
C 3.144662 -3.664439 -1.262013
C 4.768099 -2.214419 0.044871
C 4.234446 -1.677127 -2.349562
C 1.884175 -1.487530 0.390833
O 2.008200 -2.363497 1.227256
H 0.565752 -1.057478 2.413705
H 0.766174 0.675751 2.168458
H 0.565644 1.057170 -2.414012
H 0.765984 -0.676074 -2.168783
H -1.047160 -2.058337 -2.076533
H -3.494372 -2.449792 -2.108801
H -5.053596 -0.560434 -1.782063
H -5.053445 0.560435 1.782292
H -3.494090 2.449704 2.108877
H -1.046896 2.058109 2.076427
H -5.441565 -1.819334 1.368019
H -5.441545 1.819355 -1.367716
H -4.574162 4.096454 -0.962898
H -2.116856 4.498802 -0.913309
H -0.546145 2.657519 -1.259636
H -0.546222 -2.657746 1.259319
H -2.117071 -4.498933 0.913111
H -4.574349 -4.096465 0.963027
H 4.575817 0.648746 2.199174
H 5.075428 2.277284 2.708749
H 3.454503 1.674391 3.118032
H 3.939073 4.315818 1.636784
H 2.340561 3.641242 2.007495
H 2.753873 4.082436 0.333046
H 4.394329 2.629525 -0.982125
H 5.637349 2.797029 0.278097
H 5.089975 1.183286 -0.214781
H 3.941378 -4.315503 -1.635792
H 2.755728 -4.082474 -0.332394
H 2.342484 -3.642153 -2.007087
H 4.394936 -2.628001 0.982626
H 5.089718 -1.181535 0.214921
H 5.638326 -2.795067 -0.277285
H 5.076540 -2.276524 -2.708233
H 3.455289 -1.674868 -3.118038
H 4.575711 -0.648165 -2.199267

**C3_CIS_C**
Inner Energy (Hartree) : -1651.000315
Zero Point Energy (Hartree) : 0.628665
Enthalpy (Hartree) : -1650.336649
Entropy (Hartree) : 0.093697
Gibbs Energy (Hartree) : -1650.430346
Imaginary frequency (cm-1): -32.57
N 0.771509 0.249985 -0.079590
N 0.534190 -1.024879 0.535205
C -0.552475 -0.968435 1.533797
C -0.197192 0.505639 -1.182652
C -1.492796 1.215645 -0.851607
C -1.985892 -1.160419 1.080851
C -2.618267 0.783517 -1.512723
C -3.857832 1.443454 -1.382653
C -3.965689 2.540691 -0.573678
C -2.831584 3.032442 0.122948
C -1.570124 2.380752 -0.022204
C -2.410454 -2.187419 0.179609
C -3.795277 -2.271686 -0.156065
C -4.725317 -1.394126 0.457952
C -4.300567 -0.456125 1.358379
C -2.926864 -0.335116 1.651652
C -4.227816 -3.247076 -1.093126
C -2.932622 4.181831 0.950852
C -1.836076 4.683968 1.601010
C -0.581880 4.052072 1.447198
C -0.450626 2.933720 0.662226
C -1.522319 -3.133432 -0.404556
C -1.974896 -4.068651 -1.300836
C -3.339583 -4.120251 -1.663718
O 2.559209 1.596734 -0.136061
C 3.461497 2.501978 -0.861118
C 3.595249 3.669470 0.111202
C 2.771577 2.945752 -2.148920
C 4.814205 1.853827 -1.126820
C 2.069513 0.495890 -0.693262
O 2.508834 -0.078430 -1.658534
O 2.767338 -1.440420 0.666593
C 3.926768 -2.343876 0.670096
C 3.715708 -3.447309 -0.362723
C 4.159909 -2.885290 2.078125
C 5.069396 -1.428633 0.250914
C 1.544078 -1.910335 0.879264
O 1.281546 -2.997975 1.364956
H -0.320339 -1.742496 2.268264
H -0.471131 0.007675 2.023903
H 0.324532 1.138302 -1.909324
H -0.410601 -0.440687 -1.692809
H -2.560561 -0.104771 -2.136096
H -4.724252 1.059406 -1.911863
H -4.917130 3.052526 -0.454023
H -5.777489 -1.480357 0.198856
H -5.008043 0.222163 1.824901
H -2.603202 0.454891 2.324382
H -5.284436 -3.290983 -1.345641
H -3.902645 4.661807 1.054650

H  -1.926768  5.564500  2.230183
H  0.288086  4.451855  1.960907
H  0.514839  2.453229  0.576205
H  -0.480880  -3.137382  -0.112045
H  -1.277737  -4.783038  -1.728804
H  -3.682278  -4.861316  -2.379849
H  4.028158  3.332402  1.057788
H  4.246649  4.437326  -0.316707
H  2.615884  4.114687  0.312367
H  3.371875  3.721492  -2.633953
H  1.785096  3.365838  -1.925002
H  2.659186  2.113291  -2.847701
H  4.722474  1.023117  -1.827557
H  5.488197  2.602478  -1.555810
H  5.255500  1.490780  -0.194693
H  4.640675  -4.023102  -0.467883
H  2.915697  -4.126409  -0.063429
H  3.469347  -3.005458  -1.333221
H  3.348810  -3.544373  2.387700
H  4.240666  -2.058753  2.791534
H  5.101081  -3.444687  2.094527
H  6.007808  -1.991043  0.241010
H  4.887172  -1.025389  -0.747552
H  5.169936  -0.597352  0.955390

**C3_GP2_CC**
Inner Energy (Hartree)      : -1651.055976
Zero Point Energy (Hartree) : 0.629470
Enthalpy (Hartree)          : -1650.390522
Entropy (Hartree)           : 0.095861
Gibbs Energy (Hartree)      : -1650.486383
Minimum geometry
N  0.764177  1.817732  -0.299079
N  0.533751  0.453835  -0.139220
C  0.116279  0.110927  1.233673
C  -0.004532  2.527236  -1.328980
C  -1.467481  2.175398  -1.310689
C  -0.288085  -1.315783  1.526428
C  -2.015274  1.544602  -2.401570
C  -3.388130  1.210068  -2.448372
C  -4.198268  1.491542  -1.381585
C  -3.676429  2.138676  -0.230227
C  -2.300083  2.502291  -0.194092
C  -1.378921  -1.958661  0.861663
C  -1.731072  -3.286943  1.238064
C  -1.013443  -3.935757  2.278473
C  0.016523  -3.294880  2.910388
C  0.377559  -1.983776  2.524883
C  -2.790901  -3.943275  0.559691
C  -4.503440  2.437784  0.884010
C  -3.996436  3.077207  1.984996
C  -2.634199  3.453570  2.017709
C  -1.808364  3.175474  0.957975
C  -2.118769  -1.332393  -0.175166
C  -3.138132  -1.993947  -0.813161
C  -3.477884  -3.315461  -0.446880
O  2.748977  1.387538  0.639850
C  4.203106  1.455291  0.744842
C  4.531539  0.153114  1.469742
C  4.615770  2.667356  1.574562
C  4.813588  1.464811  -0.654406
C  2.052691  2.282707  -0.065054
O  2.433961  3.375155  -0.432181
O  1.372457  -1.585174  -0.544283
C  1.744275  -2.695513  -1.417500
C  1.621211  -3.901230  -0.491684
C  3.182287  -2.529268  -1.903359
C  0.741911  -2.788103  -2.565023
C  1.268176  -0.345400  -1.003023
O  1.724013  0.083712  -2.047452
H  0.926792  0.390001  1.914958
H  -0.729513  0.774526  1.440025
H  0.145031  3.592814  -1.141630
H  0.418481  2.300932  -2.312847
H  -1.376790  1.288888  -3.242693
H  -3.789147  0.718072  -3.329330
H  -5.251623  1.224587  -1.400000
H  -1.295837  -4.946947  2.559712
H  0.567178  -3.788778  3.705257
H  1.211260  -1.498873  3.025859
H  -3.047175  -4.958105  0.853422
H  -5.550781  2.149605  0.847347
H  -4.637720  3.299512  2.832370
H  -2.239744  3.966947  2.889406
H  -0.762638  3.460160  1.003515
H  -1.864531  -0.322981  -0.476050
H  -3.687438  -1.492792  -1.603688
H  -4.284838  -3.828558  -0.961547
H  4.161469  -0.702725  0.896416
H  5.614017  0.054548  1.594558
H  4.063367  0.135636  2.459168
H  5.696853  2.639958  1.746184
H  4.112829  2.649762  2.546912
H  4.361549  3.595349  1.060322
H  4.591651  2.398344  -1.174922
H  5.900036  1.355700  -0.578089
H  4.415697  0.631474  -1.242104
H  1.895703  -4.812675  -1.031810
H  2.284897  -3.790750  0.371180
H  0.595787  -4.001972  -0.125939
H  3.855451  -2.391156  -1.050476
H  3.277162  -1.673036  -2.571840
H  3.490334  -3.434455  -2.437113
H  0.938935  -3.692085  -3.150014
H  -0.277303  -2.845737  -2.167991
H  0.819396  -1.921980  -3.225867

**C3_CIS_CC**
Inner Energy (Hartree)      : -1650.996204
Zero Point Energy (Hartree) : 0.630731
Enthalpy (Hartree)          : -1650.331221
Entropy (Hartree)           : 0.091404
Gibbs Energy (Hartree)      : -1650.422625
Imaginary frequency (cm-1): -18.34
N  0.258267  0.487489  -0.796335
N  0.142646  0.160450  0.596109
C  1.093650  -0.863545  1.101356
C  0.554014  -0.638689  -1.706354
C  -0.221401  -1.923831  -1.457922
C  2.541129  -0.960715  0.616143
C  0.498026  -3.091650  -1.353305
C  -0.110561  -4.315925  -0.998243
C  -1.447976  -4.353253  -0.712279
C  -2.245290  -3.188434  -0.861406
C  -1.644170  -1.971368  -1.300000
C  3.426925  0.112022  0.265565
C  4.772634  -0.208559  -0.099754
C  5.217334  -1.554812  -0.082704
C  4.364095  -2.558087  0.283294
C  3.030526  -2.250234  0.621650
C  5.667399  0.829495  -0.471818
C  -3.640128  -3.222279  -0.597404
C  -4.423614  -2.116952  -0.800928
C  -3.847084  -0.937869  -1.324068
C  -2.500026  -0.869623  -1.571647
C  3.052799  1.483625  0.251176
C  3.943973  2.462440  -0.108180
C  5.266881  2.138560  -0.479359
O  -1.416620  2.031055  -0.595411
C  -2.038900  3.314198  -0.944965
C  -2.841912  3.662937  0.302796
C  -2.964088  3.121332  -2.143917
C  -0.961949  4.367992  -1.192310
C  -0.490638  1.501967  -1.383190
O  -0.312445  1.799821  -2.552969
O  -0.487089  0.757925  2.720596
C  -1.736913  0.003286  2.919822
C  -1.942319  0.120567  4.426754
C  -1.620904  -1.462759  2.511150
C  -2.860942  0.688609  2.154854
C  -0.054771  1.201000  1.523282
O  0.206440  2.369542  1.359604
H  1.111524  -0.706522  2.180268
H  0.645109  -1.844541  0.945215
H  0.354627  -0.238314  -2.700609
H  1.621614  -0.861187  -1.669514

H 1.573872 -3.063917 -1.498946
H 0.497520 -5.211595 -0.916065
H -1.922153 -5.275362 -0.386865
H 6.245313 -1.771029 -0.361187
H 4.697614 -3.590991 0.305763
H 2.365311 -3.066175 0.892906
H 6.683228 0.560471 -0.749775
H -4.078005 -4.150679 -0.239887
H -5.488958 -2.153540 -0.595033
H -4.478903 -0.083287 -1.547240
H -2.100097 0.020421 -2.037872
H 2.052082 1.783375 0.522633
H 3.622459 3.499608 -0.106688
H 5.959605 2.924177 -0.765666
H -2.184316 3.705987 1.175200
H -3.320025 4.638078 0.168934
H -3.623017 2.918509 0.482222
H -3.530257 4.042544 -2.316814
H -3.678437 2.315957 -1.941196
H -2.398188 2.877721 -3.043548
H -0.394747 4.155425 -2.099494
H -1.439523 5.347059 -1.300499
H -0.277664 4.404030 -0.339960
H -2.867839 -0.385656 4.717761
H -1.108853 -0.342293 4.964120
H -2.007565 1.171171 4.722928
H -0.777992 -1.949041 3.011148
H -1.518081 -1.570759 1.429780
H -2.536819 -1.981132 2.813098
H -3.806480 0.176395 2.359936
H -2.953918 1.732245 2.466805
H -2.681495 0.653854 1.076630

**C3_GP1**
Inner Energy (Hartree) : -1651.069164
Zero Point Energy (Hartree) : 0.630236
Enthalpy (Hartree) : -1650.403362
Entropy (Hartree) : 0.094309
Gibbs Energy (Hartree) : -1650.497671
Minimum geometry
N -0.196269 -0.658601 1.027873
N 0.196170 0.657942 1.028202
C 1.200813 1.046842 2.013436
C -1.200963 -1.048002 2.012874
C -2.520580 -0.338190 1.815367
C 2.520491 0.337249 1.815513
C -2.788854 0.781647 2.564013
C -3.991937 1.507753 2.406075
C -4.925190 1.090859 1.496552
C -4.692849 -0.062907 0.699967
C -3.474644 -0.786644 0.847684
C 3.474508 0.786315 0.848061
C 4.692792 0.062786 0.699961
C 4.925261 -1.091368 1.495946
C 3.992055 -1.508839 2.405251
C 2.788889 -0.782953 2.563568
C 5.652955 0.503377 -0.248110
C -5.653055 -0.502893 -0.248341
C -5.420293 -1.610095 -1.022959
C -4.208397 -2.323318 -0.884989
C -3.259636 -1.923329 0.022427
C 3.259398 1.923428 0.023418
C 4.208126 2.324007 -0.883773
C 5.420090 1.610975 -1.022130
O 1.146480 -0.962367 -0.728780
C 1.779527 -1.709212 -1.812994
C 2.638390 -0.651148 -2.496876
C 0.709235 -2.232052 -2.768703
C 2.656112 -2.820355 -1.240874
C 0.305394 -1.555820 0.110468
O -0.023750 -2.730871 0.107139
O -1.146537 0.962621 -0.728325
C -1.779314 1.709935 -1.812371
C -0.708797 2.232892 -2.767764
C -2.655791 2.821040 -1.240013
C -2.638252 0.652240 -2.496731
C -0.305467 1.555635 0.111245
O 0.023628 2.730703 0.108578

H 1.293640 2.130470 1.950707
H 0.814122 0.811478 3.011986
H -1.293883 -2.131584 1.949514
H -0.814255 -0.813249 3.011560
H -2.059487 1.120439 3.295862
H -4.168901 2.391006 3.011858
H -5.856846 1.636023 1.368954
H 5.856979 -1.636359 1.368071
H 4.169120 -2.392386 3.010576
H 2.059556 -1.122213 3.295235
H 6.579039 -0.056639 -0.350167
H -6.579080 0.057272 -0.350104
H -6.161389 -1.938037 -1.745649
H -4.022867 -3.192556 -1.508798
H -2.326363 -2.472821 0.090947
H 2.326085 2.472811 0.092232
H 4.022513 3.193566 -1.507108
H 6.161155 1.939378 -1.744642
H 3.413556 -0.283735 -1.820596
H 3.119296 -1.082784 -3.380251
H 2.024665 0.198014 -2.812603
H 1.191440 -2.696479 -3.634869
H 0.088520 -1.403201 -3.125570
H 0.070880 -2.971016 -2.282401
H 2.053324 -3.599979 -0.773072
H 3.247102 -3.265453 -2.048148
H 3.344335 -2.405394 -0.497236
H -1.190797 2.697676 -3.633853
H -0.070368 2.971602 -2.281175
H -0.088184 1.404039 -3.124799
H -2.052936 3.600443 -0.771931
H -3.344133 2.405958 -0.496552
H -3.246655 3.266442 -2.047212
H -3.118950 1.084247 -3.380036
H -2.024615 -0.196917 -2.812645
H -3.413582 0.284730 -1.820692

**C3_TRANS_CC**
Inner Energy (Hartree) : -1651.004840
Zero Point Energy (Hartree) : 0.629336
Enthalpy (Hartree) : -1650.340567
Entropy (Hartree) : 0.093636
Gibbs Energy (Hartree) : -1650.434203
Imaginary frequency (cm-1): -20.25
N -0.982004 1.201237 -0.977189
N 0.287393 0.576040 -1.338253
C 1.458448 1.022963 -0.541630
C -2.108181 0.645339 -1.752121
C -2.859092 -0.552605 -1.192570
C 2.653712 0.126411 -0.787393
C -3.083228 -1.602901 -2.052275
C -3.876165 -2.713110 -1.689816
C -4.427742 -2.777850 -0.440219
C -4.216555 -1.728212 0.491759
C -3.443959 -0.587910 0.115687
C 3.525125 -0.255765 0.283669
C 4.655459 -1.075296 -0.012423
C 4.894174 -1.489577 -1.348393
C 4.052662 -1.101304 -2.354697
C 2.936936 -0.285070 -2.069309
C 5.519241 -1.483674 1.038063
C -4.767185 -1.800713 1.798528
C -4.580701 -0.786135 2.699905
C -3.847503 0.360135 2.320037
C -3.298591 0.458911 1.066520
C 3.320332 0.123608 1.639946
C 4.171384 -0.290098 2.633485
C 5.286125 -1.104568 2.333366
O 0.014765 3.188414 -0.444335
C 0.038667 4.501877 0.201436
C 1.535351 4.752584 0.369237
C -0.648647 4.429946 1.562819
C -0.585919 5.548313 -0.717008
C -1.126404 2.540667 -0.656352
O -2.234052 3.046990 -0.551285
O 0.184707 -1.200766 0.123283
C 0.298897 -2.597893 0.574685
C 0.437019 -2.435959 2.084137

C 1.546855 -3.239755 -0.024761
C -0.967162 -3.364702 0.219761
C 0.136363 -0.869787 -1.154515
O 0.026992 -1.608616 -2.099513
H 1.715254 2.025668 -0.868305
H 1.196307 1.067803 0.517168
H -2.814807 1.466102 -1.879440
H -1.712519 0.381445 -2.737836
H -2.625535 -1.586513 -3.037033
H -4.029400 -3.516355 -2.403939
H -5.028093 -3.632777 -0.140063
H 5.755054 -2.119687 -1.555485
H 4.234099 -1.417082 -3.377318
H 2.271475 0.006974 -2.875376
H 6.372934 -2.109910 0.791993
H -5.343939 -2.681372 2.069890
H -5.004575 -0.853328 3.697445
H -3.723521 1.176766 3.025338
H -2.777174 1.363671 0.787073
H 2.478263 0.751148 1.907513
H 3.986928 0.012258 3.659994
H 5.951976 -1.425760 3.128432
H 2.037841 4.749483 -0.603057
H 1.698980 5.726190 0.840675
H 1.988466 3.980432 0.999245
H -0.484387 5.370168 2.098668
H -0.221438 3.616868 2.159446
H -1.722358 4.269249 1.457633
H -1.654578 5.371851 -0.843095
H -0.437970 6.543363 -0.284729
H -0.101938 5.525446 -1.698605
H 0.520752 -3.419979 2.555473
H 1.332163 -1.854659 2.327115
H -0.440199 -1.925450 2.493904
H 2.442801 -2.674930 0.244133
H 1.478744 -3.303898 -1.112178
H 1.645696 -4.253236 0.377400
H -0.905574 -4.370405 0.648614
H -1.845564 -2.865912 0.635413
H -1.089486 -3.449978 -0.860572

**C3_TRANS_C**
Inner Energy (Hartree) : -1651.008937
Zero Point Energy (Hartree) : 0.629596
Enthalpy (Hartree) : -1650.344638
Entropy (Hartree) : 0.092438
Gibbs Energy (Hartree) : -1650.437076
Imaginary frequency (cm-1): -7.17
N 0.090383 0.016751 1.208424
N 0.232120 1.410873 1.524896
C 1.585682 1.977457 1.603421
C -1.044203 -0.632488 1.920943
C -2.343758 -0.904984 1.188720
C 2.639536 1.545247 0.580026
C -3.494574 -0.745777 1.925071
C -4.757019 -1.155180 1.440212
C -4.858455 -1.730353 0.203249
C -3.704468 -1.891335 -0.607992
C -2.428425 -1.474549 -0.123604
C 2.423250 1.234033 -0.805279
C 3.558780 0.929489 -1.620896
C 4.864554 0.969958 -1.070649
C 5.046294 1.269638 0.251597
C 3.929008 1.536560 1.066895
C 3.374856 0.577499 -2.985656
C -3.812175 -2.436676 -1.914237
C -2.712335 -2.555025 -2.723117
C -1.449801 -2.130704 -2.252576
C -1.309723 -1.609141 -0.990690
C 1.143873 1.178702 -1.428838
C 0.999247 0.829741 -2.746694
C 2.125491 0.523297 -3.541960
O 1.408429 -1.684157 0.659143
C 2.400144 -2.754779 0.785549
C 2.169783 -3.549454 -0.496158
C 2.089948 -3.593980 2.021137
C 3.803209 -2.158370 0.811105
C 1.256191 -0.744224 1.575546
O 1.913493 -0.590746 2.577730
O -1.689584 1.703096 0.346283
C -2.722702 2.518632 -0.310361
C -2.070779 3.509224 -1.274887
C -3.587487 3.198650 0.746932
C -3.540567 1.498861 -1.092842
C -0.728123 2.297481 1.049443
O -0.653362 3.491195 1.281731
H 1.427596 3.053271 1.520973
H 1.991081 1.780530 2.595446
H -0.681754 -1.608096 2.266923
H -1.249335 -0.047083 2.824099
H -3.434427 -0.306092 2.917727
H -5.638651 -1.013988 2.058087
H -5.821137 -2.056040 -0.182013
H 5.712028 0.747870 -1.713798
H 6.042024 1.289841 0.683595
H 4.093436 1.742009 2.121279
H 4.254628 0.346513 -3.580954
H -4.792070 -2.749483 -2.266173
H -2.809469 -2.966417 -3.723557
H -0.577970 -2.208971 -2.896226
H -0.338807 -1.270345 -0.661377
H 0.247888 1.368803 -0.861482
H 0.002306 0.776720 -3.174838
H 1.997819 0.246881 -4.584230
H 2.358940 -2.919022 -1.370723
H 2.845904 -4.408943 -0.529740
H 1.137477 -3.909494 -0.544870
H 2.759272 -4.459514 2.047749
H 1.058787 -3.960752 1.982966
H 2.232532 -3.017132 2.937118
H 3.977939 -1.586383 1.723304
H 4.537371 -2.968757 0.754900
H 3.948365 -1.498064 -0.048993
H -2.854726 4.006059 -1.855372
H -1.490623 4.265734 -0.747342
H -1.414547 2.979545 -1.974185
H -3.021812 3.947829 1.302108
H -3.976907 2.449524 1.444166
H -4.437287 3.686594 0.258477
H -4.287040 2.024640 -1.696501
H -2.898913 0.914514 -1.759264
H -4.060754 0.814530 -0.420089

**D4_GP2_C**
Inner Energy (Hartree) : -1036.647928
Zero Point Energy (Hartree) : 0.435667
Enthalpy (Hartree) : -1036.185469
Entropy (Hartree) : 0.077988
Gibbs Energy (Hartree) : -1036.263457
Minimum geometry
N 1.051665 0.406244 0.558162
N 1.051685 -0.405884 -0.558358
C 1.793096 0.033398 -1.731587
C 1.793069 -0.032982 1.731420
C 3.145845 0.604796 1.895698
C 3.146071 -0.604019 -1.895629
C 3.648789 -1.566938 -1.132444
C 3.648506 1.567740 1.132508
O -0.640480 1.483975 -0.422452
C -1.750027 2.426718 -0.522405
C -2.285661 2.153357 -1.924808
C -1.227107 3.856374 -0.407222
C -2.810382 2.110889 0.530367
C 0.073827 1.371584 0.700002
O -0.061584 2.016858 1.720846
O -0.640197 -1.483931 0.422350
C -1.749285 -2.427195 0.522494
C -2.284843 -2.154056 1.924967
C -1.225740 -3.856621 0.407272
C -2.809921 -2.111865 -0.530144
C 0.073936 -1.371310 -0.700191
O -0.061290 -2.016751 -1.720956
H 1.180691 -0.173815 -2.614901
H 1.893636 1.121087 -1.661884
H 1.180512 0.173923 2.614703
H 1.893935 -1.120630 1.661567

H 3.723472 0.219518 2.735066
H 3.723795 -0.218505 -2.734822
H 4.633690 -1.981831 -1.321646
H 3.092322 -1.974584 -0.293156
H 4.633266 1.982889 1.321882
H 3.092128 1.975154 0.293049
H -3.137552 2.807352 -2.133828
H -1.510751 2.338873 -2.674806
H -2.612529 1.112695 -2.014640
H -2.043167 4.559528 -0.602476
H -0.439232 4.031190 -1.147102
H -0.828290 4.048365 0.590144
H -3.105132 1.058442 0.465908
H -2.442194 2.313506 1.536713
H -3.697047 2.725944 0.346278
H -1.509744 -2.339222 2.674857
H -2.612165 -1.113543 2.014845
H -3.136411 -2.808431 2.134112
H -2.041456 -4.560135 0.602666
H -0.437679 -4.031063 1.147044
H -0.826981 -4.048461 -0.590147
H -3.696283 -2.727318 -0.345929
H -3.105142 -1.059549 -0.465672
H -2.441775 -2.314336 -1.536536

**D4_CIS_C**
Inner Energy (Hartree) : -1036.599730
Zero Point Energy (Hartree) : 0.436374
Enthalpy (Hartree) : -1036.138095
Entropy (Hartree) : 0.074447
Gibbs Energy (Hartree) : -1036.212543
Imaginary frequency (cm-1): -37.83
N -0.169355 1.244542 -0.207359
N 1.265714 1.092642 -0.288879
C 1.881321 2.200158 -1.027226
C -0.477471 2.417198 0.649478
C -1.778486 3.075688 0.281360
C 2.736290 3.117860 -0.195564
C 3.007439 2.964757 1.096028
C -2.470737 2.880199 -0.837505
O -1.835233 -0.237534 -0.501587
C -3.066119 -0.927654 -0.089833
C -3.883685 -0.920567 -1.377503
C -3.771430 -0.123644 1.000615
C -2.769165 -2.351917 0.360006
C -0.918477 0.133142 0.376432
O -0.807369 -0.249055 1.514968
O 1.172174 -1.172805 -0.204448
C 1.792913 -2.483007 0.022879
C 0.599586 -3.379945 0.321576
C 2.719268 -2.410872 1.234568
C 2.510114 -2.951787 -1.241084
C 1.941478 -0.101112 -0.394209
O 3.134236 -0.118114 -0.647604
H 2.497225 1.781373 -1.829208
H 1.070435 2.757685 -1.509087
H -0.465051 2.135710 1.711073
H 0.321164 3.152097 0.529984
H -2.127878 3.801784 1.014057
H 3.160634 3.954984 -0.748408
H 3.645363 3.664788 1.626339
H 2.604051 2.127230 1.658914
H -3.385022 3.433546 -1.030982
H -2.150391 2.156775 -1.580148
H -4.840665 -1.425040 -1.213427
H -4.079979 0.106333 -1.700250
H -3.347034 -1.441712 -2.175977
H -4.757768 -0.562317 1.181712
H -3.903627 0.914350 0.682342
H -3.209619 -0.139054 1.936391
H -2.256849 -2.906641 -0.430367
H -2.153626 -2.356493 1.260482
H -3.713571 -2.861140 0.578190
H 0.051752 -3.008728 1.191093
H -0.078076 -3.411206 -0.535867
H 0.944798 -4.397536 0.527805
H 3.065473 -3.419202 1.483144
H 2.176184 -2.010421 2.096696
H 3.588293 -1.782575 1.034998
H 2.837315 -3.987943 -1.104726
H 1.826145 -2.918731 -2.095423
H 3.378326 -2.329123 -1.456412

**D4_GP2_CC**
Inner Energy (Hartree) : -1036.647470
Zero Point Energy (Hartree) : 0.435849
Enthalpy (Hartree) : -1036.184630
Entropy (Hartree) : 0.078898
Gibbs Energy (Hartree) : -1036.263528
Minimum geometry
N 0.488917 1.524618 -0.347744
N -0.459426 0.711060 0.242869
C -0.752097 0.923502 1.661625
C 0.015519 2.409808 -1.414475
C -1.116849 3.268262 -0.939661
C -2.053141 1.639531 1.893293
C -2.182316 2.713183 2.666275
C -2.297833 3.330543 -1.546345
O 1.968977 0.051486 0.450074
C 3.160350 -0.790319 0.404014
C 2.842708 -1.861766 1.443315
C 4.390191 0.016792 0.811082
C 3.298873 -1.413413 -0.983294
C 1.790615 1.044518 -0.424047
O 2.628166 1.528000 -1.157885
O -1.672852 -1.149119 0.272135
C -2.167804 -2.436101 -0.209720
C -3.095827 -2.877630 0.918193
C -1.006684 -3.415750 -0.367219
C -2.948552 -2.244193 -1.507809
C -0.796379 -0.433612 -0.441337
O -0.356263 -0.708915 -1.542213
H -0.765957 -0.053813 2.152848
H 0.084493 1.492186 2.073323
H 0.873919 3.021853 -1.700404
H -0.282514 1.813822 -2.285777
H -0.926746 3.860568 -0.046360
H -2.919490 1.210462 1.393317
H -3.146391 3.185796 2.829483
H -1.326451 3.159366 3.168817
H -3.088277 3.982833 -1.187162
H -2.515256 2.732784 -2.428896
H 3.676438 -2.565985 1.521715
H 2.676093 -1.407716 2.425055
H 1.942967 -2.415500 1.156935
H 5.255918 -0.649856 0.882312
H 4.232156 0.480434 1.790289
H 4.604200 0.796677 0.078955
H 2.352848 -1.878280 -1.278400
H 3.569258 -0.664771 -1.729586
H 4.076699 -2.183302 -0.959259
H -2.542049 -2.973569 1.857185
H -3.900680 -2.150063 1.060693
H -3.541460 -3.847085 0.675913
H -1.399983 -4.410561 -0.599900
H -0.439976 -3.482159 0.567564
H -0.336090 -3.106656 -1.170351
H -3.423840 -3.189776 -1.788064
H -3.733508 -1.493808 -1.368311
H -2.291628 -1.925371 -2.318025

**D4_CIS_CC**
Inner Energy (Hartree) : -1036.601422
Zero Point Energy (Hartree) : 0.435936
Enthalpy (Hartree) : -1036.139910
Entropy (Hartree) : 0.075422
Gibbs Energy (Hartree) : -1036.215333
Imaginary frequency (cm-1): -59.66
N 1.582035 0.241255 -0.187315
N 0.778080 -0.937382 -0.047638
C 1.431962 -1.891107 0.878942
C 2.887082 -0.060998 -0.774044
C 2.783828 -0.905841 -2.013420
C 1.893080 -1.310171 2.188238
C 3.123848 -1.479703 2.662121
C 3.544114 -1.972133 -2.242086

O -0.204939 1.617851 -0.191720
C -0.826533 2.921165 0.065313
C -2.288841 2.564712 0.297820
C -0.685219 3.823169 -1.158625
C -0.223624 3.538259 1.325182
C 1.120967 1.526618 -0.318816
O 1.874258 2.462452 -0.536836
O -1.354735 -1.327969 -0.593490
C -2.712912 -1.825028 -0.340170
C -3.096567 -2.424727 -1.689455
C -3.662018 -0.694222 0.035919
C -2.653557 -2.906499 0.736680
C -0.606197 -0.814328 0.371860
O -0.980045 -0.481544 1.471043
H 0.696947 -2.687103 1.046028
H 2.267772 -2.357165 0.346880
H 3.332319 0.908480 -1.001729
H 3.525914 -0.543630 -0.026678
H 2.056369 -0.576531 -2.753460
H 1.153356 -0.741782 2.745832
H 3.428502 -1.071433 3.621177
H 3.875061 -2.041786 2.109335
H 3.477012 -2.534615 -3.168324
H 4.271546 -2.317931 -1.509709
H -2.856899 3.470606 0.530106
H -2.717601 2.108749 -0.598754
H -2.382843 1.862691 1.129922
H -1.283226 4.728342 -1.008693
H -1.060541 3.308863 -2.049353
H 0.354380 4.105523 -1.323632
H -0.298474 2.832942 2.159147
H 0.823288 3.805944 1.174857
H -0.782311 4.442558 1.586695
H -3.085294 -1.655572 -2.467470
H -2.396565 -3.216359 -1.972273
H -4.103082 -2.850265 -1.634418
H -4.678326 -1.094335 0.112127
H -3.657742 0.079559 -0.736298
H -3.388308 -0.248284 0.992431
H -3.638183 -3.374296 0.833406
H -1.933149 -3.682256 0.456715
H -2.373522 -2.489342 1.706075

**D4_GP1**
Inner Energy (Hartree) : -1036.652089
Zero Point Energy (Hartree) : 0.436487
Enthalpy (Hartree) : -1036.188985
Entropy (Hartree) : 0.077455
Gibbs Energy (Hartree) : -1036.266440
Minimum geometry
N -0.196659 -1.384108 0.660979
N 0.196675 -1.384151 -0.660913
C -0.551599 -2.249437 -1.569702
C 0.551636 -2.249318 1.569820
C 1.906004 -1.703834 1.927468
C -1.905965 -1.703995 -1.927422
C -3.050277 -2.320044 -1.647991
C 3.050315 -2.319900 1.648071
O -1.440350 0.412146 0.193413
C -2.368204 1.502578 0.462852
C -2.619236 2.072625 -0.930252
C -1.705322 2.543098 1.362462
C -3.661407 0.951712 1.059408
C -0.989697 -0.374726 1.168864
O -1.221853 -0.269718 2.359046
O 1.440346 0.412129 -0.193436
C 2.368172 1.502574 -0.462914
C 2.619213 2.072651 0.930176
C 1.705259 2.543065 -1.362534
C 3.661377 0.951719 -1.059477
C 0.989718 -0.374802 -1.168852
O 1.221872 -0.269850 -2.359039
H 0.063772 -2.366107 -2.464296
H -0.645527 -3.228075 -1.088147
H -0.063718 -2.365920 2.464436
H 0.645561 -3.227993 1.088341
H 1.903199 -0.746495 2.444331
H -1.903157 -0.746705 -2.444375
H -4.009994 -1.899773 -1.935175
H -3.069928 -3.273029 -1.123220
H 4.010034 -1.899596 1.935197
H 3.069963 -3.272933 1.123387
H -3.286583 2.937343 -0.866180
H -1.678473 2.389528 -1.391111
H -3.084677 1.319669 -1.573563
H -2.365124 3.410788 1.463972
H -0.762580 2.879525 0.918388
H -1.504114 2.133926 2.353489
H -4.053889 0.152218 0.422491
H -3.496240 0.557512 2.063195
H -4.407744 1.750982 1.111444
H 1.678449 2.389543 1.391042
H 3.084676 1.319715 1.573495
H 3.286541 2.937381 0.866080
H 2.365050 3.410760 -1.464080
H 0.762523 2.879494 -0.918447
H 1.504034 2.133868 -2.353547
H 4.407703 1.750997 -1.111525
H 4.053874 0.152235 -0.422556
H 3.496208 0.557508 -2.063259

**D4_TRANS_CC**
Inner Energy (Hartree) : -1036.608293
Zero Point Energy (Hartree) : 0.435993
Enthalpy (Hartree) : -1036.146715
Entropy (Hartree) : 0.075745
Gibbs Energy (Hartree) : -1036.222460
Imaginary frequency (cm-1): -25.76
N 0.627912 1.106756 -0.715585
N -0.284948 0.048059 -0.351364
C 0.039754 -1.327372 -0.797766
C 0.176515 2.453083 -0.279013
C -0.192404 2.478346 1.180239
C 0.268583 -2.302419 0.322277
C 0.084896 -2.056019 1.613452
C -1.375663 2.874325 1.640434
O 2.357053 -0.282272 -0.124549
C 3.767295 -0.640365 0.050105
C 4.533778 -0.436412 -1.254863
C 3.698762 -2.123858 0.399763
C 4.359328 0.156524 1.209919
C 2.002567 0.943806 -0.491913
O 2.773947 1.871481 -0.657079
O -2.349630 -0.611174 0.060003
C -3.800733 -0.686042 -0.069563
C -4.133481 -1.939637 0.734982
C -4.194958 -0.864503 -1.534136
C -4.443787 0.547243 0.561463
C -1.641649 0.342403 -0.550365
O -2.107731 1.280323 -1.162578
H -0.788594 -1.697689 -1.412242
H 0.905717 -1.287717 -1.456924
H 1.031313 3.100742 -0.466381
H -0.650654 2.777504 -0.901580
H 0.585505 2.158577 1.874074
H 0.588174 -3.290666 -0.007261
H 0.256396 -2.821563 2.364183
H -0.241621 -1.077152 1.952244
H -1.591361 2.909600 2.704427
H -2.170181 3.179643 0.963812
H 5.541565 -0.849960 -1.145697
H 4.034955 -0.966873 -2.072923
H 4.612179 0.620238 -1.511131
H 4.709082 -2.506646 0.573666
H 3.247542 -2.693244 -0.419149
H 3.097831 -2.281357 1.298859
H 4.413719 1.219112 0.970574
H 3.748233 0.021605 2.108287
H 5.368409 -0.210734 1.423205
H -3.624793 -2.811724 0.313050
H -3.813853 -1.822928 1.774809
H -5.212584 -2.119826 0.717851
H -5.272968 -1.043398 -1.599603
H -3.679280 -1.730597 -1.962579
H -3.952170 0.022229 -2.121699
H -5.528182 0.405138 0.613263

H -4.065104 0.688322 1.578933
H -4.231953 1.444167 -0.021876

**D4_TRANS_C**
Inner Energy (Hartree) : -1036.602635
Zero Point Energy (Hartree) : 0.436273
Enthalpy (Hartree) : -1036.140756
Entropy (Hartree) : 0.075644
Gibbs Energy (Hartree) : -1036.216400
Imaginary frequency (cm-1): -40.72
N -0.416928 -0.585655 -0.214906
N 0.066457 0.647947 -0.805014
C -0.352946 1.883768 -0.067741
C 0.306658 -1.827957 -0.555599
C 0.880243 -2.502465 0.659004
C -0.000385 1.867649 1.393501
C 0.786515 2.772721 1.969355
C 0.905659 -3.820187 0.830236
O -2.525383 0.271603 -0.365135
C -3.968175 0.272567 -0.118806
C -4.362701 1.703964 -0.473482
C -4.249090 -0.012527 1.354480
C -4.657737 -0.721016 -1.050419
C -1.782792 -0.803239 -0.101693
O -2.215078 -1.893645 0.226591
O 2.182368 0.040029 -0.072366
C 3.651636 0.074646 -0.062881
C 4.008561 -0.900135 1.054420
C 4.126143 1.483941 0.282992
C 4.192452 -0.420074 -1.401518
C 1.485229 0.767256 -0.937093
O 1.942458 1.549919 -1.738742
H 0.155084 2.700851 -0.582116
H -1.420513 2.014910 -0.211533
H -0.386335 -2.504406 -1.060504
H 1.092549 -1.584342 -1.271199
H 1.296745 -1.836753 1.409520
H -0.431329 1.063977 1.987476
H 1.004388 2.745155 3.033266
H 1.236898 3.581857 1.397831
H 1.353384 -4.268632 1.712118
H 0.475404 -4.500913 0.098783
H -5.444123 1.828928 -0.363950
H -3.864415 2.419584 0.188432
H -4.088451 1.933133 -1.507672
H -5.315548 0.134347 1.553186
H -3.684584 0.680767 1.987140
H -3.979124 -1.035981 1.617606
H -4.387336 -0.512566 -2.090547
H -4.376578 -1.746190 -0.807751
H -5.743038 -0.616692 -0.950425
H 3.560532 -0.580387 2.000442
H 3.652581 -1.907694 0.821105
H 5.095142 -0.933095 1.178381
H 5.209633 1.470201 0.438643
H 3.647126 1.822840 1.207123
H 3.893321 2.188303 -0.516340
H 5.281687 -0.507872 -1.337576
H 3.785097 -1.410117 -1.632473
H 3.944560 0.268025 -2.210830

**E5_GP2_C**
Inner Energy (Hartree) : -1491.864232
Zero Point Energy (Hartree) : 0.495369
Enthalpy (Hartree) : -1491.336652
Entropy (Hartree) : 0.089011
Gibbs Energy (Hartree) : -1491.425663
Minimum geometry
N -0.489087 -1.690363 0.084655
N -0.109554 -0.363565 0.122230
C -0.537775 0.392035 1.235727
C 0.524102 -2.682329 0.013847
C 1.881266 -2.265258 0.491588
C -0.684386 1.861097 1.046395
O 0.309016 -3.776309 -0.445390
O -0.844748 -0.167609 2.266917
C 2.972789 -2.624362 -0.300935
C 4.261454 -2.292678 0.101214
C 4.462914 -1.620062 1.304816
C 3.374652 -1.282092 2.106956
C 2.082633 -1.598495 1.701758
C -1.206707 2.393779 -0.134219
C -1.379635 3.766953 -0.255345
C -1.018703 4.610371 0.794681
C -0.508374 4.076834 1.976195
C -0.355282 2.700961 2.109046
O -2.512845 -0.864754 -0.413567
C -3.981872 -0.834888 -0.416168
C -4.516848 -1.729748 -1.530097
C -4.284454 0.631541 -0.705282
C -4.501606 -1.229205 0.963748
C -1.860704 -1.983831 -0.111705
O -2.318161 -3.086715 0.030627
O 1.418290 1.107324 -0.551108
C 2.313556 1.837107 -1.456138
C 1.544512 2.335328 -2.675821
C 3.481131 0.927443 -1.822953
C 2.762720 3.003643 -0.583040
C 0.709246 0.071108 -0.948390
O 0.750926 -0.517709 -2.000876
H 2.799556 -3.152195 -1.232937
H 5.108023 -2.560624 -0.523106
H 5.469793 -1.364686 1.621452
H 3.531723 -0.771859 3.052140
H 1.232878 -1.340797 2.326330
H -1.488652 1.732507 -0.948028
H -1.795462 4.179608 -1.169261
H -1.144398 5.684129 0.694596
H -0.234718 4.732463 2.796723
H 0.029331 2.270756 3.028037
H -4.070225 -1.448674 -2.489077
H -4.301169 -2.780246 -1.331824
H -5.601313 -1.599910 -1.604571
H -5.367309 0.782665 -0.743504
H -3.859416 0.930744 -1.668649
H -3.869977 1.274539 0.077037
H -5.578920 -1.041390 1.008456
H -4.323205 -2.285196 1.171751
H -4.012207 -0.626559 1.735587
H 0.683139 2.933410 -2.362139
H 2.203461 2.971860 -3.274513
H 1.201039 1.507748 -3.298623
H 3.146466 0.085406 -2.432126
H 3.956182 0.538385 -0.916909
H 4.222986 1.499508 -2.389286
H 3.453895 3.641043 -1.142386
H 1.899604 3.602639 -0.274692
H 3.271794 2.636870 0.313235

**E5_CIS_C**
Inner Energy (Hartree) : -1491.824034
Zero Point Energy (Hartree) : 0.494831
Enthalpy (Hartree) : -1491.297964
Entropy (Hartree) : 0.086789
Gibbs Energy (Hartree) : -1491.384752
Imaginary frequency (cm-1): -25.83
N 1.105283 -0.076068 0.184123
N -0.285635 -0.384375 0.178175
C -0.519625 -1.666125 -0.376914
C 1.865407 -1.096908 0.908716
C 3.202008 -1.412749 0.355800
C -1.919957 -2.158287 -0.241656
O 1.379399 -1.644464 1.865416
O 0.369853 -2.336740 -0.854875
C 4.125066 -2.039074 1.194851
C 5.378518 -2.387916 0.707462
C 5.704511 -2.126395 -0.621775
C 4.776423 -1.516355 -1.464203
C 3.526495 -1.155947 -0.977773
C -2.524810 -2.782028 -1.333554
C -3.808104 -3.301020 -1.207417
C -4.471588 -3.228405 0.016691
C -3.854050 -2.632574 1.114682
C -2.581102 -2.086816 0.986343
O 0.756836 2.158951 0.003211
C 1.224471 3.549378 -0.157855

C 1.651004 4.114484 1.193475
C -0.013725 4.266488 -0.677624
C 2.339635 3.594679 -1.199628
C 1.617671 1.212577 0.344229
O 2.753653 1.377431 0.737719
O -2.137096 0.850555 0.560848
C -3.447586 1.447909 0.230192
C -4.135887 0.651711 -0.875386
C -3.263065 2.910548 -0.147838
C -4.203562 1.310469 1.546679
C -1.244017 0.588598 -0.367121
O -1.223436 0.948668 -1.513275
H 3.851194 -2.241724 2.224921
H 6.100199 -2.865904 1.362427
H 6.682673 -2.403041 -1.003999
H 5.027004 -1.325165 -2.502929
H 2.790021 -0.697726 -1.628459
H -1.988638 -2.848492 -2.274785
H -4.288065 -3.769908 -2.060474
H -5.469684 -3.644138 0.116513
H -4.364420 -2.591755 2.072011
H -2.091338 -1.612912 1.830005
H 0.850167 3.980638 1.927626
H 2.555847 3.629718 1.560803
H 1.841778 5.187339 1.086903
H 0.236594 5.305954 -0.909255
H -0.805176 4.262812 0.074744
H -0.385764 3.781356 -1.584264
H 2.584808 4.640247 -1.410745
H 3.240093 3.090273 -0.847762
H 2.004604 3.126488 -2.130734
H -4.229054 -0.401384 -0.597577
H -5.142781 1.057696 -1.014868
H -3.600309 0.728011 -1.822819
H -2.673859 3.010281 -1.060872
H -2.770426 3.454062 0.663398
H -4.245298 3.363612 -0.315015
H -5.199826 1.751335 1.446495
H -4.313524 0.256137 1.815939
H -3.672818 1.826631 2.352083

**E5_GP2_CC**
Inner Energy (Hartree) : -1491.866919
Zero Point Energy (Hartree) : 0.495163
Enthalpy (Hartree) : -1491.339328
Entropy (Hartree) : 0.089781
Gibbs Energy (Hartree) : -1491.429109
Minimum geometry
N -0.270003 -0.346428 -0.152048
N 1.100028 -0.276337 -0.256088
C 1.764737 -1.525979 -0.316039
C -0.992159 -0.381188 -1.373742
C -2.335101 -1.017015 -1.337132
C 3.189791 -1.553512 0.100457
O -0.475518 0.032156 -2.387658
O 1.140093 -2.520633 -0.612631
C -3.351327 -0.487422 -2.130566
C -4.611289 -1.075627 -2.121086
C -4.845856 -2.207059 -1.342067
C -3.818034 -2.755888 -0.575963
C -2.562641 -2.159967 -0.567774
C 3.639125 -0.777380 1.171218
C 4.954209 -0.896676 1.602863
C 5.821942 -1.778129 0.960738
C 5.369959 -2.557842 -0.101975
C 4.049567 -2.457326 -0.523849
O -2.032390 0.365907 1.014674
C -2.912375 0.510301 2.178600
C -2.949450 -0.793787 2.969797
C -4.263029 0.787579 1.527111
C -2.441346 1.693870 3.017322
C -0.779116 -0.033290 1.126577
O -0.101470 -0.112074 2.122222
O 0.822431 1.929024 -0.314448
C 1.031278 3.307610 -0.766207
C 1.138640 3.336141 -2.287889
C 2.259140 3.893263 -0.075850
C -0.246023 3.995038 -0.294295
C 1.675072 0.963122 -0.617954
O 2.775630 1.062075 -1.100426
H -3.150776 0.389199 -2.738134
H -5.409321 -0.654445 -2.724354
H -5.828632 -2.668730 -1.339727
H -3.994690 -3.650539 0.012946
H -1.751082 -2.585816 0.014575
H 2.952009 -0.106480 1.677933
H 5.302729 -0.302320 2.441529
H 6.851913 -1.862452 1.294205
H 6.045077 -3.248250 -0.597661
H 3.677407 -3.068824 -1.339364
H -3.234482 -1.623668 2.315370
H -1.982886 -1.012218 3.427389
H -3.698885 -0.708895 3.762804
H -5.025370 0.926556 2.299523
H -4.556798 -0.049901 0.886307
H -4.215567 1.693920 0.915948
H -3.160974 1.878869 3.821213
H -1.463086 1.495531 3.459087
H -2.377345 2.594257 2.398098
H 0.291378 2.807457 -2.734539
H 1.124194 4.375832 -2.629620
H 2.064090 2.869244 -2.629450
H 3.170293 3.387097 -0.398717
H 2.164263 3.799591 1.010668
H 2.339736 4.956448 -0.323888
H -0.222841 5.053379 -0.570309
H -1.122725 3.530421 -0.755768
H -0.342310 3.919426 0.793259

**E5_CIS_CC**
Inner Energy (Hartree) : -1491.828167
Zero Point Energy (Hartree) : 0.494400
Enthalpy (Hartree) : -1491.302253
Entropy (Hartree) : 0.087882
Gibbs Energy (Hartree) : -1491.390135
Imaginary frequency (cm-1): -24.29
N 0.854602 0.124157 -0.558924
N -0.447330 0.088850 0.026327
C -1.431437 0.655252 -0.809263
C 1.257405 1.408751 -1.137020
C 0.917156 2.626156 -0.348148
C -2.830584 0.605370 -0.301070
O 1.912617 1.427097 -2.142284
O -1.140236 1.195516 -1.858181
C 0.826293 3.839973 -1.033248
C 0.566151 5.012444 -0.336766
C 0.412461 4.979267 1.048650
C 0.517923 3.772741 1.735791
C 0.763748 2.593347 1.040394
C -3.146329 0.877283 1.031583
C -4.477238 0.886175 1.434321
C -5.487880 0.614384 0.515448
C -5.172411 0.349934 -0.816333
C -3.845950 0.359545 -1.228798
O 3.023407 -0.363643 -0.370537
C 4.228998 -0.964090 0.212929
C 4.383711 -0.506395 1.660666
C 5.333417 -0.383011 -0.663580
C 4.169241 -2.483265 0.079117
C 1.825280 -0.608531 0.127747
O 1.550544 -1.357670 1.044821
O -0.727343 -2.123715 -0.384036
C -1.101228 -3.527883 -0.152758
C -2.604505 -3.602630 0.100708
C -0.277777 -4.109601 0.991477
C -0.734029 -4.184016 -1.478791
C -0.846194 -1.220691 0.568615
O -1.320989 -1.333452 1.663094
H 0.952855 3.846144 -2.110622
H 0.483883 5.953043 -0.872093
H 0.210989 5.896690 1.593512
H 0.406715 3.747613 2.815181
H 0.835464 1.654893 1.580655
H -2.354406 1.081056 1.743066
H -4.724565 1.104446 2.468286
H -6.525300 0.614150 0.836244

H -5.960482 0.143927 -1.533696
H -3.584666 0.175549 -2.265944
H 4.349568 0.586393 1.716965
H 3.600064 -0.921889 2.296334
H 5.354932 -0.838871 2.040358
H 6.305828 -0.755635 -0.328212
H 5.336182 0.709159 -0.603710
H 5.186972 -0.673010 -1.707950
H 5.135667 -2.906621 0.370669
H 3.393988 -2.908676 0.718180
H 3.971440 -2.763515 -0.960286
H -3.149831 -3.098324 -0.703548
H -2.914118 -4.652249 0.122890
H -2.871000 -3.139757 1.052349
H -0.529430 -3.640146 1.943614
H 0.789301 -3.967041 0.801172
H -0.482331 -5.182583 1.063839
H -0.975850 -5.250357 -1.442640
H -1.290420 -3.727130 -2.302530
H 0.336316 -4.074443 -1.676962

**E5_GP1**
Inner Energy (Hartree) : -1491.868508
Zero Point Energy (Hartree) : 0.495556
Enthalpy (Hartree) : -1491.340711
Entropy (Hartree) : 0.089174
Gibbs Energy (Hartree) : -1491.429885
Minimum geometry
N -0.673311 0.006220 -0.253060
N 0.694350 0.088276 -0.175904
C 1.346186 0.743735 -1.252994
C -1.179597 -1.146833 -0.898224
C -2.548110 -1.586773 -0.512661
C 2.755751 0.352167 -1.523816
O -0.472384 -1.770858 -1.657865
O 0.725218 1.517686 -1.944585
C -3.409187 -2.067713 -1.497006
C -4.678217 -2.514330 -1.143884
C -5.072043 -2.509188 0.192794
C -4.194988 -2.060893 1.179755
C -2.934165 -1.592951 0.829297
C 3.140004 -0.989468 -1.478736
C 4.449398 -1.340640 -1.784399
C 5.375449 -0.354422 -2.122683
C 4.985075 0.981961 -2.180454
C 3.670338 1.335454 -1.895962
O -2.586041 1.155591 -0.349326
C -3.629445 2.084048 0.101003
C -3.825761 1.961565 1.609387
C -4.856359 1.574396 -0.647562
C -3.257835 3.499402 -0.328091
C -1.377066 1.154641 0.175844
O -0.865929 1.974506 0.898848
O 2.447129 0.206854 1.200801
C 3.364146 -0.232327 2.258805
C 3.545942 -1.745983 2.195057
C 2.835675 0.238659 3.609608
C 4.655736 0.484985 1.881190
C 1.236798 -0.297994 1.068114
O 0.612475 -0.973130 1.851666
H -3.085006 -2.075708 -2.532695
H -5.358324 -2.871221 -1.910844
H -6.060716 -2.864209 0.467568
H -4.493858 -2.076455 2.223296
H -2.239285 -1.248650 1.590208
H 2.407610 -1.750057 -1.224586
H 4.747780 -2.384179 -1.760730
H 6.400615 -0.629753 -2.351340
H 5.704078 1.748387 -2.452144
H 3.349355 2.370938 -1.946299
H -4.039878 0.923044 1.880377
H -2.945415 2.302387 2.157029
H -4.680440 2.576988 1.907143
H -5.723777 2.194235 -0.401382
H -5.070995 0.538103 -0.367394
H -4.691877 1.616852 -1.728311
H -4.088606 4.175903 -0.103063
H -2.367642 3.847148 0.199347
H -3.067712 3.531280 -1.405206
H 3.876043 -2.045407 1.195398
H 4.316037 -2.042497 2.913952
H 2.621325 -2.271224 2.441323
H 1.902632 -0.267822 3.863097
H 2.660847 1.318760 3.592343
H 3.577763 0.020967 4.384433
H 5.438796 0.242456 2.605905
H 4.987222 0.175388 0.884943
H 4.506056 1.568702 1.877792

**E5_TRANS_CC**
Inner Energy (Hartree) : -1491.826906
Zero Point Energy (Hartree) : 0.494378
Enthalpy (Hartree) : -1491.301052
Entropy (Hartree) : 0.087507
Gibbs Energy (Hartree) : -1491.388559
Imaginary frequency (cm-1): -18.14
N 0.255145 0.244365 -0.259796
N 0.131970 -0.749917 0.764882
C 1.368531 -1.143700 1.473758
C -0.993417 0.709286 -0.902601
C -2.094017 1.156446 -0.006487
C 2.512267 -1.570744 0.623293
O -1.029040 0.782478 -2.099469
O 1.348302 -1.214126 2.670027
C -3.319351 1.465502 -0.606733
C -4.388144 1.894737 0.167480
C -4.234609 2.033389 1.546590
C -3.009735 1.750969 2.144944
C -1.938054 1.312027 1.374473
C 2.363564 -1.894292 -0.727618
C 3.462774 -2.334205 -1.457908
C 4.708782 -2.444336 -0.847059
C 4.858146 -2.122300 0.501586
C 3.762275 -1.692017 1.236352
O 0.973657 2.289122 -0.763973
C 1.705394 3.551677 -0.595104
C 1.344406 4.175419 0.750259
C 1.165904 4.392476 -1.747160
C 3.202902 3.305236 -0.750437
C 1.130720 1.283481 0.081752
O 1.906869 1.218573 1.012006
O -1.449448 -1.686035 -0.538449
C -2.586914 -2.569718 -0.837760
C -3.619163 -2.447541 0.278824
C -2.092985 -3.999309 -1.038215
C -3.120592 -1.994661 -2.144660
C -0.723596 -1.839013 0.563150
O -0.752823 -2.769621 1.335905
H -3.414694 1.369603 -1.683166
H -5.338799 2.125991 -0.302373
H -5.070169 2.367313 2.154199
H -2.886024 1.867232 3.216668
H -0.994805 1.079756 1.856321
H 1.398644 -1.803431 -1.215128
H 3.344100 -2.589878 -2.506027
H 5.565754 -2.782934 -1.421638
H 5.829272 -2.209488 0.978599
H 3.857243 -1.441409 2.287460
H 0.256728 4.248372 0.852470
H 1.742462 3.591804 1.581810
H 1.762741 5.185500 0.801008
H 1.641125 5.377814 -1.738773
H 0.083991 4.524317 -1.652753
H 1.375912 3.910045 -2.706085
H 3.724935 4.267353 -0.757510
H 3.593691 2.700555 0.069200
H 3.406431 2.796941 -1.698061
H -3.897501 -1.398308 0.423749
H -4.517415 -3.007625 0.000509
H -3.236039 -2.849714 1.218588
H -1.714059 -4.424148 -0.108471
H -1.298992 -4.022181 -1.791505
H -2.923479 -4.615398 -1.397569
H -3.961500 -2.601139 -2.493871
H -3.467659 -0.968142 -2.002991
H -2.342371 -1.995903 -2.912942

**E5_TRANS_C**
Inner Energy (Hartree)      : -1491.830152
Zero Point Energy (Hartree) : 0.494834
Enthalpy (Hartree)          : -1491.304035
Entropy (Hartree)           : 0.087033
Gibbs Energy (Hartree)      : -1491.391068
Imaginary frequency (cm-1): -18.92
N  -0.529739  -0.022517  -0.222525
N  0.829287  -0.245802  -0.587692
C  1.394256  -1.311578  0.139569
C  -1.086694  1.177378  -0.671302
C  -2.533777  1.406238  -0.379119
C  2.854022  -1.546152  -0.052138
O  -0.417874  2.011181  -1.253796
O  0.712175  -2.011600  0.862624
C  -3.039191  1.347365  0.920133
C  -4.375453  1.651940  1.152966
C  -5.210725  1.993884  0.090739
C  -4.703330  2.057451  -1.205045
C  -3.360187  1.781612  -1.438790
C  3.602328  -1.875340  1.081401
C  4.962938  -2.129718  0.963987
C  5.572771  -2.083443  -0.288673
C  4.821630  -1.782702  -1.421910
C  3.462845  -1.508135  -1.308050
O  -2.193501  -1.354949  0.537396
C  -3.266808  -2.359672  0.471927
C  -4.171805  -2.043857  -0.715017
C  -4.000377  -2.142732  1.790702
C  -2.663854  -3.759995  0.410415
C  -1.327207  -1.230620  -0.438032
O  -1.168198  -1.924166  -1.406401
O  1.669923  1.534071  0.532102
C  2.513777  2.707494  0.805609
C  2.162570  3.842774  -0.150441
C  3.979469  2.289272  0.719817
C  2.126643  3.059748  2.237444
C  1.683422  0.948050  -0.651203
O  2.365665  1.193290  -1.605092
H  -2.389326  1.057339  1.738361
H  -4.767129  1.616879  2.164928
H  -6.256888  2.218618  0.274990
H  -5.350539  2.329918  -2.032582
H  -2.948749  1.846889  -2.441016
H  3.109349  -1.924476  2.046991
H  5.546451  -2.370511  1.847024
H  6.635120  -2.288172  -0.381683
H  5.293652  -1.759646  -2.398913
H  2.876507  -1.263972  -2.186147
H  -4.544655  -1.016429  -0.646213
H  -3.643995  -2.171058  -1.662513
H  -5.028848  -2.724545  -0.703663
H  -4.834801  -2.846209  1.866468
H  -4.395412  -1.124530  1.847254
H  -3.326464  -2.308267  2.636491
H  -3.461736  -4.496471  0.549636
H  -2.176398  -3.945440  -0.547055
H  -1.928931  -3.888164  1.210674
H  1.088167  4.043674  -0.122280
H  2.697577  4.745829  0.160511
H  2.446774  3.603063  -1.176003
H  4.260698  2.034244  -0.303326
H  4.165335  1.423212  1.363391
H  4.610673  3.115987  1.060763
H  2.704753  3.925361  2.574100
H  1.062313  3.306146  2.297200
H  2.330583  2.220782  2.909529

**F6_GP2_C**
Inner Energy (Hartree)      : -1108.520964
Zero Point Energy (Hartree) : 0.387479
Enthalpy (Hartree)          : -1108.107236
Entropy (Hartree)           : 0.077169
Gibbs Energy (Hartree)      : -1108.184405
Minimum geometry
N  -1.039365  1.295577  -0.222747
N  0.143283  0.997864  0.428382
C  0.053747  0.960709  1.846629
C  -1.248460  2.618820  -0.674111
C  -0.167173  3.600219  -0.282041
C  1.309388  0.771255  2.655898
O  -2.212955  2.922825  -1.329409
O  -1.026175  1.145864  2.358547
O  -1.368423  -0.902906  -0.073691
C  -2.114788  -2.159859  0.003817
C  -2.649919  -2.534187  -1.375264
C  -3.217096  -2.037633  1.052253
C  -1.040328  -3.144207  0.456130
C  -1.971604  0.242564  -0.386859
O  -3.106238  0.397186  -0.751403
O  2.057640  -0.185001  0.255245
C  3.242086  -0.759751  -0.402874
C  2.801729  -1.835023  -1.391080
C  3.998699  -1.372381  0.770638
C  4.058260  0.348120  -1.061273
C  1.147776  0.481497  -0.428216
O  1.103835  0.667507  -1.619552
H  -0.047670  3.648458  0.804166
H  0.792344  3.318640  -0.724785
H  -0.466813  4.577816  -0.658860
H  2.154514  1.324533  2.243544
H  1.086307  1.112631  3.667280
H  1.584766  -0.285371  2.683173
H  -3.415164  -1.830919  -1.706345
H  -1.835679  -2.545261  -2.106847
H  -3.087443  -3.536758  -1.331654
H  -4.000342  -1.351386  0.726573
H  -2.799375  -1.675940  1.997153
H  -3.662822  -3.022544  1.223440
H  -0.229959  -3.189693  -0.278127
H  -0.622885  -2.840497  1.421175
H  -1.471356  -4.144201  0.560855
H  3.687221  -2.342207  -1.786766
H  2.177416  -2.579350  -0.886888
H  2.243075  -1.404732  -2.223460
H  4.274199  -0.602755  1.498212
H  4.913046  -1.852203  0.409921
H  3.385443  -2.127061  1.272475
H  4.291817  1.132321  -0.333810
H  5.001374  -0.071396  -1.424844
H  3.525202  0.789134  -1.904807

**F6_CIS_C**
Inner Energy (Hartree)      : -1108.479062
Zero Point Energy (Hartree) : 0.387087
Enthalpy (Hartree)          : -1108.066758
Entropy (Hartree)           : 0.074636
Gibbs Energy (Hartree)      : -1108.141394
Imaginary frequency (cm-1): -40.90
N  1.300932  -1.037269  -0.125661
N  -0.102647  -1.249597  -0.040828
C  -0.448242  -2.544484  -0.458972
C  2.046493  -2.077508  0.632045
C  3.296647  -2.574887  -0.030092
C  -1.918347  -2.863973  -0.559564
O  1.641159  -2.431745  1.703028
O  0.414867  -3.369100  -0.688314
O  1.080081  1.228378  0.024303
C  1.599546  2.606033  -0.071896
C  2.379006  2.769586  -1.373874
C  2.431599  2.934120  1.164572
C  0.330871  3.446180  -0.103834
C  1.919201  0.205873  -0.008309
O  3.131686  0.265628  0.063040
O  -1.947585  -0.114146  0.524602
C  -3.214223  0.621173  0.338762
C  -2.933975  2.107721  0.168209
C  -3.939637  0.348536  1.651426
C  -3.990827  0.048932  -0.843569
C  -1.041337  -0.208044  -0.429793
O  -1.010416  0.358446  -1.489345
H  3.336909  -2.291576  -1.080350
H  4.162640  -2.158087  0.489178
H  3.313703  -3.662408  0.071169
H  -2.380640  -2.312750  -1.384158

H -2.003848 -3.931540 -0.759314
H -2.443298 -2.613227 0.364289
H 3.302401 2.189520 -1.363912
H 1.763903 2.452900 -2.222084
H 2.631174 3.826332 -1.507051
H 3.353340 2.352727 1.189531
H 1.855355 2.730235 2.072670
H 2.683925 3.999406 1.151241
H -0.292872 3.164564 -0.955824
H -0.242811 3.311596 0.816992
H 0.594951 4.503911 -0.194583
H -3.883963 2.651184 0.167054
H -2.328752 2.478203 0.999589
H -2.416817 2.307017 -0.771051
H -4.096756 -0.725196 1.791071
H -4.914744 0.844406 1.643165
H -3.360953 0.729440 2.497800
H -4.219618 -1.008964 -0.686641
H -4.939174 0.587448 -0.932996
H -3.441033 0.167671 -1.779542

**F6_GP2_CC**
Inner Energy (Hartree) : -1108.519787
Zero Point Energy (Hartree) : 0.386888
Enthalpy (Hartree) : -1108.106548
Entropy (Hartree) : 0.077090
Gibbs Energy (Hartree) : -1108.183637
Minimum geometry
N 0.261295 1.223237 0.642254
N -0.261276 1.223220 -0.642253
C 0.382746 2.027840 -1.620834
C -0.382827 2.027744 1.620860
C -1.581192 2.804011 1.122271
C 1.580973 2.804293 -1.122202
O 0.014256 2.086958 2.756399
O -0.014297 2.086983 -2.756391
O 1.523301 -0.439485 -0.145385
C 2.479453 -1.552558 -0.130543
C 2.002254 -2.645467 0.822034
C 3.864958 -1.027221 0.232725
C 2.434266 -2.033995 -1.576790
C 1.220634 0.226308 0.964913
O 1.667353 0.054820 2.067658
O -1.523181 -0.439592 0.145360
C -2.479419 -1.552590 0.130528
C -3.864913 -1.027146 -0.232627
C -2.434176 -2.034100 1.576749
C -2.002346 -2.645480 -0.822134
C -1.220468 0.226162 -0.964949
O -1.667321 0.054790 -2.067657
H -1.329694 3.464178 0.287788
H -2.369968 2.125386 0.784898
H -1.947926 3.398926 1.958300
H 2.369849 2.125793 -0.784813
H 1.947635 3.399275 -1.958215
H 1.329344 3.464412 -0.287720
H 2.051866 -2.321421 1.862125
H 0.971744 -2.930293 0.587522
H 2.636309 -3.528716 0.697148
H 3.891365 -0.658714 1.259685
H 4.155601 -0.219453 -0.446850
H 4.595147 -1.836353 0.132638
H 1.424759 -2.363254 -1.841555
H 2.726853 -1.230816 -2.259571
H 3.122524 -2.873827 -1.709707
H -4.595148 -1.836234 -0.132513
H -4.155454 -0.219382 0.446997
H -3.891371 -0.658603 -1.259571
H -1.424675 -2.363435 1.841441
H -3.122479 -2.873895 1.709666
H -2.726673 -1.230936 2.259586
H -0.971840 -2.930381 -0.587697
H -2.636449 -3.528696 -0.697260
H -2.051998 -2.321372 -1.862204

**F6_CIS_CC**
Inner Energy (Hartree) : -1108.486989
Zero Point Energy (Hartree) : 0.386227
Enthalpy (Hartree) : -1108.075119
Entropy (Hartree) : 0.075659
Gibbs Energy (Hartree) : -1108.150777
Imaginary frequency (cm-1): -37.63
N -0.526609 1.016248 0.117438
N 0.764604 1.253655 -0.416798
C 1.404261 2.345133 0.178259
C -1.409010 2.193681 -0.001335
C -2.310909 2.432511 1.175464
C 2.838131 2.562384 -0.239557
O -1.354773 2.889076 -0.976115
O 0.804987 3.085384 0.933676
O -2.387128 -0.188672 0.161132
C -3.255528 -1.358989 -0.019084
C -2.749478 -2.514966 0.839023
C -3.338752 -1.718013 -1.499760
C -4.597245 -0.846292 0.493677
C -1.101266 -0.217500 -0.170824
O -0.492639 -1.152856 -0.646461
O 1.955514 -0.497646 0.400255
C 2.844979 -1.670402 0.402575
C 2.279442 -2.773823 -0.485998
C 2.826501 -2.089799 1.868107
C 4.243359 -1.235514 -0.026824
C 1.626725 0.113184 -0.724285
O 2.057254 -0.066589 -1.829769
H -3.348507 2.463142 0.835034
H -2.194479 1.675810 1.948888
H -2.049886 3.414096 1.580497
H 2.955073 2.476572 -1.322035
H 3.135392 3.556570 0.092762
H 3.489540 1.821447 0.235427
H -1.790849 -2.888973 0.476102
H -2.638584 -2.195082 1.879968
H -3.478395 -3.330837 0.806856
H -2.383634 -2.088935 -1.874347
H -3.636601 -0.842948 -2.085751
H -4.095519 -2.496794 -1.637067
H -4.518620 -0.542326 1.541847
H -4.931507 0.012466 -0.096211
H -5.349460 -1.636633 0.415122
H 2.894251 -3.671312 -0.363689
H 1.252478 -3.007190 -0.195437
H 2.288701 -2.484468 -1.537891
H 3.180450 -1.275721 2.507749
H 3.478903 -2.955987 2.012959
H 1.812148 -2.360786 2.175417
H 4.611907 -0.433619 0.621567
H 4.928049 -2.084764 0.062411
H 4.250184 -0.892181 -1.063036

**F6_GP1**
Inner Energy (Hartree) : -1108.530497
Zero Point Energy (Hartree) : 0.387629
Enthalpy (Hartree) : -1108.116642
Entropy (Hartree) : 0.076870
Gibbs Energy (Hartree) : -1108.193513
Minimum geometry
N -0.314498 1.176694 0.613743
N 0.314487 1.176691 -0.613754
C -0.214884 2.093475 -1.557222
C 0.214831 2.093529 1.557185
C -0.450187 2.208302 2.899303
C 0.450036 2.208130 -2.899399
O 1.179912 2.748619 1.236847
O -1.179936 2.748599 -1.236869
O -1.523331 -0.468548 -0.277823
C -2.498185 -1.562815 -0.331499
C -3.908516 -1.010670 -0.147869
C -2.140745 -2.623548 0.706039
C -2.301451 -2.098639 -1.745975
C -1.354882 0.254136 0.818988
O -1.962936 0.160368 1.859718
O 1.523293 -0.468567 0.277815
C 2.498197 -1.562790 0.331513
C 2.140905 -2.623479 -0.706119
C 2.301369 -2.098703 1.745942
C 3.908514 -1.010557 0.148039

C 1.354967 0.254228 -0.818941
O 1.963063 0.160488 -1.859650
H -1.509406 2.450118 2.793899
H -0.389446 1.265556 3.446891
H 0.069253 2.996579 3.444723
H 0.389414 1.265293 -3.446840
H -0.069545 2.996252 -3.444909
H 1.509225 2.450124 -2.794084
H -4.058437 -0.632066 0.864199
H -4.093404 -0.202605 -0.862410
H -4.633777 -1.808790 -0.335328
H -2.280346 -2.251310 1.721965
H -1.099538 -2.939134 0.581466
H -2.782489 -3.498223 0.561578
H -2.512116 -1.319460 -2.484457
H -1.272571 -2.444000 -1.887404
H -2.979257 -2.939050 -1.921945
H 2.782692 -3.498122 -0.561664
H 1.099707 -2.939140 -0.581654
H 2.280564 -2.251165 -1.722010
H 2.511943 -1.319558 2.484486
H 2.979194 -2.939096 1.921919
H 1.272492 -2.444113 1.887270
H 4.093288 -0.202512 0.862632
H 4.633801 -1.808645 0.335531
H 4.058510 -0.631901 -0.863998

**G7_GP2_C**
Inner Energy (Hartree) : -1190.260137
Zero Point Energy (Hartree) : 0.484934
Enthalpy (Hartree) : -1189.746187
Entropy (Hartree) : 0.082954
Gibbs Energy (Hartree) : -1189.829141
Minimum geometry
N -0.539914 -0.152256 -0.484898
N -0.233066 -0.644846 0.765074
C -1.018089 -0.164701 1.903932
C -1.453890 -0.933039 -1.307861
C -2.893435 -0.752529 -0.881195
C -0.962415 1.325918 2.095295
C -0.504203 1.916129 3.194941
C -3.644047 -1.820771 -0.396085
C -4.957669 -1.626109 0.029776
C -5.526501 -0.357907 -0.025006
C -4.780269 0.716004 -0.511168
C -3.472542 0.518743 -0.937474
O 1.084691 1.343291 -0.168475
C 1.813055 2.586846 -0.394816
C 0.834101 3.755912 -0.477890
C 2.681160 2.464552 -1.644559
C 2.671314 2.697141 0.861812
C 0.148340 0.918417 -1.015002
O -0.108342 1.380544 -2.112769
O 1.519937 -1.613817 -0.211133
C 2.855109 -2.194911 -0.282758
C 3.104961 -2.258166 -1.786925
C 2.855227 -3.594580 0.327309
C 3.863098 -1.265655 0.389218
C 0.953463 -1.320594 0.962414
O 1.369366 -1.618434 2.065726
H -2.056380 -0.477239 1.745076
H -0.632157 -0.684437 2.780996
H -1.311950 -0.597218 -2.336687
H -1.152541 -1.983170 -1.243190
H -1.365026 1.928027 1.282081
H -0.515097 2.996877 3.306969
H -0.102353 1.340137 4.025432
H -3.196983 -2.810409 -0.341662
H -5.532456 -2.465671 0.409453
H -6.547918 -0.203794 0.309914
H -5.221068 1.707431 -0.558538
H -2.885318 1.351722 -1.316813
H 1.394437 4.694468 -0.535501
H 0.195133 3.675441 -1.358872
H 0.208191 3.785411 0.420058
H 2.066986 2.380929 -2.542247
H 3.326594 1.582737 -1.572716
H 3.320351 3.349210 -1.731413
H 3.263818 3.616580 0.829686
H 2.035770 2.713544 1.752106
H 3.353499 1.845378 0.940952
H 3.054117 -1.256704 -2.225559
H 4.096122 -2.677604 -1.984244
H 2.355761 -2.889218 -2.274780
H 2.672423 -3.553580 1.401869
H 3.826121 -4.068590 0.149833
H 2.081873 -4.210788 -0.142794
H 4.876572 -1.642771 0.219409
H 3.790183 -0.263344 -0.044615
H 3.686338 -1.200508 1.464019

**G7_CIS_C**
Inner Energy (Hartree) : -1190.210020
Zero Point Energy (Hartree) : 0.484575
Enthalpy (Hartree) : -1189.697569
Entropy (Hartree) : 0.080131
Gibbs Energy (Hartree) : -1189.777700
Imaginary frequency (cm-1): -52.57
N -0.710138 0.291478 -0.696170
N 0.060370 -0.909487 -0.548170
C -0.707115 -1.931417 0.207892
C -1.856532 0.096345 -1.592954
C -3.175641 -0.146286 -0.888760
C -1.134957 -1.564332 1.600971
C -0.987444 -2.366875 2.649966
C -3.994064 -1.209819 -1.267812
C -5.215842 -1.427538 -0.634348
C -5.628438 -0.580968 0.389768
C -4.817573 0.487109 0.770532
C -3.601387 0.706770 0.132604
O 1.014882 1.679691 -0.227839
C 1.541577 2.929895 0.329480
C 0.719421 3.333933 1.551055
C 1.568355 4.011478 -0.747571
C 2.957251 2.551145 0.746478
C -0.269743 1.584324 -0.560142
O -1.021181 2.526504 -0.767316
O 2.226838 -1.367810 -0.813845
C 3.558458 -1.830255 -0.409503
C 4.105422 -2.425333 -1.703570
C 3.416112 -2.907082 0.664240
C 4.422224 -0.662871 0.053626
C 1.376081 -0.846995 0.061927
O 1.612777 -0.543549 1.203910
H -1.586521 -2.177246 -0.394694
H -0.078985 -2.828769 0.216488
H -1.937720 0.994912 -2.208621
H -1.606718 -0.742272 -2.249572
H -1.629542 -0.601684 1.709086
H -1.352601 -2.089983 3.634546
H -0.495080 -3.333851 2.565579
H -3.671051 -1.879960 -2.061748
H -5.840102 -2.262958 -0.937439
H -6.577190 -0.751351 0.889878
H -5.136270 1.154488 1.565979
H -2.973175 1.545985 0.416137
H 1.205938 4.178193 2.049738
H -0.292280 3.631411 1.270787
H 0.667227 2.498827 2.257033
H 0.558859 4.294154 -1.046658
H 2.113496 3.653233 -1.626888
H 2.087190 4.894227 -0.359160
H 3.456475 3.420273 1.185553
H 2.933295 1.743698 1.482256
H 3.536993 2.220182 -0.119819
H 4.162351 -1.660305 -2.483702
H 5.109514 -2.825490 -1.533662
H 3.461267 -3.235856 -2.057120
H 3.010405 -2.495769 1.590331
H 4.399822 -3.337581 0.876091
H 2.760899 -3.710537 0.311396
H 5.447576 -1.014421 0.207658
H 4.439753 0.120595 -0.709845
H 4.051032 -0.242119 0.988229

**G7_GP2_CC**

Inner Energy (Hartree) : -1190.259205
Zero Point Energy (Hartree) : 0.485058
Enthalpy (Hartree) : -1189.745244
Entropy (Hartree) : 0.082492
Gibbs Energy (Hartree) : -1189.827736
Minimum geometry
N -0.524814 0.869020 -0.506698
N -0.127155 -0.315320 0.076157
C -0.519493 -0.557638 1.472986
C -1.579329 0.783511 -1.507168
C -2.918832 0.466757 -0.883251
C -1.311720 -1.823815 1.650279
C -2.592149 -1.842165 2.005729
C -3.613546 -0.690996 -1.221984
C -4.847531 -0.974214 -0.638811
C -5.393242 -0.100370 0.295537
C -4.701121 1.059873 0.644307
C -3.472606 1.339785 0.057180
O 1.293264 1.752617 0.429150
C 2.458429 2.628950 0.490079
C 3.350612 1.914923 1.501406
C 2.053747 4.009501 1.000118
C 3.133235 2.681704 -0.878681
C 0.313781 1.965739 -0.453876
O 0.124000 2.977442 -1.100473
O 1.355177 -1.959495 0.239587
C 2.447406 -2.831923 -0.182487
C 2.073941 -3.554917 -1.474543
C 2.550692 -3.818761 0.976755
C 3.735396 -2.022984 -0.319008
C 0.933730 -0.951918 -0.531665
O 1.381735 -0.626511 -1.614313
H 0.381037 -0.570738 2.097096
H -1.121513 0.305541 1.761087
H -1.603251 1.751913 -2.010098
H -1.301135 0.023998 -2.245987
H -0.774557 -2.752526 1.471637
H -3.134326 -2.775278 2.130183
H -3.153350 -0.924465 2.167167
H -3.183470 -1.381861 -1.942687
H -5.377951 -1.881937 -0.911067
H -6.353662 -0.319733 0.752602
H -5.123206 1.747944 1.371064
H -2.931235 2.243567 0.327097
H 4.273754 2.482412 1.652880
H 2.838948 1.817338 2.463946
H 3.609874 0.914807 1.140419
H 1.532790 3.920982 1.959079
H 1.400743 4.512909 0.285976
H 2.950954 4.618624 1.151699
H 4.100630 3.186052 -0.788670
H 3.299751 1.665477 -1.249969
H 2.520967 3.223760 -1.601283
H 2.003206 -2.857762 -2.310382
H 2.837863 -4.305024 -1.703072
H 1.114256 -4.068359 -1.355396
H 2.762978 -3.292166 1.912179
H 3.357869 -4.532786 0.787579
H 1.615170 -4.374650 1.093758
H 4.569873 -2.700109 -0.528044
H 3.659219 -1.297320 -1.130194
H 3.949774 -1.495178 0.616174

**G7_CIS_CC**
Inner Energy (Hartree) : -1190.217004
Zero Point Energy (Hartree) : 0.485276
Enthalpy (Hartree) : -1189.704312
Entropy (Hartree) : 0.078601
Gibbs Energy (Hartree) : -1189.782913
Imaginary frequency (cm-1): -32.80
N 0.013232 1.153371 0.646540
N -0.131693 1.047570 -0.784044
C -0.480895 2.374475 -1.349833
C -1.228912 1.522702 1.335700
C -2.281492 0.440443 1.271246
C 0.295993 3.543830 -0.809640
C -0.284728 4.652704 -0.360983
C -3.264645 0.452591 0.282292
C -4.174096 -0.596027 0.171375
C -4.113248 -1.665819 1.060115
C -3.145942 -1.678052 2.063246
C -2.236410 -0.630654 2.168592
O 1.706902 -0.314765 0.610245
C 2.953121 -0.909355 1.094080
C 3.535952 -1.536723 -0.168468
C 2.645234 -1.975631 2.141445
C 3.863839 0.193475 1.624726
C 0.920332 0.426876 1.385150
O 0.956889 0.474267 2.603802
O 0.925078 -0.627627 -2.090945
C -0.232973 -1.526062 -2.128401
C 0.284427 -2.655198 -3.018027
C -1.424124 -0.835348 -2.787980
C -0.566125 -2.055399 -0.740457
C 1.035995 0.554837 -1.507853
O 2.013834 1.232284 -1.716063
H -0.332494 2.281087 -2.430578
H -1.554840 2.534473 -1.206842
H -0.935639 1.723559 2.364960
H -1.597093 2.460201 0.916085
H 1.376963 3.441788 -0.822560
H 0.297354 5.496933 -0.003829
H -1.367988 4.763084 -0.340518
H -3.317799 1.286243 -0.413748
H -4.929280 -0.576896 -0.608873
H -4.821139 -2.485036 0.975685
H -3.099658 -2.505758 2.764745
H -1.469524 -0.645782 2.938054
H 4.497590 -2.004347 0.063933
H 2.861588 -2.302161 -0.564302
H 3.683811 -0.776848 -0.940314
H 1.930427 -2.702430 1.741577
H 2.229182 -1.531216 3.046116
H 3.567929 -2.507533 2.395725
H 4.841147 -0.231169 1.874897
H 4.004708 0.961243 0.857606
H 3.444048 0.654203 2.521138
H 1.171843 -3.115824 -2.573483
H -0.487200 -3.422625 -3.131372
H 0.549596 -2.275568 -4.009230
H -1.849609 -0.073412 -2.133980
H -2.198341 -1.580152 -2.999162
H -1.121429 -0.377937 -3.735860
H -1.351987 -2.812842 -0.826556
H 0.313441 -2.515761 -0.281815
H -0.935809 -1.265349 -0.089225

**G7_GP1**
Inner Energy (Hartree) : -1190.265315
Zero Point Energy (Hartree) : 0.485386
Enthalpy (Hartree) : -1189.751231
Entropy (Hartree) : 0.081634
Gibbs Energy (Hartree) : -1189.832865
Minimum geometry
N 0.339144 -1.412054 0.005231
N 0.363119 -0.930732 1.297388
C 1.236593 -1.628576 2.239364
C -0.717413 -2.364664 -0.326536
C -2.072343 -1.727650 -0.532599
C 2.693434 -1.315646 2.042011
C 3.609306 -2.223534 1.719373
C -2.954362 -1.576139 0.537371
C -4.192787 -0.966838 0.353343
C -4.562518 -0.509074 -0.909247
C -3.687362 -0.660102 -1.983445
C -2.446907 -1.261921 -1.794291
O 1.890848 0.110904 -0.505504
C 2.863770 0.793046 -1.349309
C 3.598233 1.684719 -0.352257
C 2.143221 1.632501 -2.401463
C 3.824122 -0.221091 -1.967075
C 1.077730 -0.822020 -0.998028
O 0.970826 -1.154744 -2.164911
O -0.695601 0.892525 0.551360
C -1.267272 2.232563 0.622142
C -1.843536 2.419750 -0.777353

C  -0.168033  3.256313  0.896293
C  -2.376415  2.270003  1.670404
C  -0.074862  0.347829  1.592368
O  0.103202  0.850174  2.686948
H  0.912681  -1.333359  3.239553
H  1.054238  -2.701782  2.122727
H  -0.395555  -2.888930  -1.226905
H  -0.758304  -3.089796  0.492380
H  2.970392  -0.272756  2.180445
H  4.656208  -1.960071  1.597744
H  3.346220  -3.268517  1.568496
H  -2.667453  -1.935187  1.522813
H  -4.871450  -0.855241  1.194075
H  -5.530901  -0.040053  -1.056688
H  -3.971250  -0.306040  -2.970232
H  -1.754819  -1.367197  -2.624978
H  4.342604  2.294044  -0.873674
H  2.896780  2.350907  0.159158
H  4.110194  1.076605  0.399712
H  1.443782  2.323896  -1.920150
H  1.591997  0.998695  -3.097588
H  2.875798  2.221816  -2.962423
H  4.645914  0.309783  -2.458380
H  4.243998  -0.860225  -1.183419
H  3.318749  -0.847441  -2.703416
H  -2.610902  1.666968  -0.977505
H  -2.290495  3.414897  -0.865468
H  -1.057004  2.320806  -1.532271
H  0.262586  3.118657  1.889125
H  -0.586988  4.265442  0.828141
H  0.625723  3.164272  0.147739
H  -2.887845  3.237009  1.623258
H  -3.107151  1.481350  1.464643
H  -1.973376  2.133276  2.675211

**G7_TRANS_CC**
Inner Energy (Hartree)      : -1190.219109
Zero Point Energy (Hartree) : 0.485687
Enthalpy (Hartree)          : -1189.705945
Entropy (Hartree)           : 0.079317
Gibbs Energy (Hartree)      : -1189.785261
Imaginary frequency (cm-1): -24.36
N  0.463655  -0.132088  -1.515408
N  -0.519646  -0.580696  -0.563179
C  -0.315802  -1.849505  0.169308
C  0.367026  1.333997  -1.817634
C  0.530042  2.186389  -0.580523
C  -0.131121  -1.625491  1.643087
C  -0.749054  -2.339033  2.578747
C  -0.584347  2.615290  0.142832
C  -0.429416  3.343397  1.319145
C  0.845705  3.654426  1.784488
C  1.964427  3.243628  1.062569
C  1.805260  2.516733  -0.113184
O  2.130239  -0.876550  -0.090478
C  3.484827  -1.335167  0.244371
C  3.333968  -1.807288  1.687082
C  4.461491  -0.165005  0.165174
C  3.881733  -2.495956  -0.663691
C  1.829157  -0.436138  -1.307733
O  2.630911  -0.231986  -2.195849
O  -2.632632  -0.754527  0.077239
C  -4.073295  -0.533433  0.049575
C  -4.372287  0.963718  0.102326
C  -4.530467  -1.217826  1.335259
C  -4.694865  -1.208657  -1.170981
C  -1.833255  -0.289028  -0.891979
O  -2.210343  0.327984  -1.871266
H  -1.183151  -2.488324  -0.002904
H  0.542484  -2.361911  -0.260785
H  1.165819  1.516053  -2.534696
H  -0.583528  1.514599  -2.307199
H  0.556819  -0.828897  1.912566
H  -0.573934  -2.163710  3.636074
H  -1.456295  -3.125369  2.322673
H  -1.580164  2.381618  -0.224517
H  -1.305773  3.668860  1.872177
H  0.967747  4.222471  2.702049
H  2.961740  3.495954  1.411484
H  2.679167  2.204328  -0.679571
H  4.292227  -2.191961  2.048878
H  3.026575  -0.979660  2.333546
H  2.585577  -2.601762  1.762962
H  4.100741  0.665879  0.779999
H  4.586336  0.177855  -0.862544
H  5.434583  -0.482728  0.553401
H  4.838212  -2.905521  -0.323941
H  3.133008  -3.293580  -0.611192
H  3.988661  -2.173428  -1.700097
H  -4.066036  1.459048  -0.820356
H  -5.447265  1.115573  0.243301
H  -3.847198  1.421879  0.947261
H  -4.281593  -2.283350  1.311652
H  -5.613882  -1.115017  1.448880
H  -4.040789  -0.767242  2.203639
H  -5.785923  -1.150902  -1.100140
H  -4.376132  -0.725535  -2.095400
H  -4.409854  -2.265390  -1.203674

**G7_TRANS_C**
Inner Energy (Hartree)      : -1190.217402
Zero Point Energy (Hartree) : 0.485648
Enthalpy (Hartree)          : -1189.704203
Entropy (Hartree)           : 0.079333
Gibbs Energy (Hartree)      : -1189.783536
Imaginary frequency (cm-1): -24.61
N  -0.371116  -0.282487  0.722102
N  0.064853  -0.690977  -0.591535
C  -0.850658  -1.510187  -1.409601
C  0.467263  0.753177  1.376918
C  0.931106  1.861863  0.456435
C  -1.306254  -2.757539  -0.699921
C  -2.526732  -3.273937  -0.800208
C  2.191704  2.425053  0.651649
C  2.630013  3.486671  -0.136495
C  1.809086  3.991460  -1.140125
C  0.549012  3.432395  -1.344518
C  0.111896  2.377724  -0.550445
O  -2.530051  0.028015  -0.044584
C  -3.961678  0.310440  0.110560
C  -4.463131  0.262543  -1.329958
C  -4.138101  1.711568  0.690874
C  -4.641896  -0.761660  0.957290
C  -1.728439  -0.059140  1.013588
O  -2.079503  0.103936  2.165317
O  2.094181  -1.089938  0.393937
C  3.525306  -1.405296  0.399124
C  3.916917  -1.135659  1.849586
C  4.285892  -0.477025  -0.544609
C  3.724651  -2.879740  0.056831
C  1.388529  -1.130651  -0.731116
O  1.814766  -1.504489  -1.808442
H  -1.687187  -0.915118  -1.760716
H  -0.247027  -1.782214  -2.275494
H  -0.162252  1.165524  2.165748
H  1.319726  0.285950  1.861684
H  -0.545643  -3.270492  -0.111237
H  -2.793379  -4.208562  -0.316202
H  -3.303041  -2.786894  -1.385984
H  2.838317  2.031426  1.432453
H  3.614861  3.912905  0.030513
H  2.149218  4.813493  -1.762741
H  -0.095897  3.818100  -2.128667
H  -0.866624  1.939773  -0.726069
H  -5.528507  0.509275  -1.359771
H  -3.921585  0.983502  -1.950167
H  -4.329283  -0.735650  -1.757532
H  -3.603726  2.446201  0.079334
H  -3.766993  1.762241  1.715158
H  -5.201352  1.972159  0.688156
H  -5.726327  -0.620874  0.903575
H  -4.403431  -1.757454  0.572521
H  -4.329731  -0.702279  2.000014
H  3.308051  -1.737386  2.531091
H  4.969374  -1.391808  2.003419
H  3.781195  -0.078867  2.099985

H 4.056055 -0.694817 -1.587774
H 5.360829 -0.608818 -0.383596
H 4.030805 0.566923 -0.337124
H 4.780317 -3.140754 0.183457
H 3.132685 -3.507942 0.730421
H 3.432338 -3.085857 -0.973412

**Bibliography**


1. Rasmussen, L.K. Facile Synthesis of Mono-, Di-, and Trisubstituted Alpha-Unbranched Hydrazines. *J. Org. Chem.* **2006**, *71*, 3627–3629, doi:10.1021/jo0525783.
2. Bredihhin, A.; Mäeorg, U. Effective Strategy for the Systematic Synthesis of Hydrazine Derivatives. *Tetrahedron* **2008**, *64*, 6788–6793, doi:10.1016/j.tet.2008.04.096.
3. Comparison of Dynamic HPLC and Dynamic NMR in the Study of Conformational Stereodynamics: Case of the Enantiomers of a Hindered Secondary Phosphine Oxide1 | Journal of the American Chemical Society Available online: https://pubs.acs.org/doi/10.1021/ja9941779 (accessed on 25 September 2023).
4. Gasparrini, F.; D'Acquarica, I.; Pierini, M.; Villani, C. Chromatographic Resolution and Enantiomerization Barriers of Axially Chiral 1-Naphthamides. *J. Sep. Sci.* **2001**, *24*, 941–946, doi:10.1002/1615-9314(20011201)24:12<941::AID-JSSC941>3.0.CO;2-V.
5. Sabia, R.; Ciogli, A.; Pierini, M.; Franzini, R.; Iazzetti, A.; Villani, C. Chromatographic Separation of the Interconverting Enantiomers of Imidazo- and Triazole-Fused Benzodiazepines. *J. Chromatogr. A* **2021**, *1647*, 462148, doi:10.1016/j.chroma.2021.462148.
6. Humphrey, W.; Dalke, A.; Schulten, K. VMD: Visual Molecular Dynamics. *J. Mol. Graph.* **1996**, *14*, 33–38, doi:10.1016/0263-7855(96)00018-5.
7. Pettersen, E.F.; Goddard, T.D.; Huang, C.C.; Couch, G.S.; Greenblatt, D.M.; Meng, E.C.; Ferrin, T.E. UCSF Chimera?A Visualization System for Exploratory Research and Analysis. *J. Comput. Chem.* **2004**, *25*, 1605–1612, doi:10.1002/jcc.20084.
8. Pracht, P.; Bohle, F.; Grimme, S. Automated Exploration of the Low-Energy Chemical Space with Fast Quantum Chemical Methods. *Phys. Chem. Chem. Phys.* **2020**, *22*, 7169–7192, doi:10.1039/C9CP06869D.
9. Bannwarth, C.; Caldeweyher, E.; Ehlert, S.; Hansen, A.; Pracht, P.; Seibert, J.; Spicher, S.; Grimme, S. Extended TIGHT-BINDING Quantum Chemistry Methods. *WIREs Comput. Mol. Sci.* **2021**, *11*, doi:10.1002/wcms.1493.
10. Grimme, S.; Bohle, F.; Hansen, A.; Pracht, P.; Spicher, S.; Stahn, M. Efficient Quantum Chemical Calculation of Structure Ensembles and Free Energies for Nonrigid Molecules. *J. Phys. Chem. A* **2021**, *125*, 4039–4054, doi:10.1021/acs.jpca.1c00971.
11. Bannwarth, C.; Ehlert, S.; Grimme, S. GFN2-xTB—An Accurate and Broadly Parametrized Self-Consistent Tight-Binding Quantum Chemical Method with Multipole Electrostatics and Density-Dependent Dispersion Contributions. *J. Chem. Theory Comput.* **2019**, *15*, 1652–1671, doi:10.1021/acs.jctc.8b01176.
12. Neese, F. Software Update: The ORCA Program System—Version 5.0. *WIREs Comput. Mol. Sci.* *n/a*, e1606, doi:10.1002/wcms.1606.
13. Brandenburg, J.G.; Bannwarth, C.; Hansen, A.; Grimme, S. B97-3c: A Revised Low-Cost Variant of the B97-D Density Functional Method. *J. Chem. Phys.* **2018**, *148*, 064104, doi:10.1063/1.5012601.
14. Grimme, S.; Hansen, A.; Ehlert, S.; Mewes, J.-M. r2SCAN-3c: A "Swiss Army Knife" Composite Electronic-Structure Method. *J. Chem. Phys.* **2021**, *154*, 064103, doi:10.1063/5.0040021.
15. Grimme, S. Supramolecular Binding Thermodynamics by Dispersion-Corrected Density Functional Theory. *Chem. – Eur. J.* **2012**, *18*, 9955–9964, doi:10.1002/chem.201200497.
16. Ehlert, S.; Stahn, M.; Spicher, S.; Grimme, S. Robust and Efficient Implicit Solvation Model for Fast Semiempirical Methods. *J. Chem. Theory Comput.* **2021**, *17*, 4250–4261, doi:10.1021/acs.jctc.1c00471.

17. Spicher, S.; Grimme, S. Single-Point Hessian Calculations for Improved Vibrational Frequencies and Rigid-Rotor-Harmonic-Oscillator Thermodynamics. *J. Chem. Theory Comput.* **2021**, *17*, 1701–1714, doi:10.1021/acs.jctc.0c01306.
18. M. J. Frisch; G. W. Trucks; H. B. Schlegel; G. E. Scuseria; M. A. Robb; J. R. Cheeseman; G. Scalmani; V. Barone; G. A. Petersson; H. Nakatsuji; et al. Gaussian 16, Revision A.03. *Gaussian Inc* **2016**.
19. Portolani, C.; Centonze, G.; Luciani, S.; Pellegrini, A.; Righi, P.; Mazzanti, A.; Ciogli, A.; Sorato, A.; Bencivenni, G. Synthesis of Atropisomeric Hydrazides by One-Pot Sequential Enantio- and Diastereoselective Catalysis. *Angew. Chem. Int. Ed.* **2022**, doi:10.1002/anie.202209895.
20. Contreras-García, J.; Johnson, E.R.; Keinan, S.; Chaudret, R.; Piquemal, J.-P.; Beratan, D.N.; Yang, W. NCIPLOT: A Program for Plotting Noncovalent Interaction Regions. *J. Chem. Theory Comput.* **2011**, *7*, 625–632, doi:10.1021/ct100641a.
21. Zimmerman, P.M. Growing String Method with Interpolation and Optimization in Internal Coordinates: Method and Examples. *J. Chem. Phys.* **2013**, *138*, 184102, doi:10.1063/1.4804162.
22. Luchini, G.; Alegre-Requena, J.V.; Funes-Ardoiz, I.; Paton, R.S. GoodVibes: Automated Thermochemistry for Heterogeneous Computational Chemistry Data 2020.
23. Simón, L.; Goodman, J.M. How Reliable Are DFT Transition Structures? Comparison of GGA, Hybrid-Meta-GGA and Meta-GGA Functionals. *Org Biomol Chem* **2011**, *9*, 689–700, doi:10.1039/C0OB00477D.
24. Conformer-Rotamer Ensemble Sampling Tool 2022.
25. Zhao, Q.; Hsu, H.-H.; Savoie, B. Conformational Sampling for Transition State Searches on a Computational Budget. **2021**, doi:10.26434/chemrxiv-2021-xhxtc.
26. Velasco-Juárez, E.; Arpa, E.M. A Novel Partitioning Scheme for the Application of the Distortion/Interaction - Activation Strain Model to Intramolecular Reactions. *Theor. Chem. Acc.* **2021**, *140*, 107, doi:10.1007/s00214-021-02803-3.
27. Houk, K.N.; Bickelhaupt, M. Analyzing Reaction Rates with the Distortion Interaction-Activation Strain Model. *Angew. Chem. Int. Ed.* **2017**, *56*, 10070–10086, doi:10.1002/anie.201701486.
